# Donor-free 9,10-dihydro-9,10-dialuminaanthracenes†

**DOI:** 10.1039/d4sc06940d

**Published:** 2024-11-18

**Authors:** Paula L. Lückert, Jannik Gilmer, Alexander Virovets, Hans-Wolfram Lerner, Matthias Wagner

**Affiliations:** a Institut für Anorganische und Analytische Chemie, Goethe-Universität Frankfurt Max-von-Laue-Straße 7 D-60438 Frankfurt (Main) Germany matthias.wagner@chemie.uni-frankfurt.de

## Abstract

Despite their promising potential, *e.g.*, as ditopic, cooperatively binding Lewis acids, 9,10-dihydro-9,10-dialuminaanthracenes (DAA-R_2_; R: terminal Al-bonded substituent) have remained unexplored for long due to the challenges in synthesizing the ligand-free species. We demonstrate that DAA-Me_2_ is accessible *via* the reaction of 1,2-(Me_3_Sn)_2_C_6_H_4_ with AlMe_3_, producing volatile SnMe_4_ as the sole byproduct. In non-coordinating solvents and in the solid state, DAA-Me_2_ exists as a dimer (DAA-Me_2_)_2_. Treatment of (DAA-Me_2_)_2_ with 4 equiv. AlBr_3_ cleaves the dimer, leads to quantitative Me/Br exchange, and forms the double AlBr_3_ adduct DAA-Br_2_·(AlBr_3_)_2_. Removal of AlBr_3_ with 2,2′-bipyridine gives free DAA-Br_2_, which also dimerizes in the absence of bases to form (DAA-Br_2_)_2_. (DAA-Me_2_)_2_ and (DAA-Br_2_)_2_ readily react with mono- (*e.g.*, pyridine) or ditopic Lewis bases (*e.g.*, potassium pyrazolide) to afford *trans*-diadducts or triptycene-type frameworks. Upon addition of [*n*Bu_4_N]Br, DAA-Br_2_·(AlBr_3_)_2_ undergoes selective cleavage of Al–C bonds to produce the Br^−^ chelate complex of 1,2-(Br_2_Al)_2_C_6_H_4_, a valuable synthon for 1,2-dideprotonated benzenes.

## Abstract 2

Donor-free 9,10-dialuminaanthracenes were prepared and fully characterized with Al-bonded Me or Br substituents. The low steric demand of the Me group ensures high reactivity of the unprotected Al centers: the Al–Br bonds allow late derivatizations.

## Graphical abstract

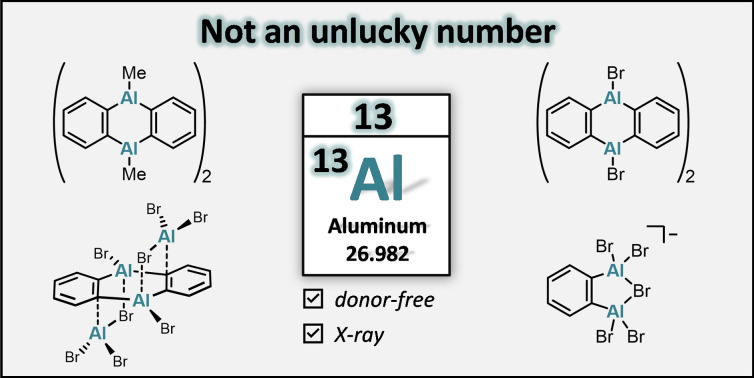

## Introduction

**Fig. 1:**
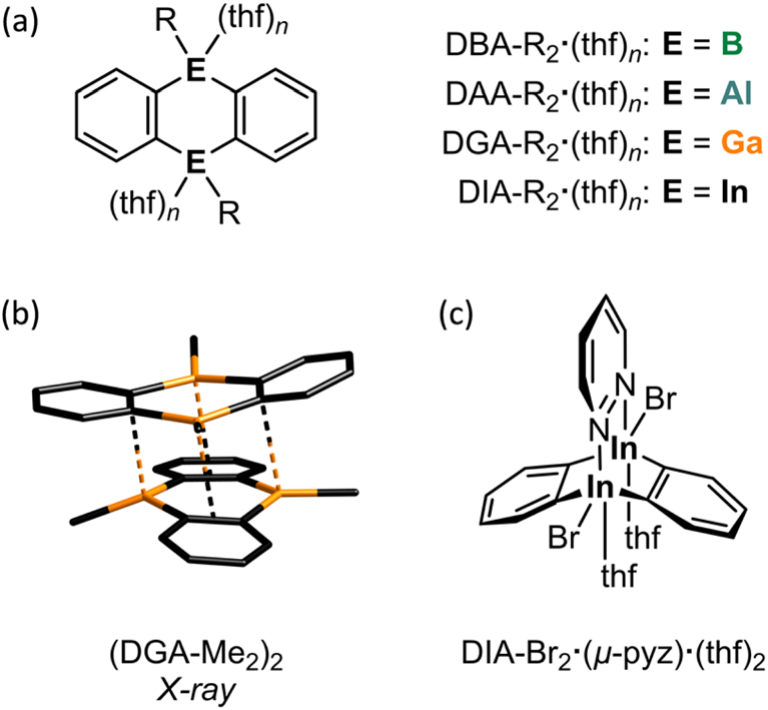
(a) General structures of thf adducts of heteroanthracenes (E = B, Al, Ga, or In). (b) Solid-state structure of (DGA-Me_2_)_2_ with all H atoms omitted for clarity. (c) Triptycene-type structure of DIA-Br_2_·(μ-pyz)·(thf)_2_, where pyz = pyridazine.

The incorporation of p-block elements other than carbon into polycyclic aromatic hydrocarbons (‘heteroatom doping’) has emerged as a powerful tool for imparting new and valuable chemical and physical properties to these compounds.^1^ Notable examples are 9,10-dihydro-9,10-diboraanthracenes (DBAs; Fig. 1a, E = B), which have found wide-ranging applications,^2^ including their use as fluorophores^3^ or homogeneous catalysts.^4^ To further enhance the utility of DBAs, various substituted derivatives have been developed,^5,6^ the B-doped acene scaffold has been expanded by benzannulation,^7^ and additional heteroatoms (such as N, O, and S) have been introduced into the delocalized π system.^8^

Significantly less attention has been given to what is arguably the most impactful modification: the exchange of the B atoms for their higher homologues.^9,10^ While a few anthracenes containing Al (DAAs),^11,12^ Ga (DGAs),^11,13^ or In (DIAs)^11,14–18^ at the 9,10-positions are known, these compounds are typically isolated as their Lewis-base adducts, which inherently diminishes the desired reactivity. As an example, the synthesis of DAA-Me_2_·(thf)_*n*_ according to Bickelhaupt *et al.*^11^ uses MeAlCl_2_ (ref. 19) and [Mg(thf)(*o*-C_6_H_4_)]_4_, prepared from [Hg(*o*-C_6_H_4_)]_3_,^20,21^ in THF;^11,22^ DGA-Me_2_·(thf)_*n*_ and DIA-Me_2_·(thf)_*n*_ were synthesized in a similar manner (Fig. 1a, E = Al, Ga, or In; *n* ≥ 2).^11^ The solubility requirements of the Mg^2+^ reagent necessitate the use of THF, which inevitably precludes the formation of ligand-free heteroanthracenes. Also in the synthesis of the octafluorinated congener of DAA-Me_2_·(thf)_*n*_, where 1,2-(Me_3_Sn)_2_C_6_F_4_ and Me_2_AlCl are employed as starting materials, the cyclocondensation of the initially formed intermediate into the target product must be initiated by adding THF.^12^ Donor-free DGA-R_2_ is accessible from 1,2-(ClHg)_2_C_6_H_4_ and GaR_3_ in *p*-xylene (R = Me, Et; 140 °C, 3 h).^13^ This protocol, however, poses considerable risks due to the toxic or pyrophoric precursors and particularly the extremely harmful byproduct HgR_2_, which is released in 4 equivalents. DGA-R_2_ dimerizes *via* Ga⋯π interactions or Ga–C_b_–Ga two-electron three-center bonds (2e3c; C_b_: bridging C atom),^23^ with (DGA-Me_2_)_2_ (Fig. 1b) and (DGA-Et_2_)_2_ having distinctly different molecular structures in the solid state (see below).

Moving on to In offers novel perspectives for several reasons: (i) due to the ‘inert-pair effect’, In(i) halides are more stable and easier to handle than their Al(i) or Ga(i) counterparts. Consequently, [Hg(*o*-C_6_H_4_)]_3_ in THF can conveniently be reacted with InBr in a combined transmetallation/redox reaction to furnish DIA-Br_2_·(thf)_4_ and elemental Hg, which is a significantly less concerning byproduct compared to HgMe_2_ mentioned earlier.^14^ (ii) Due to its larger atomic radius, each In site in DIA-Br_2_ can accommodate two Lewis bases within a trigonal-bipyramidal ligand sphere, instead of just one. Given the increasing significance of coordination networks and microporous solids,^24,25^ it is noteworthy that DIA-Br_2_·(thf)_4_, when combined with rigid, ditopic Lewis bases such as 1,4-diazine, has been used to self-assemble molecular stairs and ladders.^16^ When 1,2-diazine (pyridazine, pyz) is offered to DIA-Br_2_·(thf)_4_ instead of 1,4-diazine, the system switches to a chelating mode, leading to the formation of a triptycene-type structure DIA-Br_2_·(μ-pyz)·(thf)_2_ (Fig. 1c).^17^ This outcome points to the potential application of DIA-Br_2_·(thf)_4_ as a homogeneous Lewis acid catalyst with cooperating heteroatoms.^18^

Herein, we present efficient access routes to the first donor-free 9,10-dihydro-9,10-dialuminaanthracenes (DAA-Me_2_)_2_, (1)_2_, and (DAA-Br_2_)_2_, (2)_2_, which exist as dimers in non-coordinating solvents and in the solid state. We further describe selective reactions of (1)_2_ and (2)_2_ with (i) mono- and bidentate N- or O-Lewis bases and (ii) the Lewis acid AlBr_3_.^19^ Beyond their intriguing electronic structures, these compounds hold promise as preorganized, ditopic Lewis acids^26^ and rare *ortho*-dimetallated benzene building blocks for organic synthesis.

## Results and discussion

### Syntheses

**Scheme 1:**
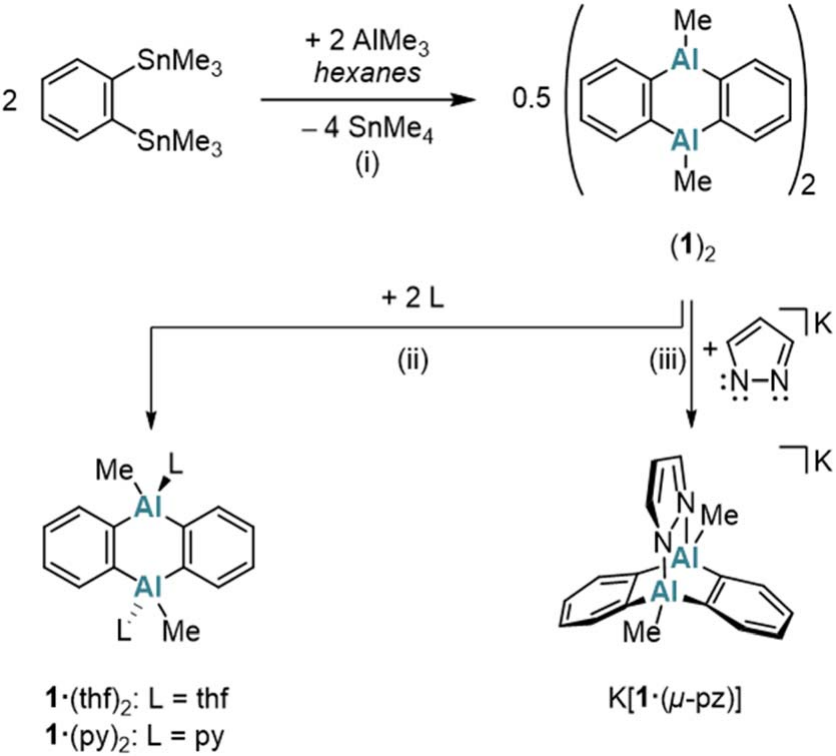
The Sn/Al exchange reaction of 1,2-(Me_3_Sn)_2_C_6_H_4_ with AlMe_3_ leads to the formation of donor-free (1)_2_. In the presence of Lewis bases (THF or py), (1)_2_ is cleaved into the monomeric diadducts 1·(thf)_2_ or 1·(py)_2_. The heterotriptycene K[1·(μ-pz)] is synthesized by reacting (1)_2_ with Kpz. (i) Hexanes, 150 °C, 3 d, sealed glass ampoule. (ii) 1·(thf)_2_: in THF, room temperature; 1·(py)_2_: 2.1 equiv. py, C_6_H_6_, room temperature. (iii) 1 equiv. Kpz, THF, room temperature.

A classical protocol for the synthesis of DBA-X_2_ is based on reactions between 1,2-(Me_3_E)_2_C_6_H_4_ and BX_3_ in toluene, *n*-hexane, or under solvent-free conditions (E = Si, Sn; X = Cl, Br).^3*a*,*d*,5^ To extend this approach to the synthesis of DAAs with the aim to avoid the use of [Hg(*o*-C_6_H_4_)]_3_ and coordinating solvents, we explored whether 1,2-(Me_3_E)_2_C_6_H_4_ could also serve as a suitable *o*-phenylene source in the present case. Eisch *et al.* reported that the reaction of the corresponding stannane with AlCl_3_ in toluene gives 1,2-(Cl_2_Al)_2_C_6_H_4_.^27^ However, a serious drawback is that the Me_3_SnCl byproduct remains firmly complexed with the aryl alane, resulting in an inseparable polymeric ion pair. Although using AlMe_2_Cl somewhat mitigated this issue – yielding a weaker electron-pair acceptor in 1,2-(Me_2_Al)_2_C_6_H_4_ – the Me_3_SnCl could still not be completely removed.^27^ Considering modified approaches, we noted that the tetrafluoro species 1,2-(Me_3_Sn)_2_C_6_F_4_ reacts with Me_2_AlCl to form dimeric [1,2-(Cl(Me)Al)_2_C_6_F_4_]_2_ and SnMe_4_ (rather than Me_3_SnCl).^28^ This suggested that starting with AlMe_3_ (ref. 19) could prevent the formation of difficult-to-remove chlorostannanes altogether. Indeed, when 1,2-(Me_3_Sn)_2_C_6_H_4_ (ref. 29) is treated with an equimolar amount of AlMe_3_ in hexanes at elevated temperatures (150 °C, 3 d, sealed glass ampoule), SnMe_4_ is released and cyclocondensation to the heteroanthracene occurs (Scheme 1). The dimeric product (1)_2_ precipitates in pure form from the reaction mixture upon cooling to room temperature (yield: 76%); (1)_2_ is highly soluble in C_6_H_6_, toluene, CHCl_3_, or CH_2_Cl_2_. The volatile byproduct SnMe_4_ can be easily removed and, in principle, subjected to a redistribution reaction with SnCl_4_ to regenerate^30^ the Me_3_SnCl required for the synthesis of the starting material 1,2-(Me_3_Sn)_2_C_6_H_4_. In the presence of Lewis-basic ligands such as tetrahydrofuran (THF) or pyridine (py), (1)_2_ is cleaved into the monomers, which are obtained as the diadducts 1·(thf)_2_ (ref. 11) and 1·(py)_2_ (Scheme 1). Of particular interest is the coordination behavior of 1 toward bidentate ligands, as this reveals the potential of 1 as a preorganized, ditopic Lewis acid. Initial exploratory investigations with pyridazine (pyz) led to the following observations: (i) the room-temperature ^1^H NMR spectrum of an equimolar mixture of (1)_2_ and pyz in THF-*d*_8_ showed only minor changes of ± 0.03 ppm compared to the chemical shift values of the signals of 1·(thf)_2_ and free pyz. (ii) Upon gas-phase diffusion of *n*-hexane into such mixtures, however, the heteroadduct 1·(pyz)(thf) crystallized, which features a pyz ligand that coordinates to one Al site through one of its N atoms, while a thf ligand coordinates to the other Al site (Fig. S41†).^16,31^ In contrast, the boron and indium congeners DBA-H_2_ and DIA-Br_2_ show triptycene-type structures with E–(μ-pyz*)_2_–E′ moieties under comparable conditions in the solid state (E = B, In; pyz* = benzo[*d*]pyridazine).^17,32^ A THF-stable heterotriptycene motif can also be imposed on 1 by using the negatively charged, five-membered pyrazolato ([pz]^−^) ligand instead of the neutral, six-membered pyz ligand (*cf.* K[1·(μ-pz)]; Scheme 1).

**Scheme 2:**
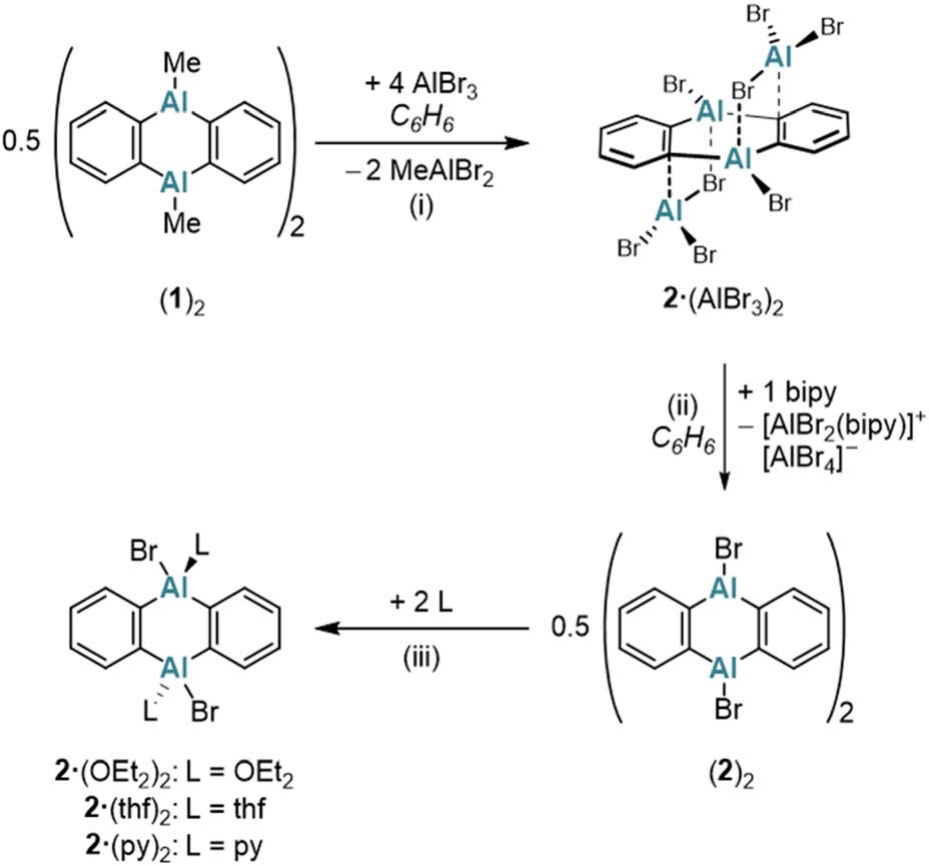
The addition of AlBr_3_ to (1)_2_ results in the immediate precipitation of 2·(AlBr_3_)_2_. Donor-free (2)_2_ is formed by the reaction of 2·(AlBr_3_)_2_ with bipy. In the presence of Lewis-bases (Et_2_O, THF, or py), (2)_2_ is cleaved into the monomeric diadducts 2·(OEt_2_)_2_, 2·(thf)_2_, or 2·(py)_2_. (i) 4 equiv. AlBr_3_ per monomeric unit 1, C_6_H_6_, room temperature, 1 d. (ii) 1 equiv. bipy, C_6_H_6_, 70 °C, 2 h, sonication. (iii) 2·(OEt_2_)_2_: exc. Et_2_O, C_6_H_6_, room temperature; 2·(thf)_2_: 2.1 equiv. THF, C_6_H_6_, room temperature; 2·(py)_2_: 2.1 equiv. py, C_6_H_6_, room temperature.

To expand the variety of donor-free DAAs, it would be desirable to obtain also an Al-halogenated derivative DAA-X_2_. One possibility is to start from (1)_2_ and to achieve the necessary Me/X exchange by the reaction with AlX_3_. AlBr_3_ was selected for this purpose because, unlike AlCl_3_, it is soluble in the non-coordinating solvent C_6_H_6_. Treatment of (1)_2_ in C_6_H_6_ with 8 equiv. AlBr_3_ leads to the instantaneous precipitation of 2·(AlBr_3_)_2_, which is generally poorly soluble in non-coordinating solvents (Scheme 2). If only 4 equiv. AlBr_3_ per (1)_2_ are used instead of 8 equiv., a structure similar to 2·(AlBr_3_)_2_ is obtained, but with the non-bridging Br positions partially occupied by Me groups [DAA-R_2_·(AlBrR_2_)_2_; R = Me or Br; according to X-ray crystallography, Fig. S42†]. Having achieved the aimed-for Me/Br exchange on 1, the next task is to remove the two coordinating AlBr_3_ molecules from 2·(AlBr_3_)_2_ to obtain the free heteroanthracene. The chelating ligand 2,2′-bipyridine (bipy) proved to be ideally suited for this purpose: In C_6_H_6_, the addition of 1 equiv. bipy to 2·(AlBr_3_)_2_ resulted in the formation of (2)_2_ after heating and sonication. NMR spectroscopy on the supernatant revealed exclusively signals of the free, dimeric (2)_2_, with no detectable bipy resonances (Scheme 2). We assume that the solid consists of species such as [AlBr_2_(bipy)][AlBr_4_], which, due to their salt-like nature, quantitatively separate from the target product.^33^ Similar to (1)_2_, (2)_2_ is converted to 2·(OEt_2_)_2_, 2·(thf)_2_, or 2·(py)_2_ upon addition of Et_2_O, THF, or py, respectively (Scheme 2).

**Scheme 3:**
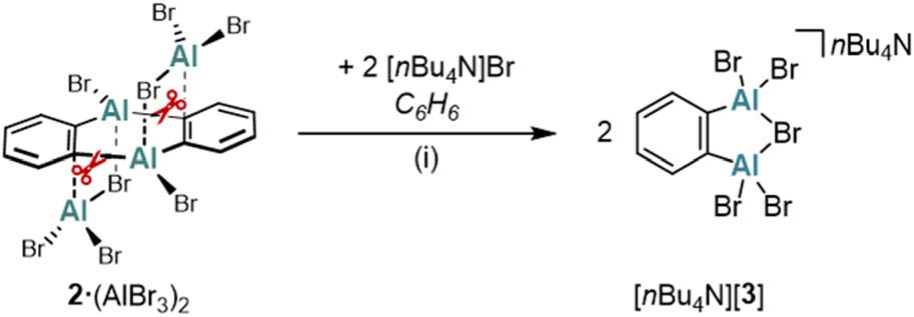
The reaction of 2·(AlBr_3_)_2_ with [*n*Bu_4_N]Br yields the 1,2-dialumino-substituted benzene derivative [*n*Bu_4_N][3]. (i) 2 equiv. [*n*Bu_4_N]Br, C_6_H_6_, 70 °C, 1.5 h, sonication.

In another attempt to generate the AlBr_3_-free (2)_2_, Br^−^ ions were used as alternative ligands instead of bipy. However, the reaction between [*n*Bu_4_N]Br (2 equiv.) and 2·(AlBr_3_)_2_ in C_6_H_6_ furnished the 1,2-dialumino-substituted benzene derivative [*n*Bu_4_N][3], rather than the initially expected products (2)_2_ and [*n*Bu_4_N][AlBr_4_] (Scheme 3). Formally, 2·(AlBr_3_)_2_ is a dimer of 1,2-(Br_2_Al)_2_C_6_H_4_, and [3]^−^ is the Br^−^ adduct of this ditopic, chelating Lewis acid (a comparable F^−^ adduct of the boron-based ditopic Lewis acid 1,2-[(C_6_F_5_)_2_B]_2_C_6_F_4_ has been characterized by NMR spectroscopy).^34^

### Solid-state structures

*Note*: Whenever we want to indicate individual DAA units in a molecular structure, we will hereafter use dashed lines for any interactions between a respective unit and the rest of the molecule. This is not intended to imply any judgements about the nature or strength of the interaction.

**Fig. 2:**
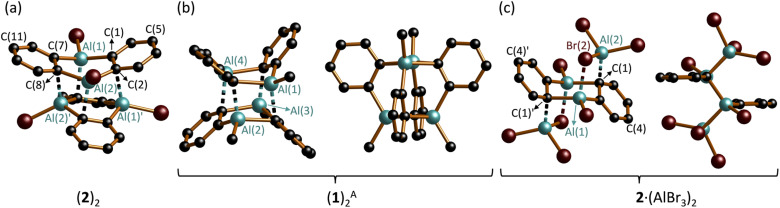
Molecular structures in the solid state: (a) (2)_2_; (b) (1)_2_^A^ viewed as a dimer of DAA monomers M, M′ (left) and as a tetramer of equivalent C_6_H_4_–Al(Me) fragments (right); (c) 2·(AlBr_3_)_2_ shown as a AlBr_3_ diadduct of DAA-Br_2_ (left) and viewed from the side (right). H atoms omitted for clarity. C: black, Br: brown, Al: turquoise.

**Fig. 3:**
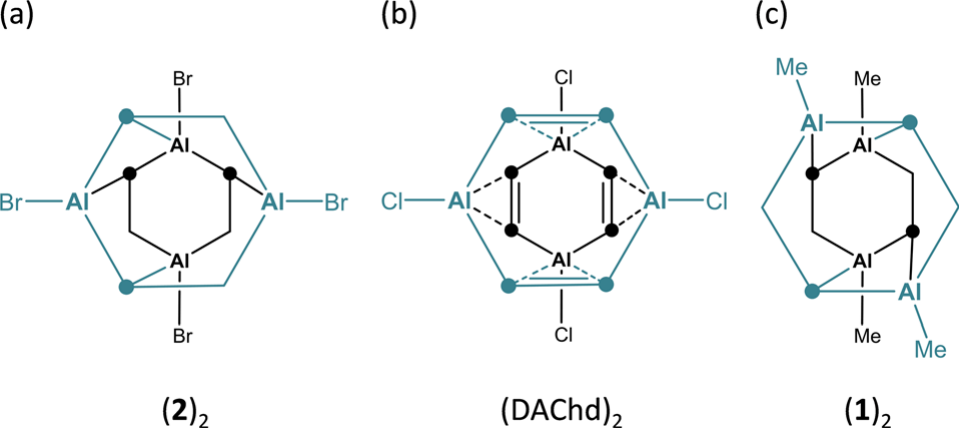
Schematic representations to illustrate the intermonomer contacts in (a) (2)_2_, (b) (DAChd)_2_, and (c) (1)_2_.

The crystal of (2)_2_ is a true racemate of discrete chiral *C*_2_-symmetric units, best described as dimers of DAA-Br_2_ molecules (Fig. 2a; note that the corresponding B-doped DBA-Br_2_ is monomeric in the solid state^3*a*^).^35^ The Al_2_C_4_ cores of the individual monomers adopt distorted, shallow boat conformations [dihedral angles Al(1)C(1)C(7)//C(1)C(2)C(7)C(8) = 35.3(2)°, Al(2)C(2)C(8)//C(1)C(2)C(7)C(8) = 6.0(2)°]. The Al atoms of monomer M (or M′) interact with two C(*ipso*) atoms, both bonded to the same Al atom of monomer M′ (or M), as schematically depicted in Fig. 3a. The corresponding bond lengths Al(1)′–C(2) and Al(2)′–C(8) measure 2.261(3) and 2.191(3) Å, respectively; the angles including these Al–C bonds and the C(2)⋯C(5) or C(8)⋯C(11) vectors across the corresponding phenylene rings are Al(1)′–C(2)⋯C(5) = 101.9(1) and Al(2)′–C(8)⋯(C11) = 111.6(2)°. In summary, (2)_2_ forms a cage structure with six-membered rings serving as the base and top, and one four-membered, two five-membered, and one six-membered ring(s) constituting the belt (Fig. S44 and S45†). To facilitate the analysis of M⋯M′ interactions in (2)_2_, we assume that each bridging C_b_ is sp^2^-hybridized, neglecting contributions from Wheland-type^36^ electronic structures with sp^3^-hybridized C_b_ atoms. Within this model, an Al′ atom from monomer M′ can engage with monomer M either through the unhybridized p_*z*_ orbital of C_b_ (Al⋯π(Ar) interaction) or *via* the Al–C_b_ σ bond (to generate a 2e3c bond). Of the four M⋯M′ interactions present in (2)_2_, two are pairwise identical. Intrinsic bond orbitals (IBOs) of the remaining two distinct interaction types are illustrated in Fig. S52.† Both types have contributions from Al⋯π(Ar) and 2e3c interactions, but to varying degrees: Based on the interpretation of IBOs, Wiberg bond indices (WBIs), and Mayer bond orders (MBOs), the two M⋯M′ interactions within the four-membered ring of the belt appear to be dominated by 2e3c bonding, whereas the other two intermonomer bonds are predominantly of the Al⋯π(Ar) type (Fig. S52 and S53†). (2)_2_ can be compared with the dimeric 1,4-dichloro-2,3,5,6-tetramethyl-1,4-**d**i**a**lumina-2,5-**c**yclo**h**exa**d**iene [(DAChd)_2_], where the two non-planar monomers are rotated by 90° relative to each other and are linked *via* four Al⋯π(olefin) bonds (Fig. 3b). The distance between two Al atoms of different monomers within the same dimer is about 3.00 Å, which was regarded as ‘relatively short’; according to *ab initio* calculations, Al⋯Al′ interactions contribute to the stability of the system.^37^ Indeed, the dimers remained intact under mass spectrometry conditions up to temperatures of 140 °C.^38^ In (2)_2_, the Al⋯Al′ distances range from 2.717(2) Å (across the four-membered ring) to 3.635(2) Å (across the six-membered ring). A third dimeric structural motif comparable to (2)_2_ and (DAChd)_2_ is observed in (DGA-Me_2_)_2_ (Fig. 1b): the primary distinction among these three cases lies in the degree of rotation of the monomer units relative to each other.^13^

(1)_2_ crystallizes with three crystallographically independent molecules in the unit cell, each displaying approximate *D*_2d_ symmetry [(1)_2_^A^–(1)_2_^C^].^39^ Since their key structural parameters are very similar (Fig. S35†), only (1)_2_^A^ will be discussed in detail. Although its molecular formula corresponds to a dimer of DAA-Me_2_, the assignment of two distinct DAA units within the total ensemble is less unambiguous than in the case of (2)_2_, owing to the higher symmetry of (1)_2_^A^. For the purposes of the discussion to follow, the six-membered rings containing Al(1)/Al(4) and Al(2)/Al(3) are defined as belonging to monomers M and M′ (Fig. 2b, left; an alternative definition is possible but leads to the same conclusions). Both monomeric units exhibit twist-boat conformations. Unlike (2)_2_, the Al atoms of monomer M (or M′) in (1)_2_^A^ interact with two diagonally opposite C(*ipso*) atoms attached to different Al atoms of monomer M′ (or M) (*cf.*Fig. 3c). The respective ‘intermonomeric’ Al–C bond lengths range from 2.100(2) to 2.132(2) Å, which are shorter than those of (2)_2_. While these findings are informative for the comparison of the solid-state structures of (1)_2_ and (2)_2_, an alternative analysis of the (1)_2_ scaffold is more appropriate to account for its high symmetry: the cluster comprises four equivalent C_6_H_4_–Al(Me) fragments, each featuring a C(Ar)–Al σ bond; the second deprotonated *o*-phenylene C atom bridges two additional Al atoms, forming an Al′–C_b_–Al′′ 2e3c bond (Fig. 2b, right). Consequently, each Al vertex is tetracoordinated by C atoms. The Al_4_ core of (1)_2_ adopts a strongly distorted tetrahedral geometry with Al⋯Al distances of av. 2.666 and av. 3.611 Å. Overall, the framework of (1)_2_ resembles that of (DGA-Et_2_)_2_ (ref. 13) and of the *o*-phenylene magnesium tetramer [Mg(thf)(*o*-C_6_H_4_)]_4_, with the Al atoms replaced by Mg atoms and the Me substituents by thf ligands (Fig. S36 and S37†).^21^ To conclude the discussion of structures (1)_2_ and (2)_2_, we find it remarkable that both species prefer to form discrete dimers in a cluster-like arrangement rather than coordination polymers with Al–(μ-Me/Br)_2_–Al′ bridges, as seen in Al_2_Me_6_ (ref. 40) and Al_2_Br_6_;^41^ the diboraanthracene (DBA-H_2_)_∞_ is indeed polymeric *via* B–(μ-H)_2_–B′ linkages in the solid state.^42^ Furthermore, it is worth noting the following result from quantum chemical calculations (SMD(C_6_H_6_)/ωB97XD/def2-TZVPP//SMD(C_6_H_6_)/ωB97XD/def2-TZVPP): after Me/Br or Br/Me exchange, the resulting (1^Br^)_2_ or (2^Me^)_2_ remain minima on the potential-energy surface. For R = Me, the crystallographically observed structure (1)_2_ is more stable than (2^Me^)_2_ by 4.9 kcal mol^−1^. Yet, for R = Br, structure (2)_2_ is less stable than (1^Br^)_2_ by 3.5 kcal mol^−1^, in the absence of crystal-packing effects.^43^

In the *C*_i_-symmetric compound 2·(AlBr_3_)_2_, two AlBr_3_ moieties coordinate to opposite sides of the DAA-Br_2_ core, which consequently adopts a chair conformation (Fig. 2c).^44^ Similar to (1)_2_, the binding sites are two diagonally opposite C(*ipso*) atoms (*cf.*Fig. 3c). The respective bonds are relatively short (Al(2)–C(1) = 2.032(4) Å), and the C(1) atoms are strongly pyramidalized (Al(2)–C(1)⋯C(4) = 134.0(2)°, Al(1)–C(1)′⋯C(4)′ = 124.0(2)°). Each Al(2)–C(1) bond is reinforced by a Br atom that bridges the Al atoms of DAA-Br_2_ and AlBr_3_ (Al(1)–Br(2) = 2.449(1) Å, Al(2)–Br(2) = 2.411(1) Å). In summary, the largely symmetric Al(1)⋯Al(1)′-bridging mode of the phenylene ring in 2·(AlBr_3_)_2_ more closely resembles the situation in (1)_2_^A^ than in (2)_2_. Compound 2·(AlBr_3_)_2_ is formally the dimer of 1,2-(Br_2_Al)_2_C_6_H_4_. The related [1,2-(Cl(Me)Al)_2_C_6_F_4_]_2_, which was characterized by Gabbaï *et al.* with X-ray diffraction, has a markedly different molecular structure: It features two stacked 1,2-phenylene rings, two distinct types of Al⋯Al′-bridging Cl^−^ ions, and lacks any Al–C_b_–Al 2e3c bonds.^28^

**Fig. 4:**
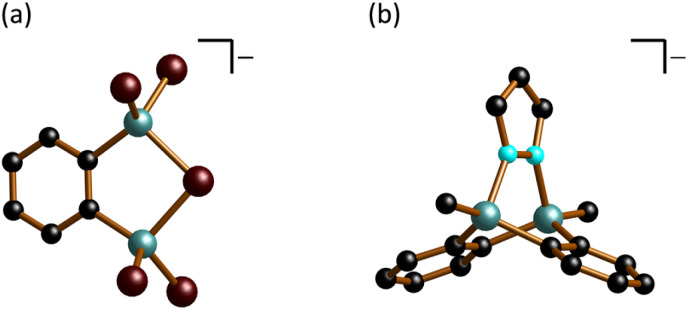
Molecular structures in the solid state: (a) [*n*Bu_4_N][3]; (b) [K(thf)_1.5_][1·(μ-pz)]. Counter cations and H atoms omitted for clarity. C: black, Br: brown, Al: turquoise, N: cyan.

The compound [*n*Bu_4_N][3] is asymmetric in the solid state, although the anionic component approximates the *C*_2v_ point group (Fig. 4a). [3]^−^ can be described as a ditopic Lewis acid (*i.e.*, 1,2-(Br_2_Al)_2_C_6_H_4_), where the two vicinally positioned Al sites cooperate in bonding to the same Br^−^ anion, with an average bond length of Al–(μ-Br) = 2.443 Å. As expected, these bonds are longer than the Al–Br bonds to the terminal Br atoms, which range from 2.282(2) to 2.294(2) Å. When the bridging Br^−^ ion is excluded from consideration, the sum of angles within the remaining CAlBr_2_ fragments averages 343.7°, which lies between the typical values of a planar (360°) and a tetrahedral geometry (328.5°). The endocyclic angles (μ-Br)–Al–C and Al–(μ-Br)–Al are av. 101.8° and 90.7(1)°, respectively.

In the crystal lattice, [K(thf)_1.5_][1·(μ-pz)] forms a one-dimensional coordination polymer, with [K(thf)]^+^ and [K(thf)_2_]^+^ cations bonding simultaneously to phenylene rings of two different anions (Fig. S40†). The anion [1·(μ-pz)]^−^ represents a rare heterotriptycene with Al atoms at the bridgehead positions (Fig. 4b).^45–47^ The Al–N bonds to the bridging pyrazolato ([pz]^−^) ring (av. 1.973 Å) are longer than the Al–N(pz) bonds in [R_2_Al–(μ-pz)_2_–AlR_2_] (R = Me: av. 1.921 Å, *t*Bu: av. 1.929 Å)^48^ or in bicyclic [HAl(μ-3,5-*t*Bu_2_pz)_2_(μ-CH_2_N*t*Bu)AlH] (av. 1.914 Å).^45^

The monomeric complexes 1·(py)_2_ × C_6_H_6_ and 2·(py)_2_ × C_6_H_6_ are isostructural in the crystalline state and show planar, *C*_i_-symmetric DAA-R_2_ units coordinated by two py ligands from opposite sides (*trans* configuration; R = Br, Me). Also, the thf diadducts 1·(thf)_2_ and 2·(thf)_2_ adopt *trans* configurations. Remarkably, the OEt_2_ ligands in the corresponding diadduct 2·(OEt_2_)_2_ are positioned in a *cis* arrangement in the solid state (full details are given in the ESI†).

### NMR analysis

For all molecules presented in this work, the ^27^Al NMR resonances were broadened beyond detection. NMR spectroscopic analysis was not possible for 2·(AlBr_3_)_2_ and [*n*Bu_4_N][3], due to their low solubility in all suitable solvents. In the cases of the Et_2_O/thf/py diadducts of 1 and 2 as well as K[1·(μ-pz)] and (1)_2_, the number of ^1^H and ^13^C NMR signals, their chemical shift values, and the integral ratios of the proton resonances were consistent with the (symmetry-averaged) molecular structures determined by X-ray analysis (full details are given in the ESI†). (2)_2_ is the only example that requires a more thorough consideration: Its solid-state structure has insufficient symmetry to align with the ^1^H and ^13^C NMR spectra obtained in solution (C_6_D_6_), which show only two and three signals, respectively. There are two possible explanations for the observed NMR features: (i) (2)_2_ may dissociate in solution into the monomeric units 2. (ii) The dimeric structure may persist but experience significant fluctuations due to librational motion of the monomers relative to each other, resulting in an average *D*_2d_ symmetry. A quantum-chemical analysis renders the conversion of (2)_2_ → 2 × 2 unlikely, as it would be endergonic with Δ*G* = 28.0 kcal mol^−1^ (which is in line with the shortened Al⋯Al′ distances discussed in the crystallographic section). In contrast, any activation barrier to be overcome during the librational motion does not exceed Δ*G*^‡^ = 3.0 kcal mol^−1^ (Scheme S1†), which makes option (ii) more probable.

### Potential of [*n*Bu_4_N][3] as synthesis equivalent of the 1,2-dideprotonated benzene nucleophile

The arylaluminum species described here, while interesting in their own right, also hold potential as *ortho*-dimetallated starting materials for organic synthesis. Such nucleophilic building blocks, which complement their ubiquitous, polarity inverted *o*-dihalogenated analogues, are just as valuable as they are difficult to access:^49^ A major challenge is to avoid the unwanted formation of benzyne on the way to 1,2-M_2_C_6_H_4_ (M = Li, MgBr). The key starting material for most *o*-dimetallated benzenes therefore still remains *o*-phenylene mercury, [Hg(*o*-C_6_H_4_)]_3_, which is obtained from 1,2-Br_2_C_6_H_4_ and sodium amalgam in a process that takes several days.^50,51^ [Hg(*o*-C_6_H_4_)]_3_ can subsequently be converted into the organolithium, -magnesium, or -zinc species 1,2-Li_2_C_6_H_4_, [Mg(thf)(*o*-C_6_H_4_)]_4_, or [Zn(thf)_2_(*o*-C_6_H_4_)]_*n*_ [*n* = 2 (crystalline state) or 3 (solution)] by reaction with metallic Li,^50,52^ Mg,^21^ or Zn,^53^ respectively; the required reaction times range from half a day to weeks. Taken together, the widespread use of *o*-dimetallated benzenes is hindered not only by the well-recognized environmental and health concerns associated with organomercury compounds but also by the apparently poor reproducibility in the synthesis of [Hg(*o*-C_6_H_4_)]_3_: while Wittig claimed to have obtained yields of 50%, Massey explicitly stated that, despite ‘10 years of experience in the field’, they consistently observed yields as low as 1–2%.^50,51^ Even though, in favorable cases, the *in situ* generation of nucleophilic intermediates from 1,2-Br_2_C_6_H_4_ and Mg can be achieved in the presence of the electrophile, resulting in satisfactory yields (as demonstrated in the synthesis of 1,2-(Me_3_Sn)_2_C_6_H_4_),^29^ there is a persistent demand for efficient access to additional *o*-dimetallated benzenes.

**Scheme 4:**
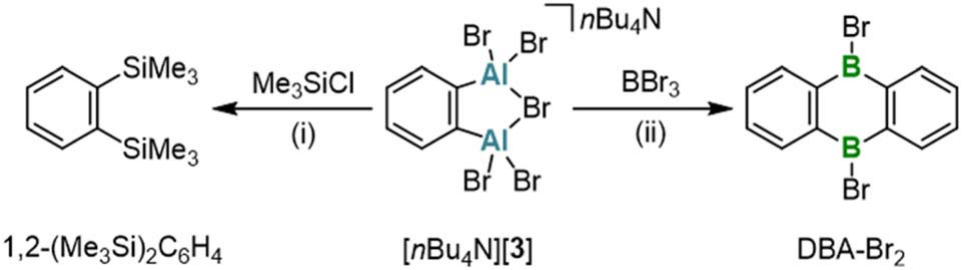
Synthesis of 1,2-(Me_3_Si)_2_C_6_H_4_ or DBA-Br_2_ by the reaction of [*n*Bu_4_N][3] and Me_3_SiCl or BBr_3_, respectively. (i) 2 equiv. Me_3_SiCl, C_6_D_6_, room temperature, 1 d. (ii) Excess BBr_3_, room temperature.

Recognizing the potential of our aryl aluminium compound [*n*Bu_4_N][3] as an ideal candidate to address this need, we conducted several proof-of-concept experiments. For this purpose, we selected target compounds with published synthesis protocols that require prolonged reaction times and/or high temperatures (Scheme 4). This allows us to identify potential advantages of our new starting material through direct comparison: For instance, the reaction between [*n*Bu_4_N][3] and 2 equiv. Me_3_SiCl in C_6_D_6_ furnished the disubstituted benzene 1,2-(Me_3_Si)_2_C_6_H_4_ at room temperature after 1 d (quantitative conversion according to NMR spectroscopy; Fig. S25†). In contrast, the established synthesis of the same product from 1,2-Br_2_C_6_H_4_, Mg, and Me_3_SiCl *via* a Grignard-type reaction in THF requires stirring for 2 d, with the temperature gradually increasing from 0 °C to room temperature.^54^ Particularly noteworthy is the conversion of [*n*Bu_4_N][3] with neat BBr_3_, which instantaneously affords the corresponding 9,10-dihydro-9,10-diboraanthracene already at room temperature (Scheme 4). The traditional route to DBA-Br_2_*via* 1,2-(Me_3_Si)_2_C_6_H_4_ and BBr_3_ requires heating to 120 °C for 6 d.^3*a*,55^

Already this selection of straightforward conversions highlights the potential of [3]^−^ as a synthetic equivalent of 1,2-dideprotonated benzene. Despite its poor solubility in non-polar solvents like C_6_H_6_, it still undergoes smooth heterogeneous reactions. The products, however, are nicely soluble in C_6_H_6_, allowing for easy separation from any unreacted starting material and Al-containing byproduct salts, which greatly simplifies the purification process.

## Conclusions

Anthracenes incorporating Group 13 elements at the 9,10-positions have significant potential as redox-active systems and versatile ditopic Lewis acids, offering a broad range of applications. Their planar structures expose the reactive heteroelement sites, while the rigid 1,2-phenylene bridges bring these sites into close proximity, promoting acid–base binding cooperativity. Additionally, the delocalized π systems enable electronic communication between the dopant atoms. While 9,10-dihydro-9,10-diboraanthracenes have been extensively studied and higher homologues have received some attention, 9,10-dihydro-9,10-dialuminaanthracenes (DAA-R_2_; R: terminal Al-bonded substituent) have remained almost entirely unexplored, despite Al being by far the most earth-abundant Group 13 element.

In this work, we synthesized DAA-Me_2_ (1) in the absence of stabilizing ligands, resulting in its dimerization through Al⋯π(Ar) interactions to form (1)_2_. Despite dimerization, (1)_2_ serves as an effective synthesis equivalent for ‘free’ DAA-Me_2_, as it readily reacts with mono- or bidentate Lewis bases to afford *trans*-diadducts such as 1·(py)_2_, or triptycene-type structures like K[1·(μ-pz)] (py: pyridine; Hpz: pyrazole). Notably, (1)_2_ can be cleaved into its monomers not only by Lewis bases but also by the strong Lewis acid AlBr_3_, which displaces the original DAA-Me_2_ partner by establishing new Br_3_Al⋯π(Ar) bonds and Al–Br–Al′ bridges in its place. This reaction also exchanges all Al-bonded Me substituents of DAA-Me_2_ for Br atoms, leading to 2·(AlBr_3_)_2_, the double AlBr_3_ adduct of DAA-Br_2_ (2), which can also be viewed as a dimer of 1,2-(Br_2_Al)_2_C_6_H_4_. The donor-free (2)_2_ can be liberated from 2·(AlBr_3_)_2_ using 2,2′-bipyridine. In contrast, treatment of 2·(AlBr_3_)_2_ with Br^−^ ions splits two Al–C bonds to give the adduct [*n*Bu_4_N][3], in which one molecule of 1,2-(Br_2_Al)_2_C_6_H_4_ chelates one Br^−^ ion. The anion [3]^−^ has proven to be an excellent synthon for a 1,2-dideprotonated benzene. Such compounds are rare but of exceptional synthetic value; the broader utility of [3]^−^ in this regard is currently under investigation in our laboratories.

## Data availability

The datasets supporting this article have been uploaded as part of the ESI.†

## Author contributions

P. L. L. performed the experimental studies and characterized all new compounds. P. L. L. and J. G. performed the quantum-chemical calculations. A. V. performed the X-ray crystal structure analyses of all compounds. H.-W. L. and M. W. supervised the project. The manuscript was written by P. L. L. and M. W. and edited by all co-authors.

## Conflicts of interest

There are no conflicts to declare.

## Supplementary Material

SC-OLF-D4SC06940D-s001

SC-OLF-D4SC06940D-s002

SC-OLF-D4SC06940D-s003

## References

[cit1] Borissov A., Maurya Y. K., Moshniaha L., Wong W.-S., Żyła-Karwowska M., Stępień M. (2022). Chem. Rev..

[cit2] Prey S. E., Wagner M. (2021). Adv. Synth. Catal..

[cit3] Januszewski E., Lorbach A., Grewal R., Bolte M., Bats J. W., Lerner H.-W., Wagner M. (2011). Chem.–Eur. J..

[cit4] Kessler S. N., Wegner H. A. (2010). Org. Lett..

[cit5] Brend'amour S., Gilmer J., Bolte M., Lerner H.-W., Wagner M. (2018). Chem.–Eur. J..

[cit6] Jin T., Bolte M., Lerner H.-W., Wagner M. (2022). Org. Chem. Front..

[cit7] Kirschner S., Mewes J.-M., Bolte M., Lerner H.-W., Dreuw A., Wagner M. (2017). Chem.–Eur. J..

[cit8] Kaehler T., Bolte M., Lerner H.-W., Wagner M. (2019). Angew. Chem., Int. Ed..

[cit9] Melaimi M., Gabbaï F. P. (2005). Adv. Organomet. Chem..

[cit10] Oishi M. , Product Subclass 9: Triorganoaluminum Compounds, in Science of Synthesis, ed. H. Yamamoto, Georg Thieme Verlag KG, Stuttgart, 2004, pp. 261–385

[cit11] Dam M. A., Nijbacker T., de Kanter F. J. J., Akkerman O. S., Bickelhaupt F., Spek A. L. (1999). Organometallics.

[cit12] Tschinkl M., Cocker T. M., Bachman R. E., Taylor R. E., Gabbaï F. P. (2000). J. Organomet. Chem..

[cit13] Jutzi P., Sielemann H., Neumann B., Stammler H.-G. (2005). Inorg. Chim. Acta.

[cit14] Gabbaï F. P., Schier A., Riede J., Schichl D. (1996). Organometallics.

[cit15] Dam M. A., Nijbacker T., de Pater B. C., de Kanter F. J. J., Akkerman O. S., Bickelhaupt F., Smeets W. J. J., Spek A. L. (1997). Organometallics.

[cit16] Gabbaï F. P., Schier A., Riede J. (1998). Angew. Chem., Int. Ed..

[cit17] Gabbaï F. P., Schier A., Riede J., Hynes M. J. (1998). Chem. Commun..

[cit18] Tschinkl M., Schier A., Riede J., Gabbaï F. P. (1998). Inorg. Chem..

[cit19] We are aware that the compounds AlMe_3_, AlBr_3_, and MeAlCl_2_ are monomeric neither in solution nor in the solid state. However, for simplicity, the monomeric forms were used in calculating the quantities employed.

[cit20] Woodard C. M., Hughes G., Massey A. G. (1976). J. Organomet. Chem..

[cit21] Tinga M. A. G. M., Akkerman O. S., Bickelhaupt F., Horn E., Spek A. L. (1991). J. Am. Chem. Soc..

[cit22] This study focuses exclusively on heteroanthracenes that lack substituents on the phenylene rings. Substituents attached to Al/Ga/In are denoted after the hyphen; for example, DAA-Me_2_ refers to a 9,10-dihydro-9,10-dialuminaanthracene with methyl substituents at the Al sites. The type and number of coordinated Lewis-basic ligands (if present) are added in parentheses.

[cit23] Hübner A., Diefenbach M., Bolte M., Lerner H.-W., Holthausen M. C., Wagner M. (2012). Angew. Chem., Int. Ed..

[cit24] Janiak C. (1997). Angew. Chem., Int. Ed..

[cit25] Zheng X., Kato M., Uemura Y., Matsumura D., Yagi I., Takahashi K., Noro S., Nakamura T. (2023). Inorg. Chem..

[cit26] Timoshkin A. Y. (2024). Chem.–Eur. J..

[cit27] Eisch J. J., Mackenzie K., Windisch H., Krüger C. (1999). Eur. J. Inorg. Chem..

[cit28] Tschinkl M., Bachman R. E., Gabbaï F. P. (1999). Chem. Commun..

[cit29] Eisch J. J., Kotowicz B. W. (1998). Eur. J. Inorg. Chem..

[cit30] Scott W. J., Crisp G. T., Stille J. K. (1990). Org. Synth..

[cit31] Fontani M., Peters F., Scherer W., Wachter W., Wagner M., Zanello P. (1998). Eur. J. Inorg. Chem..

[cit32] Lorbach A., Bolte M., Lerner H.-W., Wagner M. (2010). Chem. Commun..

[cit33] (i) The addition of monodentate pyridine (2 equiv.) also releases (2)_2_. However, the AlBr_3_/py adduct(s) that are formed as byproduct(s) remain soluble and are therefore difficult to remove. (ii) Adding 2 equiv. bipy to 2·(AlBr_3_)_2_ results in the precipitation of all Al-containing compounds (in C_6_D_6_); the ^1^H NMR spectrum of the supernatant shows no resonances, except for the solvent signal. (iii) As control experiments, we have also prepared 2:1 and 1:1 mixtures of AlBr_3_ and bipy in C_6_D_6_. In both cases, a precipitate formed; the supernatants showed either no bipy signals (2:1 mixture) or significant bipy signals (1:1 mixture). NMR analysis of each precipitate in CD_3_CN displayed a strong resonance at *δ*(^27^Al) = 81.0 ppm, assignable to the [AlBr_4_]^−^ anion (literature value: 80.0 ppm as taken from: J. W. Akitt, Aluminum, Gallium, Indium, and Thallium, in *Multinuclear NMR*, ed. J. Mason, Plenum Press, New York, 1987, ch. 9, p. 269. The ^1^H resonance patterns of both precipitates were essentially identical, though rather complex, indicating the presence of various bipy-containing cations. Overall, the NMR characteristics of the precipitate that had been isolated (in quantitative yield) during the synthesis of (2)_2_ are comparable to those observed in the control experiments. For an earlier study of the AlBr_3_/bipy system, see: J. Y. Corey and R. Lamberg, *Inorg. Nucl. Chem. Lett.*, 1972, **8**, 275–280

[cit34] Williams V. C., Piers W. E., Clegg W., Elsegood M. R. J., Collins S., Marder T. B. (1999). J. Am. Chem. Soc..

[cit35] Deposition Numbers 2385628 (for (1)_2_), 2385629 (for 1·(py)_2_), 2385630 (for K[1·(μ-pz)]), 2385631 (for 1·(pyz)(thf)), 2385632 (for DAA-R_2_·(AlBrR_2_)_2_ (R = Me or Br)), 2385633 (for α-2·(AlBr_3_)_2_), 2385634 (for β-2·(AlBr_3_)_2_), 2385635 (for (2)_2_), 2385636 (for 2·(thf)_2_), 2385637 (for 2·(py)_2_), 2385638 (for [*n*Bu_4_N][3]), 2385639 (for 1·(thf)_2_), and 2385640 (for 2·(OEt_2_)_2_) contain the ESI† crystallographic data for this paper. These data are provided free of charge by the joint Cambridge Crystallographic Data Centre and Fachinformationszentrum Karlsruhe Access Structures service.

[cit36] Reed C. A., Kim K.-C., Stoyanov E. S., Stasko D., Tham F. S., Mueller L. J., Boyd P. D. W. (2003). J. Am. Chem. Soc..

[cit37] Schnöckel H., Leimkühler M., Lotz R., Mattes R. (1986). Angew. Chem., Int. Ed..

[cit38] Hoberg H., Gotor V., Milchereit A., Krüger C., Sekutowski J. C. (1977). Angew. Chem., Int. Ed..

[cit39] To facilitate the structure discussion, we will from now on treat (1)_2_^A^ as if it had an exact *D*_2d_ symmetry in the solid state.

[cit40] Vranka R. G., Amma E. L. (1967). J. Am. Chem. Soc..

[cit41] Eley D. D., Taylor J. H., Wallwork S. C. (1961). J. Chem. Soc..

[cit42] Lorbach A., Bolte M., Li H., Lerner H.-W., Holthausen M. C., Jäkle F., Wagner M. (2009). Angew. Chem., Int. Ed..

[cit43] Nangia A. , Molecular Conformation and Crystal Lattice Energy Factors in Conformational Polymorphs, in Models, Mysteries and Magic of Molecules, ed. J. C. A. Boeyens and J. F. Ogilvie, Springer, Dordrecht, 2008, ch. 3, pp. 63–86

[cit44] The compound 2·(AlBr_3_)_2_ crystallizes in two polymorphous modifications that differ by the number of crystallographically unique molecules (two molecules in the denser α-form and one in the less dense β-form). Since the geometrical characteristics of the molecules are similar, we are focusing here on the β-form. More details are provided in the ESI.†

[cit45] Zheng W., Stasch A., Prust J., Roesky H.-W., Cimpoesu F., Noltemeyer M., Schmidt H.-G. (2001). Angew. Chem., Int. Ed..

[cit46] Zheng W., Mösch-Zanetti N. C., Roesky H.-W., Noltemeyer M., Hewitt M., Schmidt H.-G., Schneider T. R. (2000). Angew. Chem., Int. Ed..

[cit47] Seven Ö., Popp S., Bolte M., Lerner H.-W., Wagner M. (2014). Dalton Trans..

[cit48] Chang C.-C., Her T.-Y., Hsieh F.-Y., Yang C.-Y., Chiang M. Y., Lee G.-H., Wang Y., Peng S.-M. (1994). J. Chin. Chem. Soc..

[cit49] Bickelhaupt F. (1987). Angew. Chem., Int. Ed..

[cit50] Wittig G., Bickelhaupt F. (1958). Chem. Ber..

[cit51] Al-Jabar N. A. A., Massey A. G. (1984). J. Organomet. Chem..

[cit52] Winkler H. J. S., Wittig G. (1963). J. Org. Chem..

[cit53] Goedheijt M. S., Nijbacker T., Akkerman O. S., Bickelhaupt F., Veldman N., Spek A. L. (1996). Angew. Chem., Int. Ed..

[cit54] Lorbach A., Reus C., Bolte M., Lerner H.-W., Wagner M. (2010). Adv. Synth. Catal..

[cit55] The reaction of [*n*Bu_4_N][3] with Me_2_SiCl_2_ or Me_3_SnCl gives 9,9,10,10-tetramethyl-9,10-dihydro-9,10-disilaanthracene or 1,2-(Me_2_BrSn)_2_C_6_H_4_, respectively. These reactions are not yet optimized and represent the onset of an investigation into the full scope of the reaction, which will be reported at a later stage.

