# Supplementary material for: Donor-free 9,10-dihydro-9,10-dialuminaanthracenes
Source: Chem Sci. 2024 Nov 18;16(1):147–55. doi: 10.1039/d4sc06940d (PMC11609976; doi:10.1039/d4sc06940d)
Supplement: SC-016-D4SC06940D-s002 [file SC-016-D4SC06940D-s002.pdf]

Supplementary Information (SI) for Chemical Science.
This journal is © The Royal Society of Chemistry 2024

Supporting Information

Donor-free 9,10-dihydro-9,10-dialuminaanthracenes

Paula L. Lückert, Jannik Gilmer, Alexander Virovets, Hans-Wolfram Lerner, and Matthias Wagner*

Institut für Anorganische und Analytische Chemie, Goethe-Universität Frankfurt, Max-von-Laue-Straße 7, D-60438 Frankfurt (Main), Germany

*To whom correspondence should be addressed. Email: matthias.wagner@chemie.uni-frankfurt.de

Table of Contents

1 Experimental details and characterization data S3

1.1 Synthesis of (**1**)$_2$ S4

1.2 Synthesis of **1**·(thf)$_2$ S5

1.3 Synthesis of **1**·(py)$_2$ S6

1.4 Synthesis of K[**1**·(μ-pz)] S7

1.5 Synthesis of **1**·(pyz)(thf) S7

1.6 Synthesis of DAA-R$_2$·(AlBrR$_2$)$_2$ (R = Me or Br) S8

1.7 Synthesis of **2**·(AlBr$_3$)$_2$ S8

1.8 Synthesis of (**2**)$_2$ S9

1.9 Synthesis of **2**·(OEt$_2$)$_2$ S9

1.10 Synthesis of **2**·(thf)$_2$ S10

1.11 Synthesis of **2**·(py)$_2$ S11

1.12 Synthesis of [*n*Bu$_4$N][**3**] S11

1.12.1 Reactivity of [*n*Bu$_4$N][**3**] toward Me$_3$SiCl S12

1.12.2 Reactivity of [*n*Bu$_4$N][**3**] toward BBr$_3$ S12

1.13 Modified synthesis of 1,2-bis(trimethylsilyl)benzene S13

2 Plots of NMR spectra S14

3 Single-crystal X-ray structure analyses S31

3.1 Single-crystal X-ray structure analysis of (**1**)$_2$ S39

3.2 Single-crystal X-ray structure analysis of **1**·(thf)$_2$ S41

3.3 Single-crystal X-ray structure analysis of **1**·(py)$_2$ × C_6H_6 .... S41

3.4 Single-crystal X-ray structure analysis of [K(thf)$_{1.5}$]$_2$[**1**·(μ-pz)]$_2$ S42

3.5 Single-crystal X-ray structure analysis of **1**·(pyz)(thf) S43

3.6 Single-crystal X-ray structure analysis of DAA-R$_2$·(AlBrR$_2$)$_2$ (R = Me or Br) S44

3.7 Single-crystal X-ray structure analyses of two polymorphous modifications of **2**·(AlBr$_3$)$_2$ S45

3.8 Single-crystal X-ray structure analysis of (**2**)$_2$ S47

3.9 Single-crystal X-ray structure analysis of **2**·(OEt$_2$)$_2$ S48

3.10 Single-crystal X-ray structure analysis of **2**·(thf)$_2$ S48

3.11 Single-crystal X-ray structure analysis of **2**·(py)$_2$ × C_6H_6 .... S49

3.12 Single-crystal X-ray structure analysis of [*n*Bu$_4$N][**3**] S49

4 X-ray powder diffraction S50

4.1 X-ray powder diffractometry on **2**·(AlBr$_3$)$_2$ S50

5 Computational details S51

5.1 Rearrangement processes in the dimer (**2**)$_2$ S52

5.2 Characterization of the bonding situation in (**2**)$_2$ S53

5.3 Computed structures and free energies S55

6 References S57

1 Experimental details and characterization data

General considerations. All reactions, manipulations, and analyses of air- and moisture-sensitive compounds were carried out under an atmosphere of dry argon or nitrogen using Schlenk techniques or in an argon- or nitrogen-filled glovebox. *n*-Pentane and *n*-hexane were dried over Na metal; Et_2O, THF, C_6H_6, and toluene were dried over Na/benzophenone. Prior to use, the solvents were degassed by applying three freeze-pump-thaw cycles and stored over molecular sieves (3 Å). C_6D_6 and THF-d_8 were dried over Na-K alloy without benzophenone (1 d), degassed as described above, and stored over molecular sieves (3 Å). CD_2Cl_2 was stored over molecular sieves (3 Å). Pyridine (py) and pyridazine (pyz) were stored over molecular sieves (3 Å). Me_3SiCl and Me_2SiCl_2 were distilled from CaH_2. BBr_3 was stored over Hg to remove traces of Br_2. All other commercially available reagents were used as received. 1,2-Bis(trimethylstannyl)benzene[S1] and potassium pyrazolide (Kpz)[S2] were synthesized according to literature procedures.

If not stated otherwise, NMR spectra were recorded at 298 K using the following Bruker spectrometers: *Avance™ II 300*, *Avance™ III 400 HD, Avance™ III 500 HD*, or *Avance™ DRX 600.* Chemical shift values are referenced to (residual) solvent signals ($^1H/^{13}C\{^1H\}$; C_6D_6: δ = 7.16/128.06 ppm; CD_2Cl_2: δ = 5.32/53.84 ppm; THF-d_8: δ = 3.58/67.21), external $Al(NO_3)_3$ (0.00 ppm), external $SiMe_4$ (0.00 ppm), or external $BF_3{\cdot}Et_2O$ (0.00 ppm).[S3] Abbreviations: s = singlet, t = triplet, q = quartet, m = multiplet, br = broad, n.o. = not observed, n.r. = not resolved. Resonances of carbon atoms attached to aluminum atoms were typically broadened and sometimes only observed in ^1H-^{13}C-HMBC NMR experiments due to the quadrupolar relaxation of the ^{27}Al nucleus. Resonance assignments were aided by ^1H-^1H-COSY, ^1H-^{13}C-HSQC, ^1H-^{13}C-HMBC, ^1H-^{13}C-H2BC-NMR, and ^1H-^{29}Si-HMBC experiments.

High-resolution mass spectra were measured in negative-ion mode using *Bruker Apollo II MTP timsTOFfleX* (ESI, MALDI); *trans*-2-[3-(4-*t*-butylphenyl)-2-methyl-2-propenylidene]malononitrile served as the matrix.

Elemental analyses (%CHN) were performed on a HEKAtech *EURO FA* 3000 Elemental Analyzer.

1.1 Synthesis of (1)$_2$

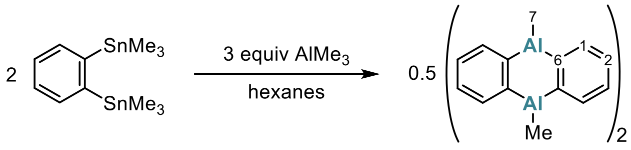

1,2-(Me$_3$Sn)$_2$C$_6$H$_4$ (**1**)$_2$

Inside a nitrogen-filled glovebox, a thick-walled glass ampoule was charged with 1,2-(Me$_3$Sn)$_2$C$_6$H$_4$ (1.13 g, 2.80 mmol) and *n*-hexane (1 mL). AlMe$_3$[S4] (2.0 M in hexanes; 2.1 mL, 4.2 mmol) was added at room temperature via syringe. The ampoule was flame-sealed at –196 °C under vacuum and subsequently heated to 150 °C in an oven for 3 d. The ampoule was allowed to cool to room temperature, whereupon (**1**)$_2$ precipitated as a colorless solid. The ampoule was opened in the glovebox and the liquid phase was removed with a syringe. The remaining colorless solid was dried at room temperature for 3 h in a dynamic vacuum. Subsequently, the solid was dissolved in C$_6$H$_6$ (5 mL), filtered through a 0.45 µm PTFE syringe filter to remove any insoluble residues, and freeze-dried to obtain (**1**)$_2$. Yield of (**1**)$_2$: 251 mg (531 µmol, 76 %).

Notes: a) A stoichiometric ratio (1:1) of 1,2-(Me$_3$Sn)$_2$C$_6$H$_4$ and AlMe$_3$ can also be used; however, for convenience, excess AlMe$_3$ was employed in the ampoule reaction. Unconsumed AlMe$_3$ can be easily removed, as the final product (**2**)$_2$ precipitates at the end of the reaction. b) NMR spectroscopic investigations indicate that the reaction is complete after about 1 d at 150 °C. Nevertheless, we typically store the ampoule in the oven for a more extended period to ensure full conversion.

Single crystals of (**1**)$_2$ suitable for X-ray diffraction were grown in an argon-filled glovebox by slow evaporation of a solution of (**1**)$_2$ in *n*-hexane (Figure S34).

^{1}H NMR (500.2 MHz, C$_6$D$_6$): δ = 8.02–7.98 (m, 4H; H-1), 6.98–6.94 (m, 4H; H-2), 0.01 (s, 6H; H-7).

^{13}C{^{1}H} NMR (125.8 MHz, C$_6$D$_6$): δ = 160.3 (br; C-6), 145.0 (C-1), 129.8 (C-2), –7.7 (br; C-7).

^{27}Al NMR (130.3 MHz, C$_6$D$_6$): δ = n.o.

^{1}H NMR (500.2 MHz, CD$_2$Cl$_2$): δ = 8.04–8.01 (m, 4H; H-1), 7.35–7.32 (m, 4H; H-2), –0.24 (s, 6H; H-7).

^{13}C{^{1}H} NMR (125.8 MHz, CD$_2$Cl$_2$): δ = 160.4 (br; C-6), 145.1 (C-1), 129.9 (C-2), –8.1 (br; C-7).

^{27}Al NMR (130.3 MHz, CD$_2$Cl$_2$): δ = n.o.

EA (%): Calculated for the monomer C$_{14}$H$_{14}$Al$_2$ [236.23]: C 71.18, H 5.97; found: C 70.07, H 5.90.

1.2 Synthesis of 1·(thf)$_2$

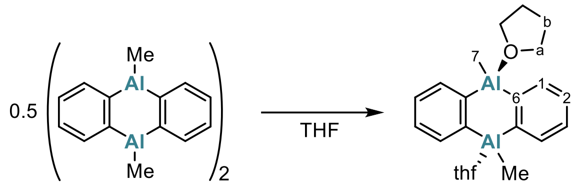

($\mathbf{1}$)$_2$ 1·(thf)$_2$

Inside an argon-filled glovebox, a round-bottom flask was charged at room temperature with (**1**)$_2$ (36 mg, 76 µmol) and THF (0.5 mL). The reaction mixture was stirred for 5 min at room temperature inside the glovebox. All volatiles were removed from the reaction mixture under reduced pressure to obtain **1**·(thf)$_2$ as a colorless solid. Yield of **1**·(thf)$_2$: 57 mg (0.15 mmol, quantitative).

The ^{1}H and ^{13}C NMR shift values measured for this sample are consistent with the literature data.[S5]

Single crystals of **1**·(thf)$_2$ suitable for X-ray diffraction were grown in an argon-filled glovebox by slow evaporation of a solution of **1**·(thf)$_2$ in THF and *n*-hexane (Figure S38).

^{1}H NMR (500.2 MHz, C_6D_6): δ = 8.09–8.06 (m, 4H; H-1), 7.56–7.52 (m, 4H; H-2), 3.39–3.36 (m, 8H; H-a), 0.77–0.74 (m, 8H; H-b), 0.07 (s, 6H; H-7).

^{13}C{^{1}H} NMR (125.8 MHz, C_6D_6): δ = 162.8 (br; C-6), 136.7 (C-1), 126.5 (C-2), 71.0 (C-a), 24.6 (C-b), –12.1 (br; C-7).

^{27}Al NMR (130.3 MHz, C_6D_6): δ = n.o.

^{1}H NMR (600.2 MHz, THF-*d*$_8$): δ = 7.61–7.58 (m, 4H; H-1), 7.05–7.02 (m, 4H; H-2), 3.64–3.59 (m, 6H*; H-a), 1.80–1.74 (m, 6H*; H-b), –0.51 (s, 6H; H-7). *) The actual integral values are somewhat smaller (Figure S7), because, only in this case, the sample remained under a dynamic vacuum overnight, which obviously removed some of the coordinated thf.

^{13}C{^{1}H} NMR (150.9 MHz, THF-*d*$_8$): δ = 162.7 (br; C-6), 136.4 (C-1), 125.9 (C-2), 68.0 (C-a), 26.1 (C-b), –13.0 (br; C-7).

^{27}Al NMR (130.3 MHz, THF-*d*$_8$): δ = n.o.

1.3 Synthesis of 1·(py)$_2$

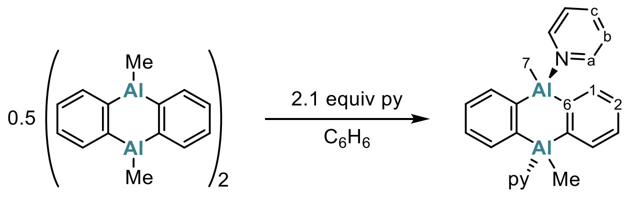

Inside an argon-filled glovebox, an NMR tube was charged at room temperature with (**1**)$_2$ (35 mg, 74 µmol), C_6H_6 (0.5 mL), and pyridine (py; 25.1 µL, 0.31 mmol), whereupon **1**·(py)$_2$ precipitated as a colorless solid, which was dried in a dynamic vacuum. Yield of **1**·(py)$_2$: 57 mg (0.14 mmol, quantitative).

Single crystals of **1**·(py)$_2$ × C_6H_6 suitable for X-ray diffraction were grown in an argon-filled glovebox through gas-phase diffusion of C_6H_6 into a THF-d_8 solution of **1**·(py)$_2$ (Figure S39).

^{1}H NMR (500.2 MHz, THF-d_8): δ = 8.40–8.38 (m, 4H; H-a), 7.76–7.73 (m, 2H; H-c), 7.68–7.65 (m, 4H; H-1), 7.28–7.25 (m, 4H; H-b), 7.08–7.05 (m, 4H; H-2), –0.43 (s, 6H; H-7).

^{13}C{^{1}H} NMR (125.8 MHz, THF-d_8): δ = 163.6 (br; C-6), 148.9 (C-a), 140.2 (C-c), 136.7 (C-1), 126.0 (C-2), 125.6 (C-b), –11.9 (br; C-7).

^{27}Al NMR (130.3 MHz, THF-d_8): δ = n.o.

1.4 Synthesis of K[1·(*μ*-pz)]

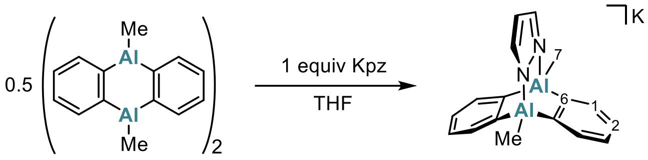

(**1**)$_2$ K[**1**·(*μ*-pz)]

Inside an argon-filled glovebox, a round bottom flask was charged at room temperature with (**1**)$_2$ (29 mg, 61 µmol), THF (0.5 mL), and potassium pyrazolide (Kpz; 13 mg, 0.12 mmol). The reaction mixture was stirred for 5 min at room temperature inside the glovebox. The solution was filtered through a 0.45 µm PTFE syringe filter. All volatiles were removed under reduced pressure, whereupon [K(thf)][**1**·(*μ*-pz)] remained as a colorless solid. Yield of [K(thf)][**1**·(*μ*-pz)]: 48 mg (0.12 mmol, 94 %).

Single crystals of [K(thf)$_{1.5}$][**1**·(*μ*-pz)] suitable for X-ray diffraction were grown in an argon-filled glovebox by slow evaporation of a solution of [K(thf)][**1**·(*μ*-pz)] in a mixture of THF and *n*-pentane (Figure S40). *Note*: To determine the yield, we use the amount of K^+-coordinated thf indicated by the ^{1}H NMR spectrum ([K(thf)]$^+$); if we refer to the crystal structure, we use the amount of thf indicated by X-ray diffraction ([K(thf)$_{1.5}$]$^+$).

^{1}H NMR (600.2 MHz, THF-*d*$_8$): δ = 7.65 (n.r.; 2H; H-a), 7.58–7.57 (m, 4H; H-1), 6.88–6.86 (m, 4H; H-2), 6.11 (n.r., 1H; H-b), 3.63–3.61 (m, 4H; H^{thf}), 1.79–1.76 (m, 4H; H^{thf}), –0.26 (s, 6H; H-7).

^{13}C{^{1}H} NMR (150.9 MHz, THF-*d*$_8$): δ = 168.0 (br; C-6), 137.8 (C-a), 134.9 (C-1),124.5 (C-2), 104.1 (C-b), 68.0 (C^{thf}), 26.2 (C^{thf}), –16.3 (br; C-7).

^{27}Al NMR (130.3 MHz, THF-*d*$_8$): δ = n.o.

HRMS (ESI): *m/z* calculated for [$C_{17}H_{17}Al_2N_2$]$^-$: 303.1028 [M]$^-$; found: 303.0945.

1.5 Synthesis of 1·(pyz)(thf)

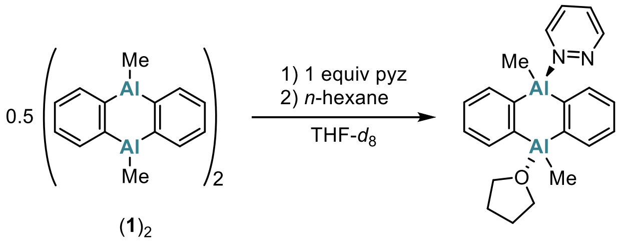

(**1**)$_2$ **1**·(pyz)(thf)

In an argon-filled glovebox, pyridazine (pyz; 3.1 µL, 42 µmol) was added to (**1**)$_2$ (10 mg, 21 µmol) in THF-*d*$_8$ (0.5 mL). The NMR tube was flame-sealed at –196 °C under vacuum, and NMR spectra were recorded (Figure S13). After the addition of a second equivalent of pyz, NMR spectra were recorded again (Figure S13).

Single crystals of **1**·(pyz)(thf) suitable for X-ray diffraction were grown in an argon-filled glovebox through gas-phase diffusion of *n*-hexane into the THF-*d*$_8$ solution (Figure S41).

1.6 Synthesis of DAA-R_2·$(AlBrR_2)_2$ (R = Me or Br)

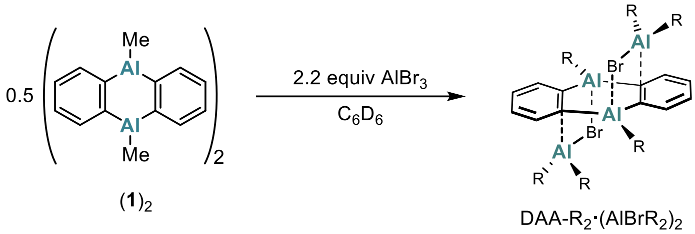

In a nitrogen-filled glovebox, an NMR tube was charged at room temperature with (**1**)$_2$ (12 mg, 25 µmol), C_6D_6 (0.5 mL), and $AlBr_3$[S4] (30 mg, 0.11 mmol), whereupon a colorless suspension was formed. The reaction mixture was filtered through a 0.45 µm PTFE syringe filter and the filtrate was then analyzed by NMR-spectroscopy.

Single crystals of DAA-R_2·$(AlBrR_2)_2$ suitable for X-ray diffraction were grown in an argon-filled glovebox through gas-phase diffusion of *n*-hexane into a C_6D_6 solution of DAA-R_2·$(AlBrR_2)_2$ (R = Me or Br; Figure S42).

Note: Some of the NMR signals of DAA-R_2·$(AlBrR_2)_2$ are severely broadened at room temperature and are thus not very diagnostic (Figure S14); up to a measurement temperature of –30 °C, no significant changes are observable. Since these resonances cannot be straightforwardly assigned to the proposed structure of DAA-R_2·$(AlBrR_2)_2$, our structural characterization rests mainly on X-ray crystallography.

1.7 Synthesis of 2·$(AlBr_3)_2$

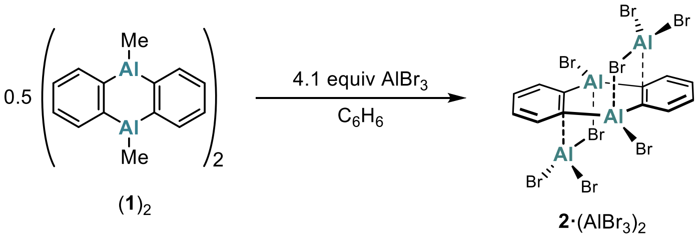

Inside a nitrogen-filled glovebox, $AlBr_3$[S4] (1.12 g, 4.18 mmol) was added at room temperature to a stirred solution of (**1**)$_2$ (241 mg, 510 µmol) in C_6H_6 (3 mL), whereupon a colorless suspension formed. After the suspension had been stirred overnight, the clear supernatant was removed via syringe. The remaining colorless solid was suspended in *n*-hexane (3 mL) and the suspension was stirred for 5 min. The clear supernatant was removed again, and the procedure was repeated with 2 mL of *n*-hexane. The byproduct Me_nAlBr_{3-n}[S4] and the unconsumed $AlBr_3$ were removed from the remaining crude product by sublimation (150 °C, 10^{-3} Torr, 3 h). **2**·$(AlBr_3)_2$ was finally obtained as a colorless solid. Yield of **2**·$(AlBr_3)_2$: 546 mg (607 µmol, 60 %).

Single crystals of two polymorphs of **2**·$(AlBr_3)_2$ suitable for X-ray diffraction were grown in an argon-filled glovebox by a) layering a solution of (**1**)$_2$ (15 mg, 32 µmol) in C_6D_6 (0.5 mL) with C_6H_6 (0.2 mL) and $AlBr_3$ (91 mg, 0.34 mmol) in *n*-hexane (0.5 mL; Figure S43) or b) slow evaporation of a filtered solution of **2**·$(AlBr_3)_2$ in C_6D_6 and *n*-hexane (Figure S43).

Note: Since **2**·$(AlBr_3)_2$ is poorly soluble in non-polar solvents and not stable in polar solvents, an NMR spectroscopic analysis was not possible (Figure S15). The bulk purity of **2**·$(AlBr_3)_2$ was therefore proven by X-ray powder diffraction (Figure S50).

1.8 Synthesis of (2)$_2$

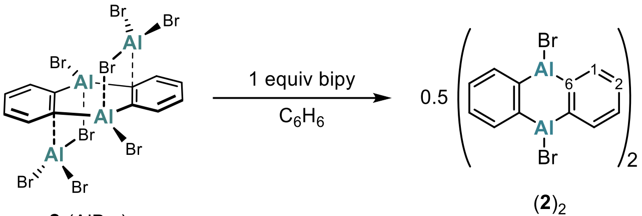

Inside an argon-filled glovebox, a Schlenk tube was charged with **2**·$(AlBr_3)_2$ (100 mg, 111 µmol), C_6H_6 (2.5 mL), and 2,2'-bipyridine (bipy; 17 mg, 109 µmol) at room temperature inside an argon-filled glovebox, whereupon a yellow solid precipitated. The reaction mixture was sonicated for 2 h at 70 °C. The suspension was filtered through a 0.45 µm PTFE syringe filter. The remaining solution was freeze-dried to afford (**2**)$_2$ as a colorless powder. Yield of (**2**)$_2$: 34 mg (93 µmol, 84 %).

Single crystals of (**2**)$_2$ suitable for X-ray diffraction were grown in an argon-filled glovebox by slow evaporation of a solution of (**2**)$_2$ in C_6H_6 and *n*-hexane (Figure S44).

^{1}H NMR (500.2 MHz, C_6D_6): δ = 8.49–8.45 (m, 4H; H-1), 6.82–6.79 (m, 4H; H-2).

^{13}C{^{1}H} NMR (125.8 MHz, C_6D_6): δ = 153.3 (br; C-6), 148.3 (C-1), 132.3 (C-2).

^{27}Al NMR (130.3 MHz, C_6D_6): δ = n.o.

1.9 Synthesis of 2·(OEt$_2$)$_2$

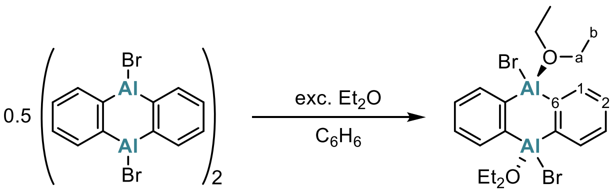

Inside an argon-filled glovebox, a round-bottom flask was charged at room temperature with (**2**)$_2$ (10 mg, 14 µmol), C_6H_6 (0.5 mL), and Et_2O (0.1 mL, 1 mmol). The reaction mixture was stirred for 5 min at room temperature inside the glovebox. All volatiles were removed from the reaction mixture under reduced pressure to obtain **2**·$(OEt_2)_2$ as a colorless solid (Figure S18). The obtained product was crystallized through gas-phase diffusion of *n*-hexane into a C_6H_6 solution (0.5 mL) of **2**·$(OEt_2)_2$. Colorless crystals of **2**·$(OEt_2)_2$ were isolated (Figure S46). Yield of **2**·$(OEt_2)_2$: 4 mg (8 µmol, 28 %).

^{1}H NMR (600.2 MHz, C_6D_6): δ = 8.15–8.12 (m, 4H; H-1), 7.46–7.43 (m, 4H; H-2), 3.45 (q, $^3J_{H,H}$ = 7.0 Hz, 8H; H-a), 0.53 (t, $^3J_{H,H}$ = 7.0 Hz, 12H; H-b).

^{13}C{^{1}H} NMR (150.9 MHz, C_6D_6): δ = 157.9* (br; C-6), 136.9 (C-1), 127.9** (C-2), 68.2 (C-a), 13.2 (C-b). *) This signal was only detected in the ^{1}H-^{13}C-HMBC NMR experiment. **) This signal was only detected in the ^{1}H-^{13}C-HMBC NMR experiment, because it overlaps with the signal of C_6D_6.

^{27}Al NMR (130.3 MHz, C_6D_6): δ = n.o.

1.10 Synthesis of 2·(thf)$_2$

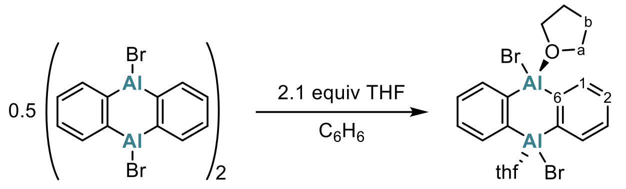

Inside an argon-filled glovebox, a round-bottom flask was charged at room temperature with (**2**)$_2$ (10 mg, 14 µmol), C_6H_6 (0.5 mL), and THF (4.7 µL, 57 µmol). The reaction mixture was stirred for 5 min at room temperature inside the glovebox. All volatiles were removed from the reaction mixture under reduced pressure. The colorless residue was dissolved in C_6D_6 (0.5 mL). NMR spectroscopic investigation revealed the formation of **2**·(thf)$_2$ (Figures S21, S22).

Single crystals of **2**·(thf)$_2$ suitable for X-ray diffraction were grown in an argon-filled glovebox through gas-phase diffusion of *n*-hexane into a THF-d_8 solution of the yellow precipitate obtained during the synthesis of (**2**)$_2$ (Figure S47).

^{1}H NMR (500.2 MHz, C_6D_6): δ = 8.21–8.17 (m, 4H; H-1), 7.50–7.47 (m, 4H; H-2), 3.55-3.52 (m, 8H*; H-a), 1.00–0.97 (m, 8H*; H-b). *) For reasons that are not yet understood, the measured integral values are approximately twice as high as theoretically expected (this situation doesn't change when the sample is stored under a dynamic vacuum for extended periods of time). Additionally, the reaction exhibits lower selectivity compared to that furnishing **2**·(OEt$_2$)$_2$ (cf. paragraph 1.9 above). We have included **2**·(thf)$_2$ in this study with the sole purpose to compare its solid-state structure with that of **2**·(OEt$_2$)$_2$.

^{13}C{^{1}H} NMR (125.8 MHz, C_6D_6): δ = 157.7 (br; C-6), 137.0 (C-1), 127.9** (C-2), 71.2 (C-a), 25.0 (C-b). **) This signal was only detected in the ^{1}H-^{13}C-HMBC NMR experiment, because it overlaps with the signal of C_6D_6.

^{27}Al NMR (130.3 MHz, C_6D_6): δ = n.o.

1.11 Synthesis of 2·(py)$_2$

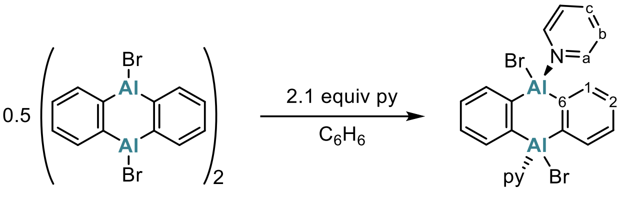

Inside an argon-filled glovebox, an NMR tube was charged at room temperature with (**2**)$_2$ (10 mg, 14 µmol), C_6H_6 (0.5 mL), and pyridine (py; 4.6 µL, 57 µmol), whereupon **2**·(py)$_2$ precipitated as an off-white solid. The clear, yellow supernatant was removed via syringe. The remaining solid was washed with *n*-hexane (3 × 0.3 mL) and dried in vacuo to obtain **2**·(py)$_2$ as an off-white solid. Yield of **2**·(py)$_2$: 14 mg (27 µmol, quantitative).

Single crystals of **2**·(py)$_2$ × C_6H_6 suitable for X-ray diffraction were grown in an argon-filled glovebox by layering a solution of **2**·($AlBr_3$)$_2$ (10 mg, 11 µmol) in C_6H_6 (0.5 mL) with C_6H_6 (0.2 mL) and py (0.1 mL, 1 mmol) in *n*-hexane (0.5 mL) (Figure S48). *Note*: The same unit cell was found when py was added to (**2**)$_2$, in which case crystals also formed.

^{1}H NMR (500.2 MHz, THF-*d*$_8$): δ = 8.63–8.61 (m, 4H; H-a), 7.89–7.86 (m, 2H; H-c), 7.74–7.71 (m, 4H; H-1), 7.45–7.42 (m, 4H; H-b), 7.16–7.12 (m, 4H; H-2).

^{13}C{^{1}H} NMR (125.8 MHz, THF-*d*$_8$): δ = 158.6 (br; C-6), 149.7 (C-a), 139.4 (C-c), 136.8 (C-1), 127.2 (C-2), 125.4 (C-b).

^{27}Al NMR (130.3 MHz, THF-*d*$_8$): δ = n.o.

1.12 Synthesis of [*n*Bu$_4$N][3]

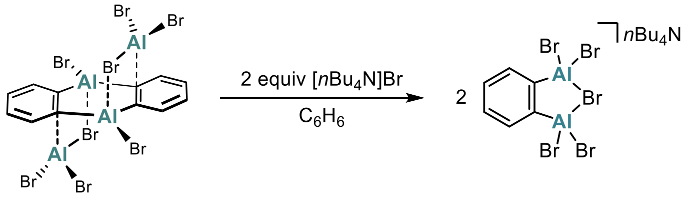

A Schlenk tube was charged with **2**·($AlBr_3$)$_2$ (374 mg, 416 µmol), C_6H_6 (3 mL), and [*n*Bu$_4$N]Br (268 mg, 832 µmol). The suspension was sonicated at 70 °C for 1.5 h until no more solids were visible. The biphasic mixture obtained was placed in the fridge (4 °C, 1 h), whereupon [*n*Bu$_4$N][**3**] precipitated as a colorless solid. The supernatant was removed and the solid was washed with *n*-hexane (2 × 1 mL) to obtain [*n*Bu$_4$N][**3**] as a colorless solid. Yield of [*n*Bu$_4$N][**3**]: 635 mg (822 µmol, quantitative).

Single crystals of [*n*Bu$_4$N][**3**] suitable for X-ray diffraction were grown in an argon-filled glovebox through gas-phase diffusion of *n*-hexane into a C_6D_6 solution of [*n*Bu$_4$N][**3**] (Figure S49).

Note: Since [*n*Bu$_4$N][**3**] is poorly soluble in non-polar solvents and not stable in polar solvents, an NMR spectroscopic analysis was not possible.

HRMS (MALDI): *m/z* calculated for [$C_6H_4Al_2Br_5$]$^-$: 524.5866 [M]$^-$; found: 524.5883.

EA (%): Calculated for $C_{22}H_{40}Al_2Br_5N$ [772.05]: C 34.23, H 5.22, N 1.81; found: C 32.99, H 5.23, N 1.88.

1.12.1 Reactivity of [nBu$_4$N][3] toward Me_3SiCl

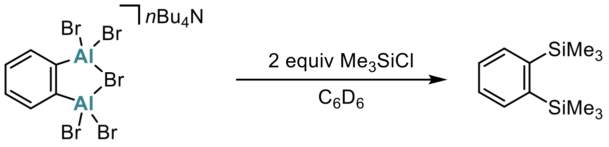

[nBu$_4$N][**3**] 1,2-($Me_3Si)_2C_6H_4$

Inside a nitrogen-filled glovebox, Me_3SiCl (3.3 µL, 26 µmol) was added to [nBu$_4$N][**3**] (10 mg, 13 µmol) in C_6D_6 (0.5 mL). The NMR tube was flame-sealed at –196 °C under vacuum and stored for 1 d at room temperature. NMR spectra were recorded (Figures S25, S26, S27).

The observed ^{1}H, ^{13}C, and ^{29}Si NMR shift values were consistent with the values of an authentic sample of 1,2-($Me_3Si)_2C_6H_4$, synthesized via a modified published protocol (cf. paragraph 1.13 below).

1.12.2 Reactivity of [nBu$_4$N][3] toward BBr_3

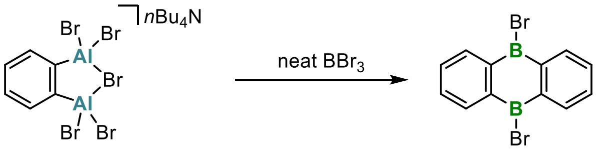

[nBu$_4$N][**3**] DBA-Br_2

Inside a nitrogen-filled glovebox, an NMR tube was filled with [nBu$_4$N][**3**] (50 mg, 65 µmol). BBr_3 (0.5 mL) was added. The NMR tube was flame-sealed at –196 °C under vacuum and stored at room temperature for 1 d. The NMR tube was opened in the glovebox and the reaction mixture was filtered through a 0.45 µm PTFE syringe filter. Impurities were removed from the remaining crude product by sublimation (80 °C, 10^{-3} Torr, 2 h), yielding DBA-Br_2 as a pale yellow solid (Figures S28, S29, S30).

The ^{1}H, ^{13}C, and ^{11}B NMR shift values measured for this sample are consistent with the literature data.[S6]

1.13 Modified synthesis of 1,2-bis(trimethylsilyl)benzene

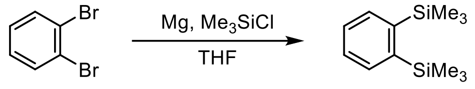

1,2-$Br_2C_6H_4$ 1,2-$(Me_3Si)_2C_6H_4$

1,2-$(Me_3Si)_2C_6H_4$ was prepared according to a published protocol with slight modifications.[S7]

Mg turnings (4.0 g, 0.17 mmol) were placed in a Schlenk flask equipped with a dropping funnel and heated under vacuum with a Bunsen burner while stirring. After cooling to room temperature, the Mg turnings were covered with THF (60 mL) and Me_3SiCl (70 mL, 0.55 mol). A solution of 1,2-dibromobenzene (5.0 mL, 42 mmol) in toluene (20 mL) was added dropwise at 0 °C. The reaction mixture was stirred for 3 d, during which time it was allowed to warm to room temperature. The reaction mixture was carefully added to a mixture of a saturated aqueous solution of $NaHCO_3$ (200 mL) and *n*-hexane (100 mL). Two phases formed, which were separated by using a separation funnel. The aqueous phase was extracted with *n*-hexane (3 × 40 mL), the combined organic phases were dried over $MgSO_4$, and filtered. All volatiles were removed from the filtrate under reduced pressure. 1,2-$(Me_3Si)_2C_6H_4$ was isolated as a colorless liquid by fractional distillation using a Vigreux column (50–55 °C; 10^{-3} Torr). Yield of 1,2-$(Me_3Si)_2C_6H_4$: 7.2 g (32 mmol, 76 %).

^{1}H NMR (500.2 MHz, C_6D_6): δ = 7.70–7.66 (m, 2H; H-Ar), 7.22–7.19 (m, 2H; H-Ar), 0.35 (s, 18H; H-Me).

^{13}C{^{1}H} NMR (125.8 MHz, C_6D_6): δ = 146.0 (C-Ar), 135.7 (C-Ar), 128.2 (C-Ar), 2.1 (C-Me).

^{29}Si{^{1}H} NMR (99.4 MHz, C_6D_6): δ = –3.9 (s).

The ^{1}H, ^{13}C, and ^{29}Si NMR shift values measured for this sample are consistent with the literature data.[S8]

2 Plots of NMR spectra

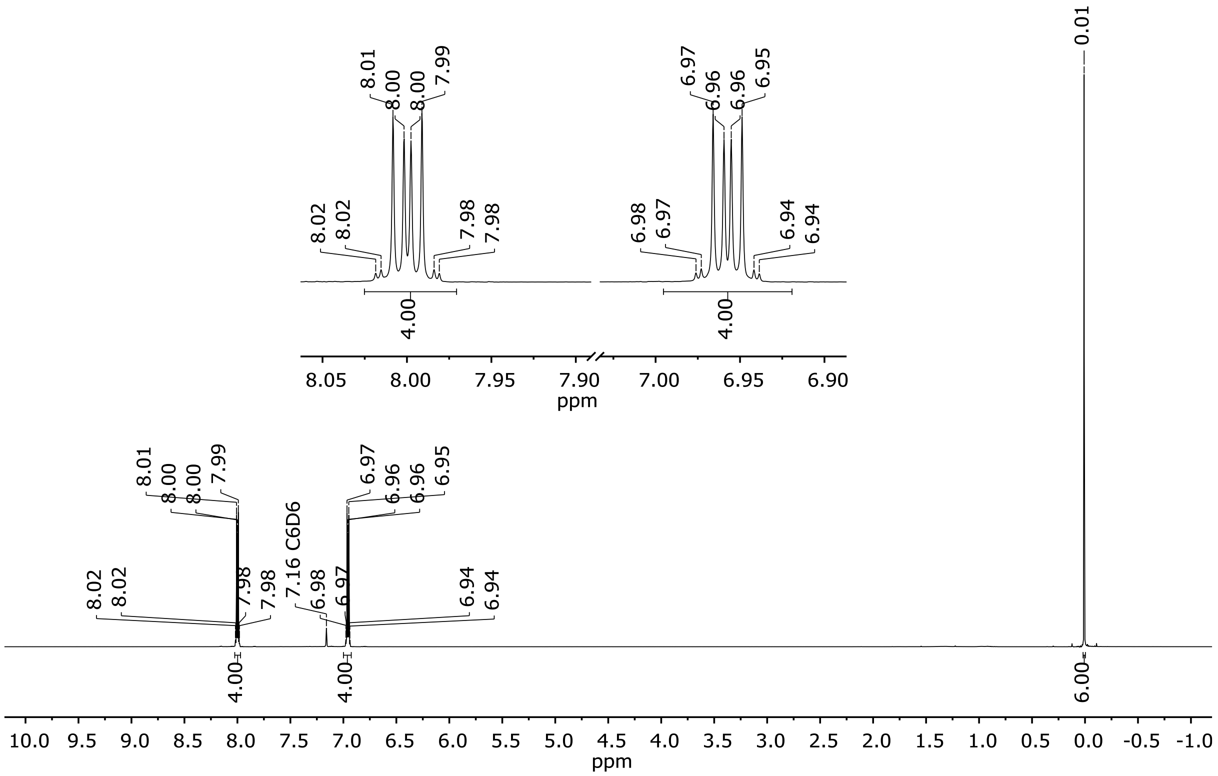

Figure S1: ^{1}H NMR spectrum of (**1**)$_2$ (500.2 MHz, C_6D_6).

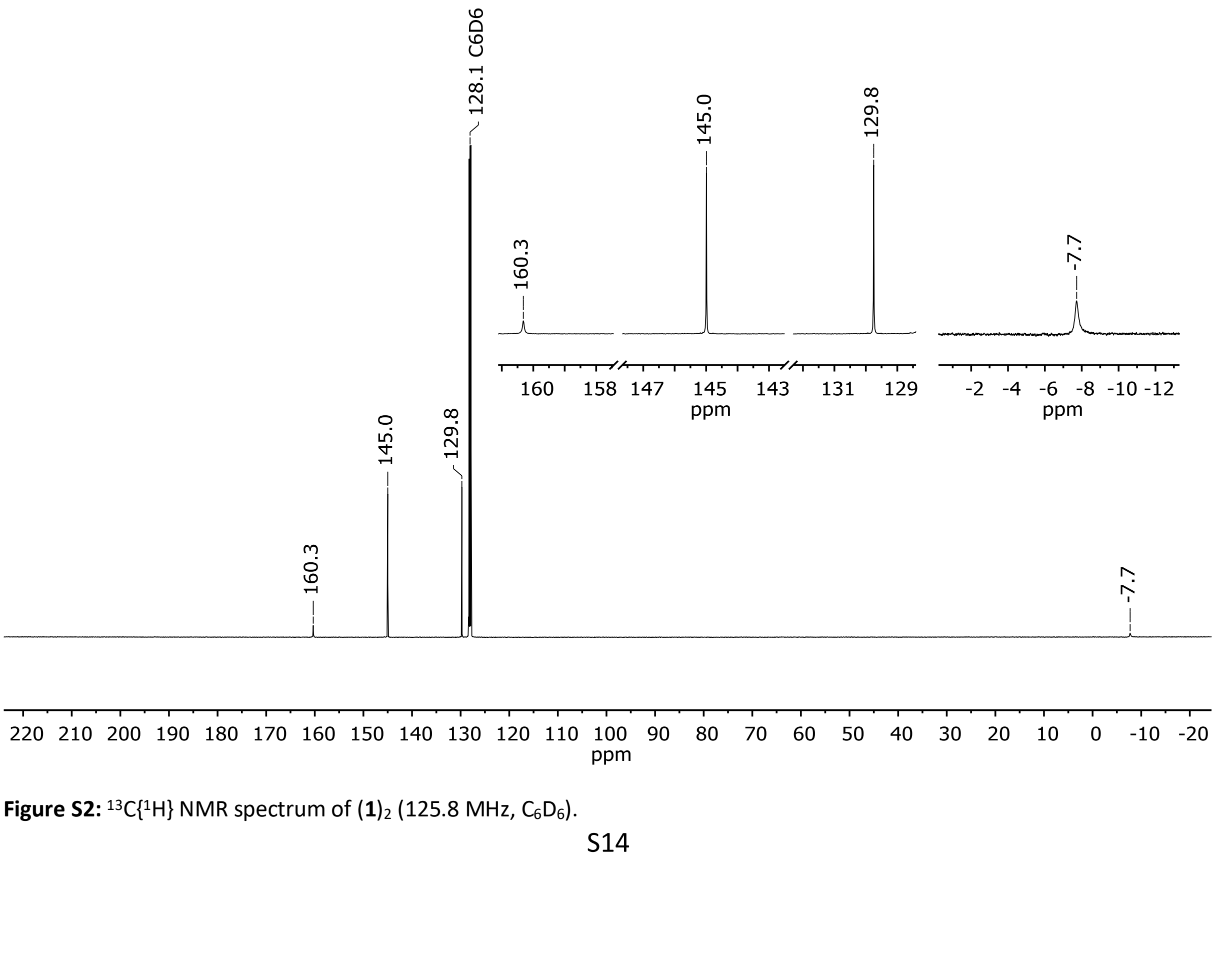

Figure S2: $^{13}C\{^{1}H\}$ NMR spectrum of (**1**)$_2$ (125.8 MHz, C_6D_6).

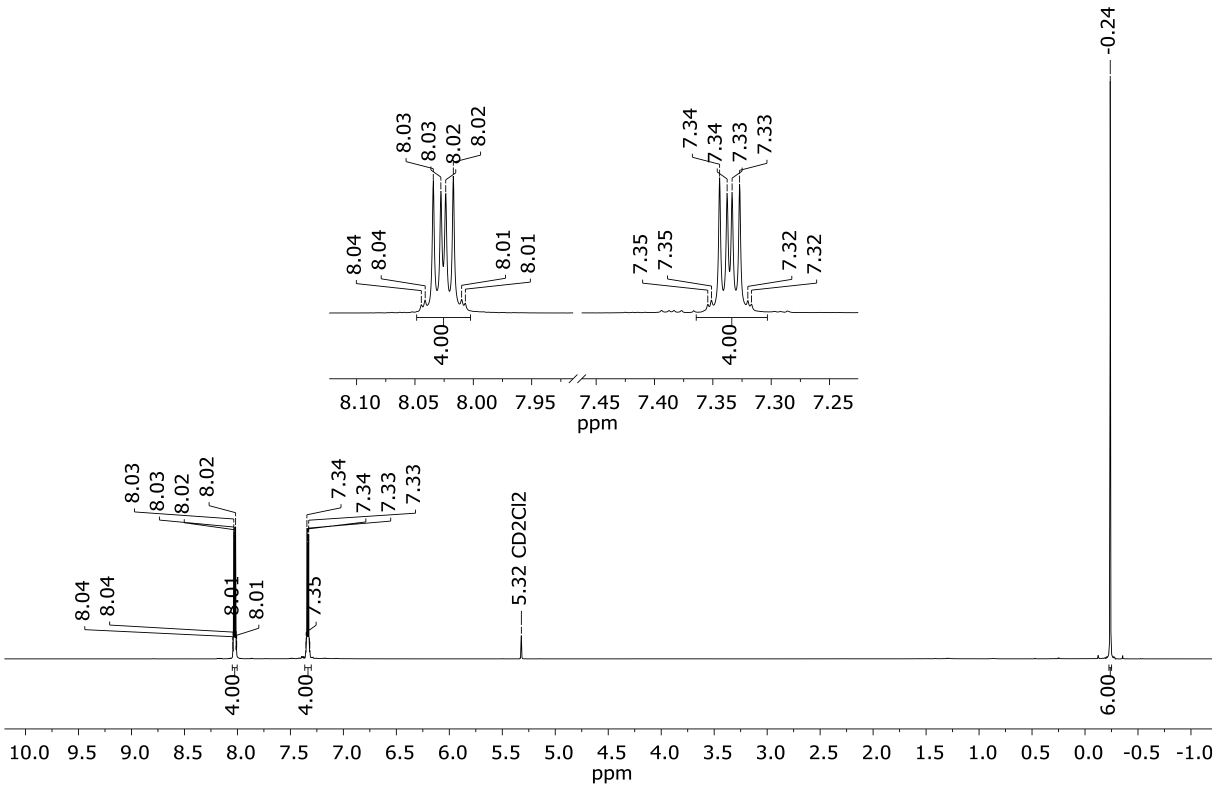

Figure S3: ^{1}H NMR spectrum of (**1**)$_2$ (500.2 MHz, CD_2Cl_2).

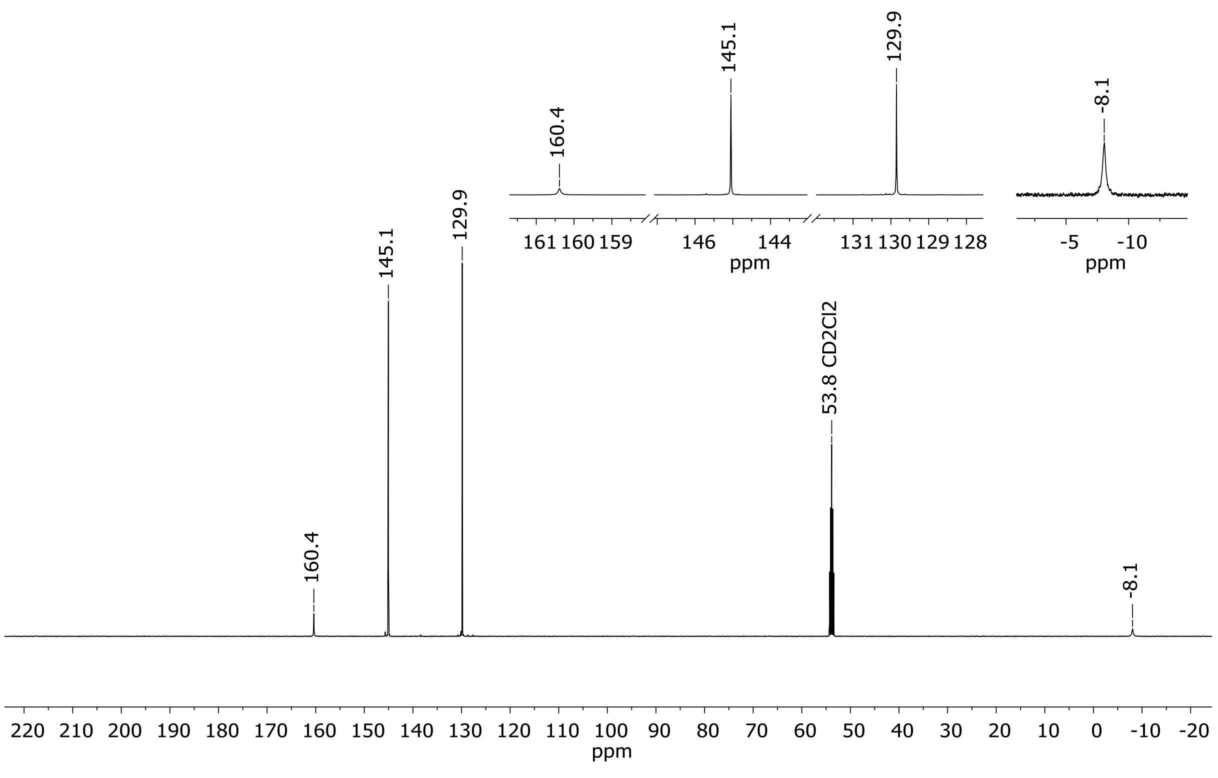

Figure S4: $^{13}C\{^{1}H\}$ NMR spectrum of (**1**)$_2$ (125.8 MHz, CD_2Cl_2).

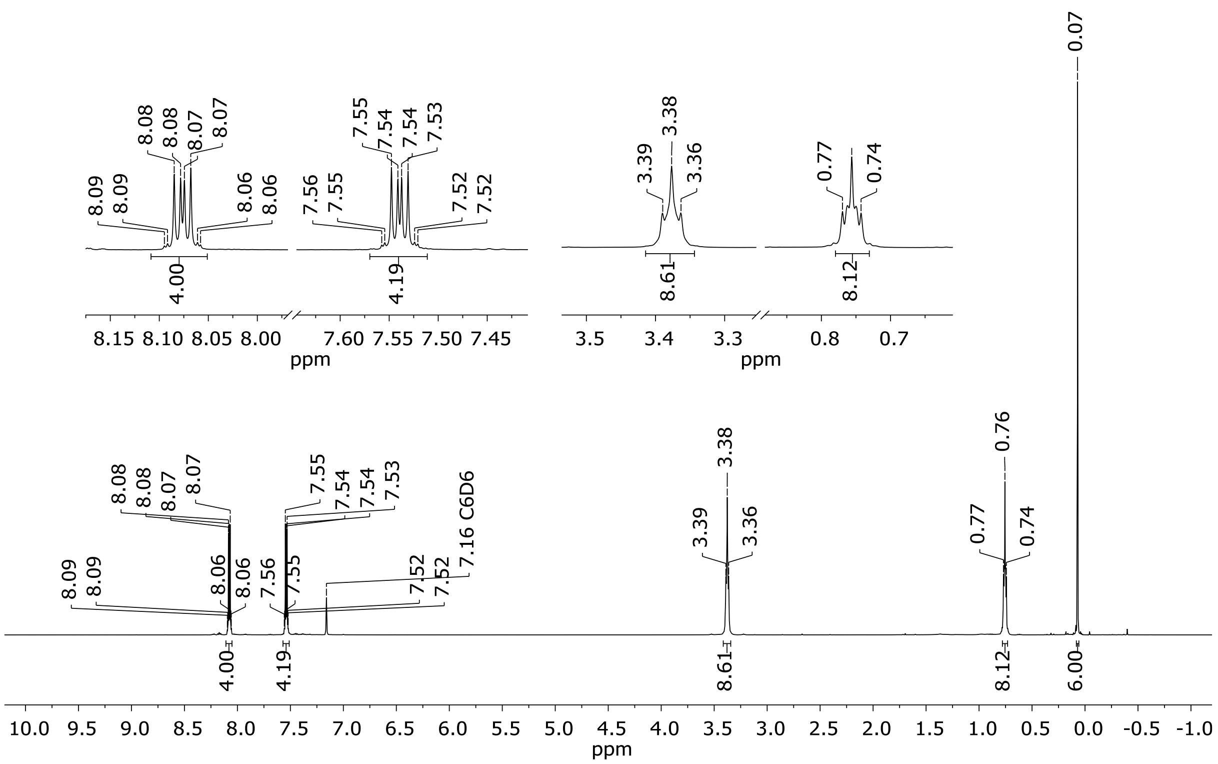

Figure S5: ^{1}H NMR spectrum of **1**·(thf)$_2$ (500.2 MHz, C_6D_6).

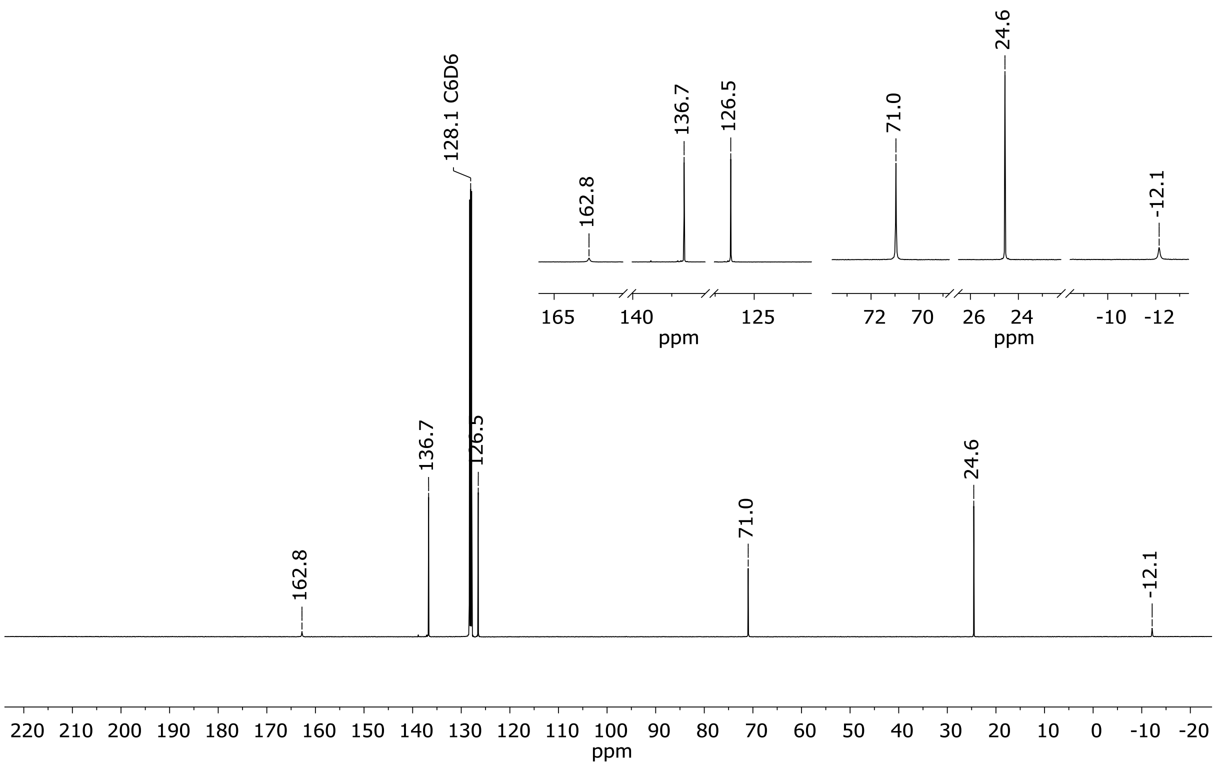

Figure S6: $^{13}C\{^{1}H\}$ NMR spectrum of **1**·(thf)$_2$ (125.8 MHz, C_6D_6).

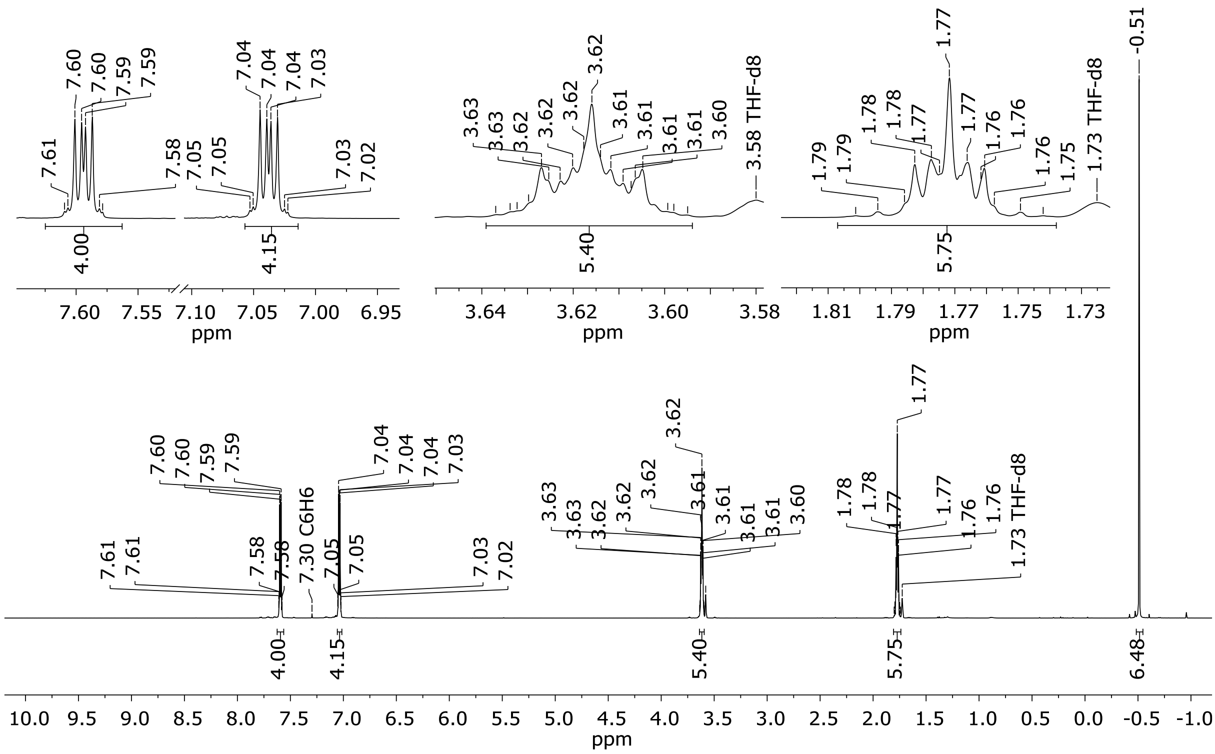

Figure S7: ^{1}H NMR spectrum of **1**·(thf)$_2$ (600.2 MHz, THF-d_8).

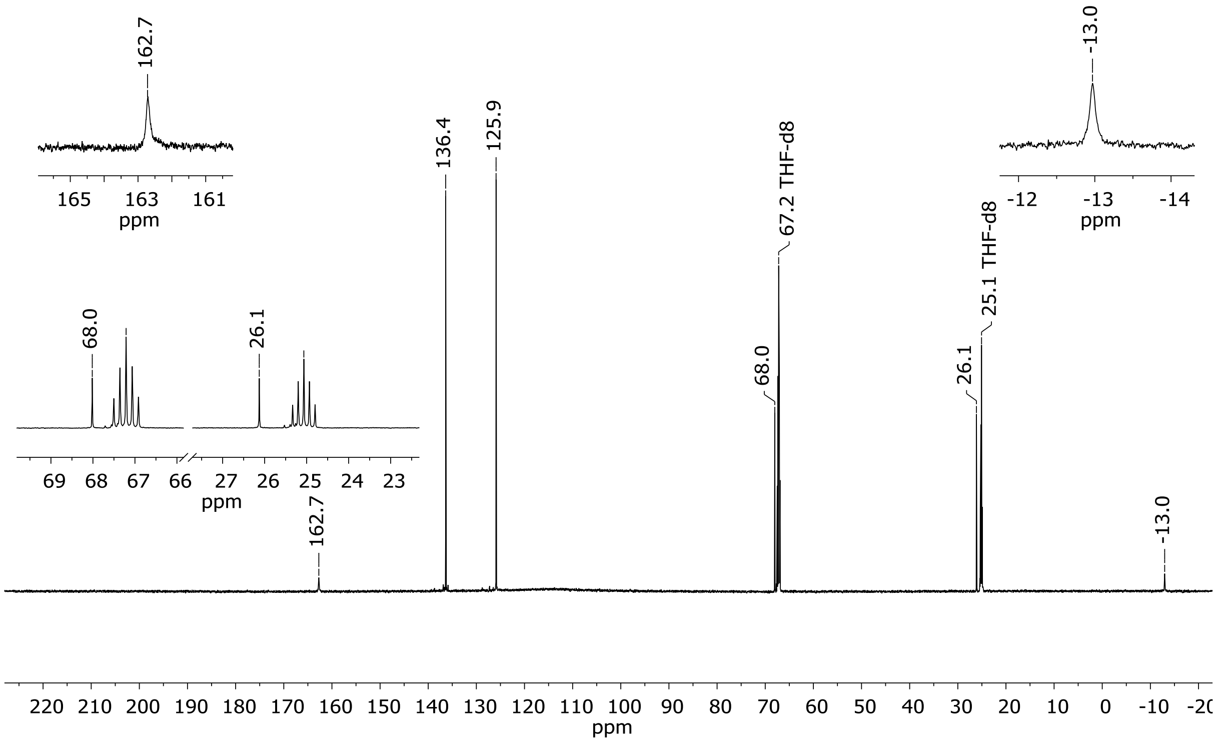

Figure S8: ^{13}C{^{1}H} NMR spectrum of **1**·(thf)$_2$ (150.9 MHz, THF-d_8).

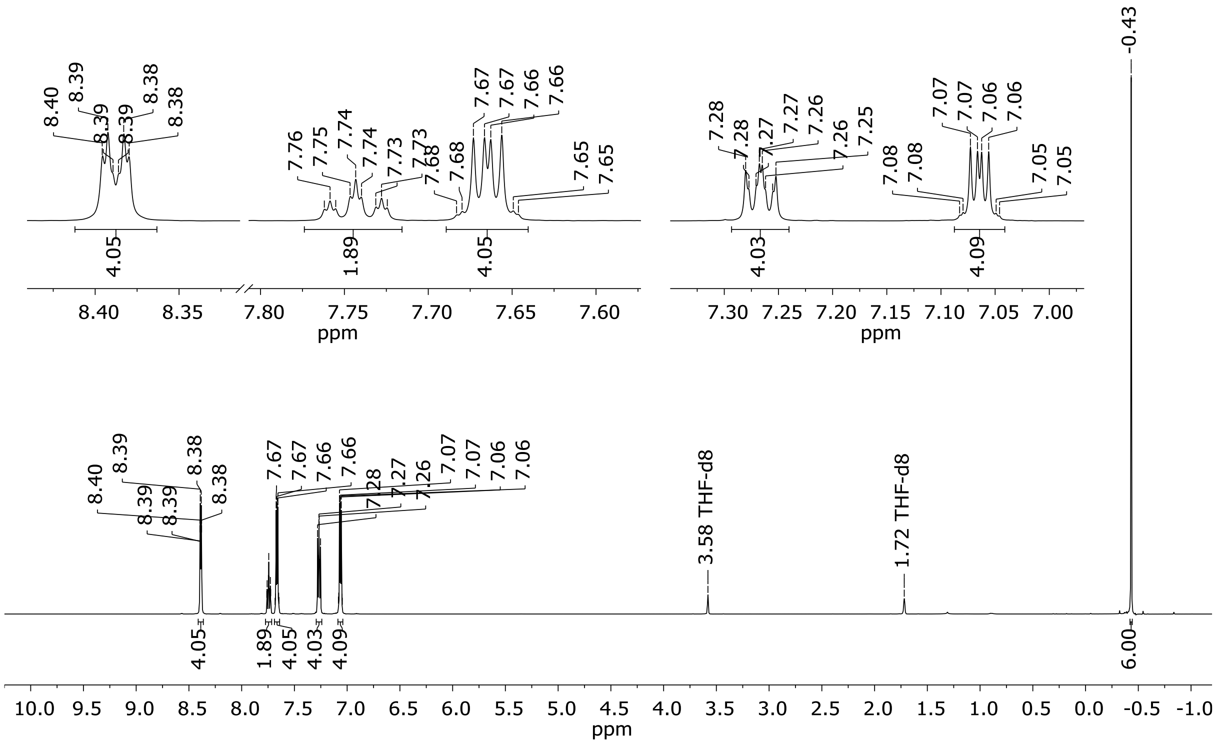

Figure S9: ^{1}H NMR spectrum of **1**·(py)$_2$ (500.2 MHz, THF-d_8).

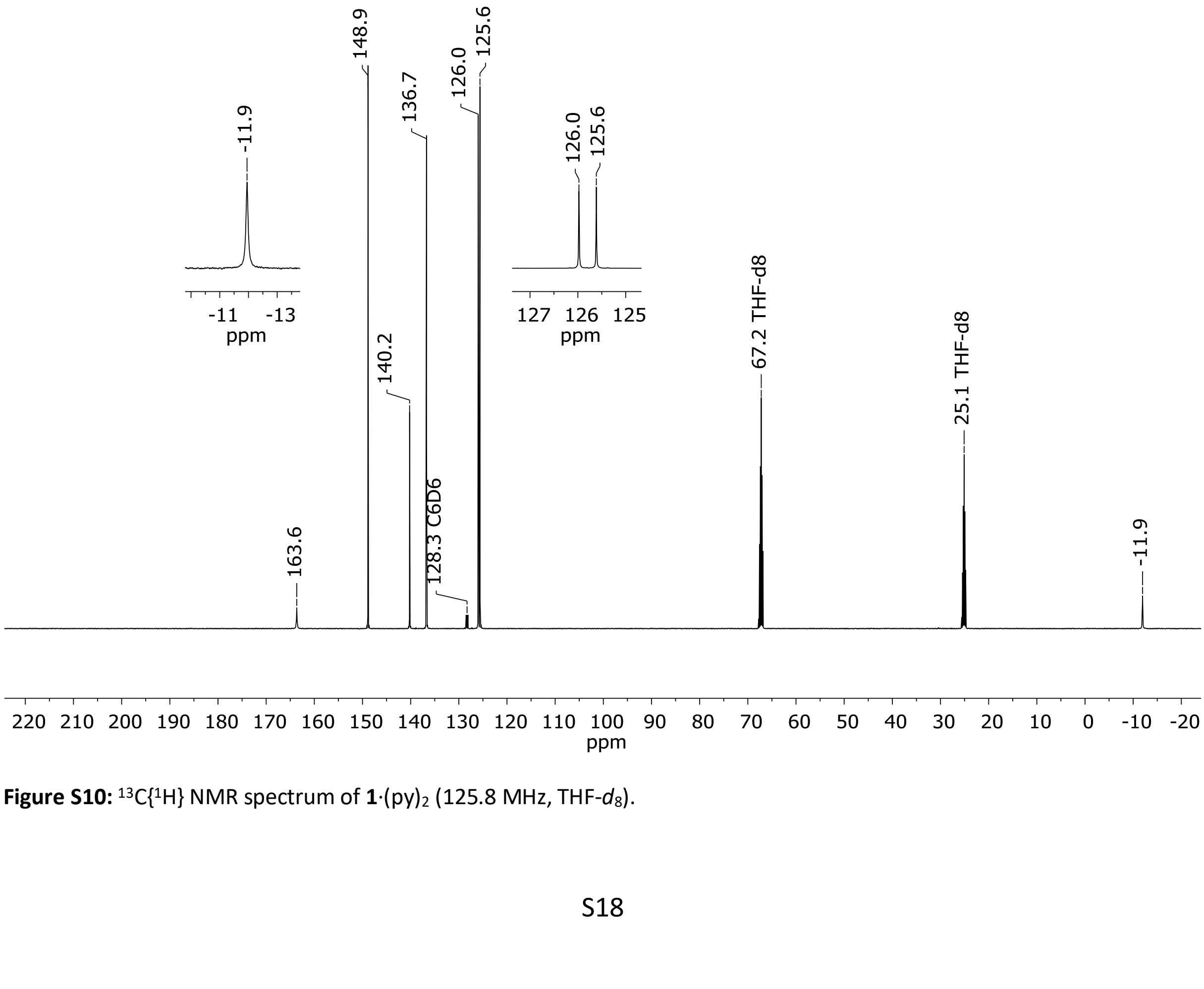

Figure S10: $^{13}C\{^{1}H\}$ NMR spectrum of **1**·(py)$_2$ (125.8 MHz, THF-d_8).

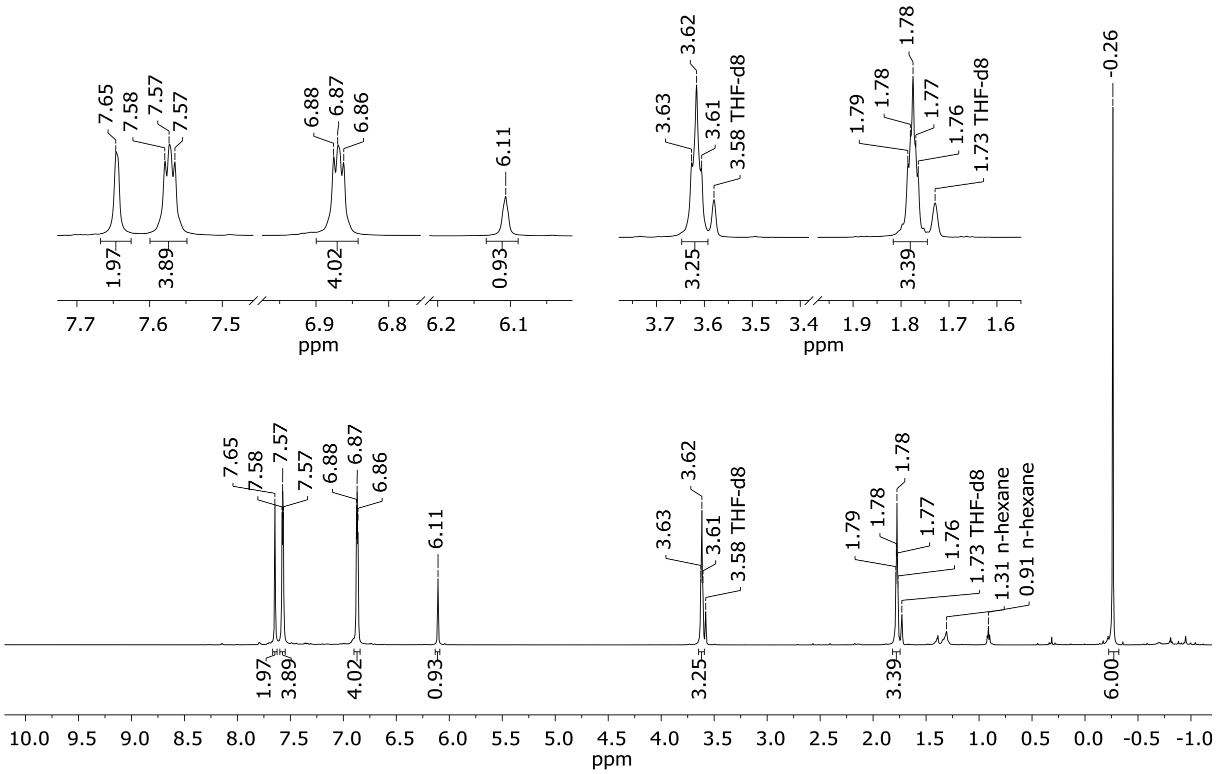

Figure S11: ^{1}H NMR spectrum of [K(thf)][**1**·(μ-pz)] (600.2 MHz, THF-d_8).

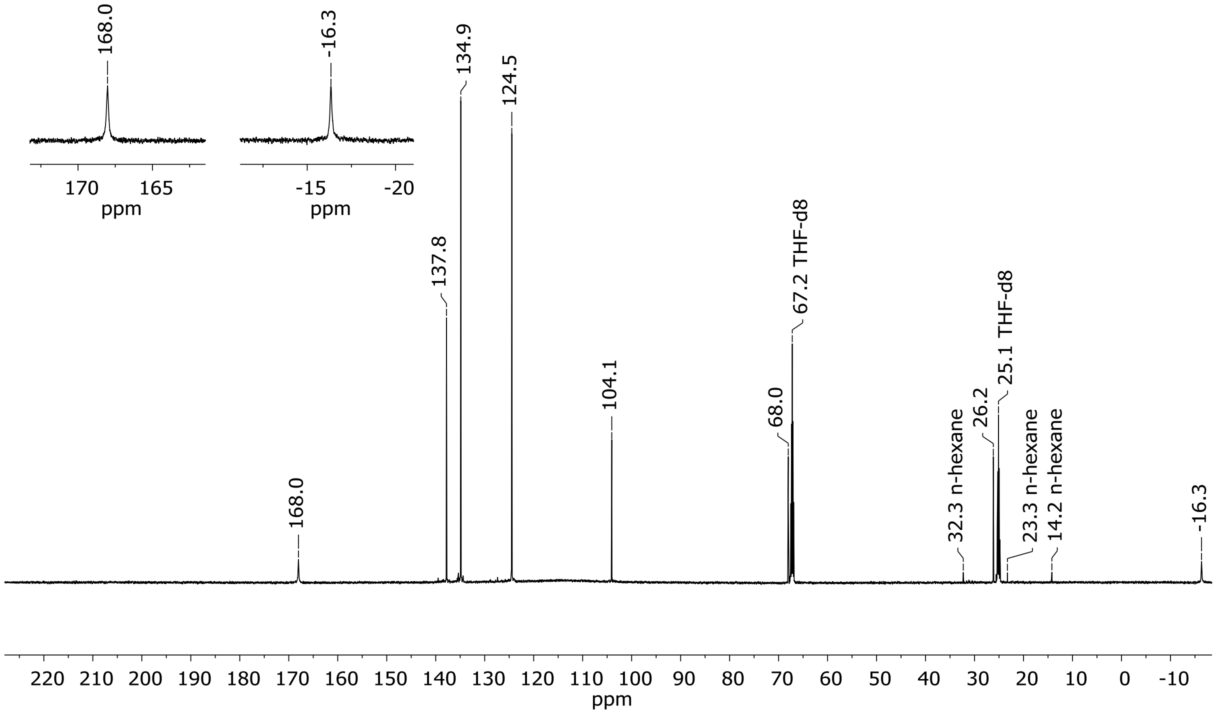

Figure S12: ^{13}C{^{1}H} NMR spectrum of [K(thf)][**1**·(μ-pz)] (150.9 MHz, THF-d_8).

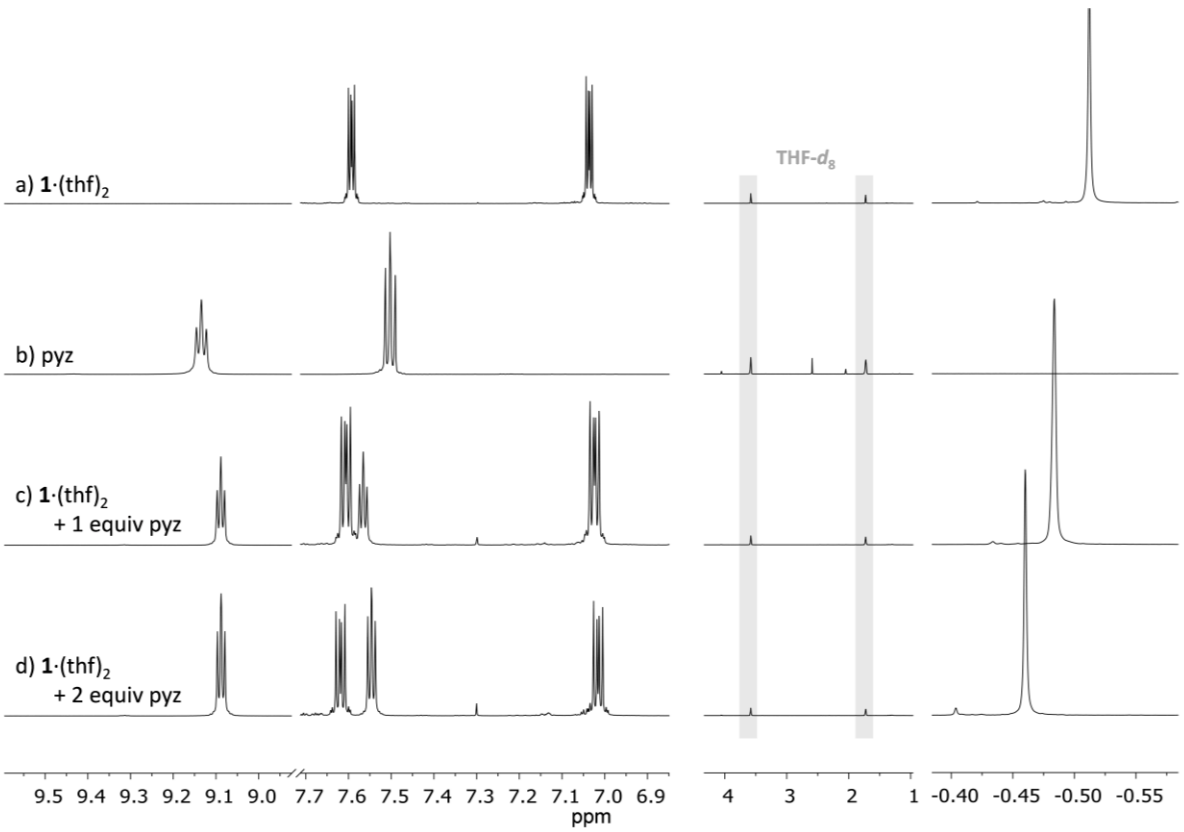

Figure S13: ^{1}H NMR spectra of a) **1**·(thf)$_2$ (600.2 MHz, THF-d_8), b) pyz (300.0 MHz, THF-d_8), c) **1**·(thf)$_2$ + 1 equiv pyz (400.3 MHz, THF-d_8), and d) **1**·(thf)$_2$ + 2 equiv pyz (400.3 MHz, THF-d_8). The intensities of the signals in the aliphatic vs. the aromatic region are not to scale. *Note*: For the sake of clarity, we refer to Al-bonded thf-d_8 ligands as "thf".

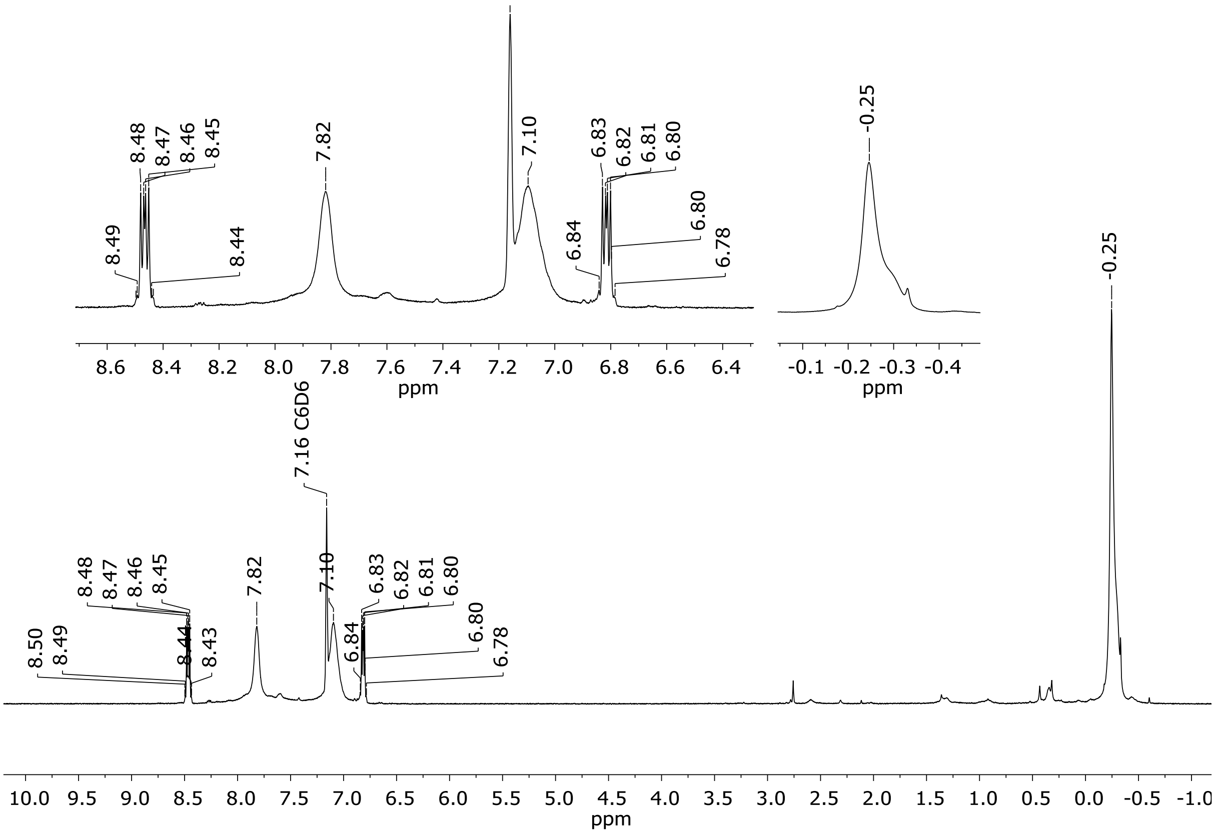

Figure S14: ^{1}H NMR spectrum of DAA-R_2·$(AlBrR_2)_2$ (R = Me or Br; 300.0 MHz, C_6D_6).

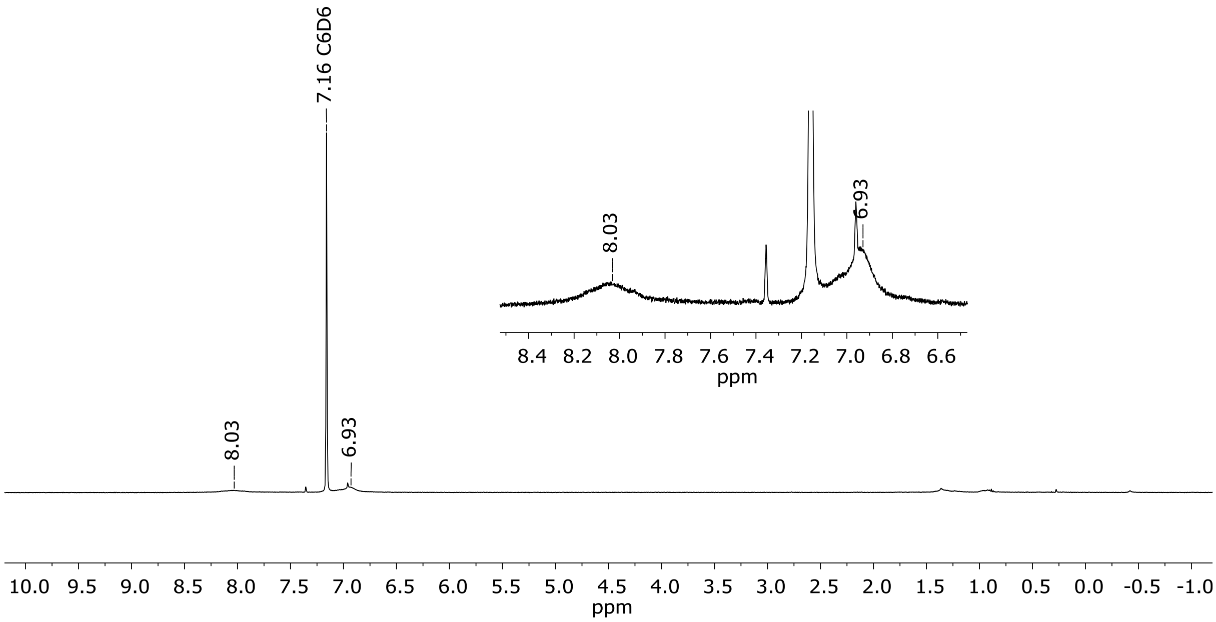

Figure S15: ^{1}H NMR spectrum of **2**·$(AlBr_3)_2$ (400.3 MHz, C_6D_6).

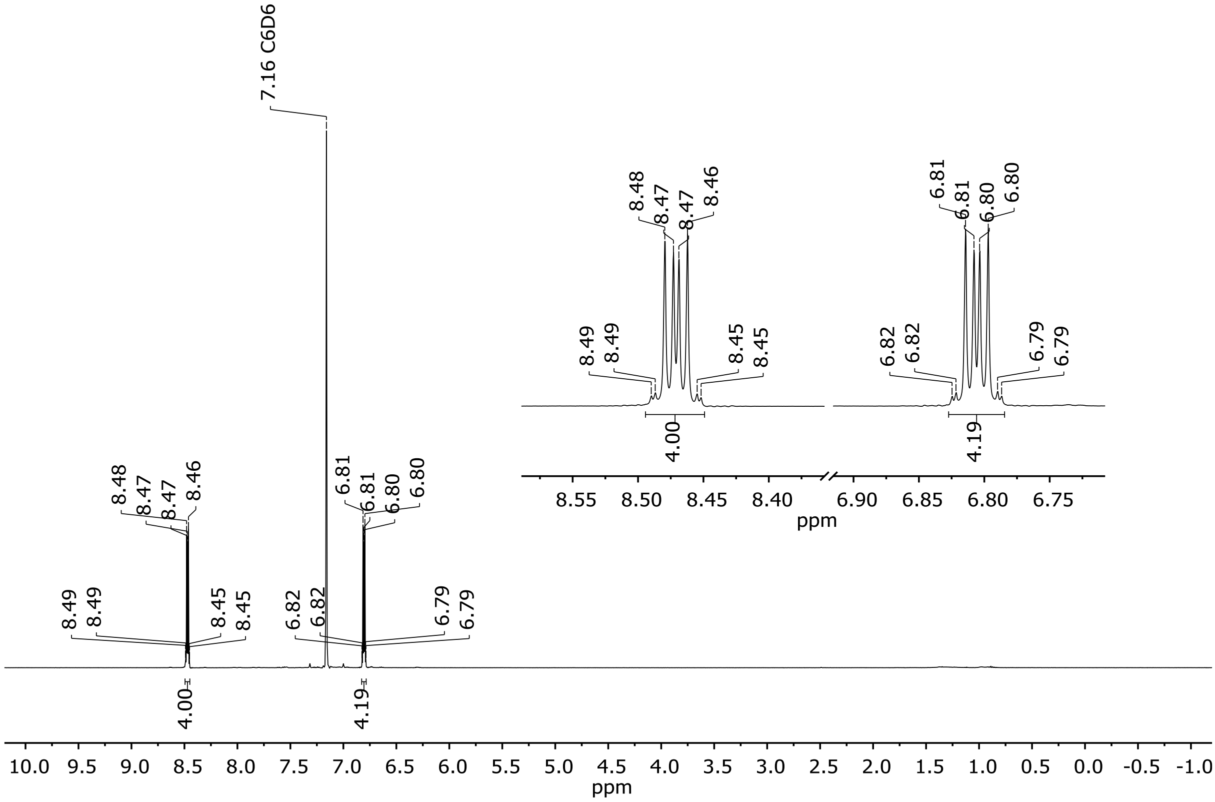

Figure S16: ^{1}H NMR spectrum of (**2**)$_2$ (500.2 MHz, C_6D_6).

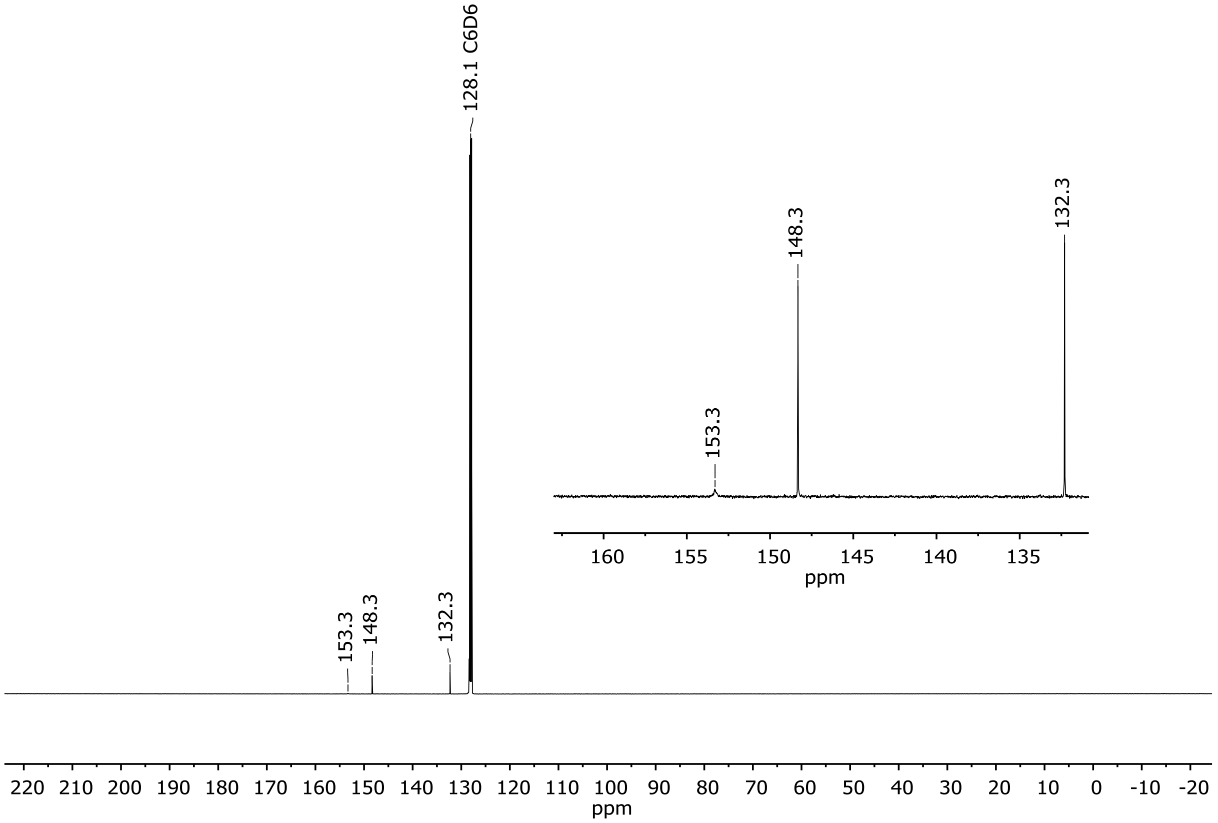

Figure S17: $^{13}C\{^{1}H\}$ NMR spectrum of (**2**)$_2$ (125.8 MHz, C_6D_6).

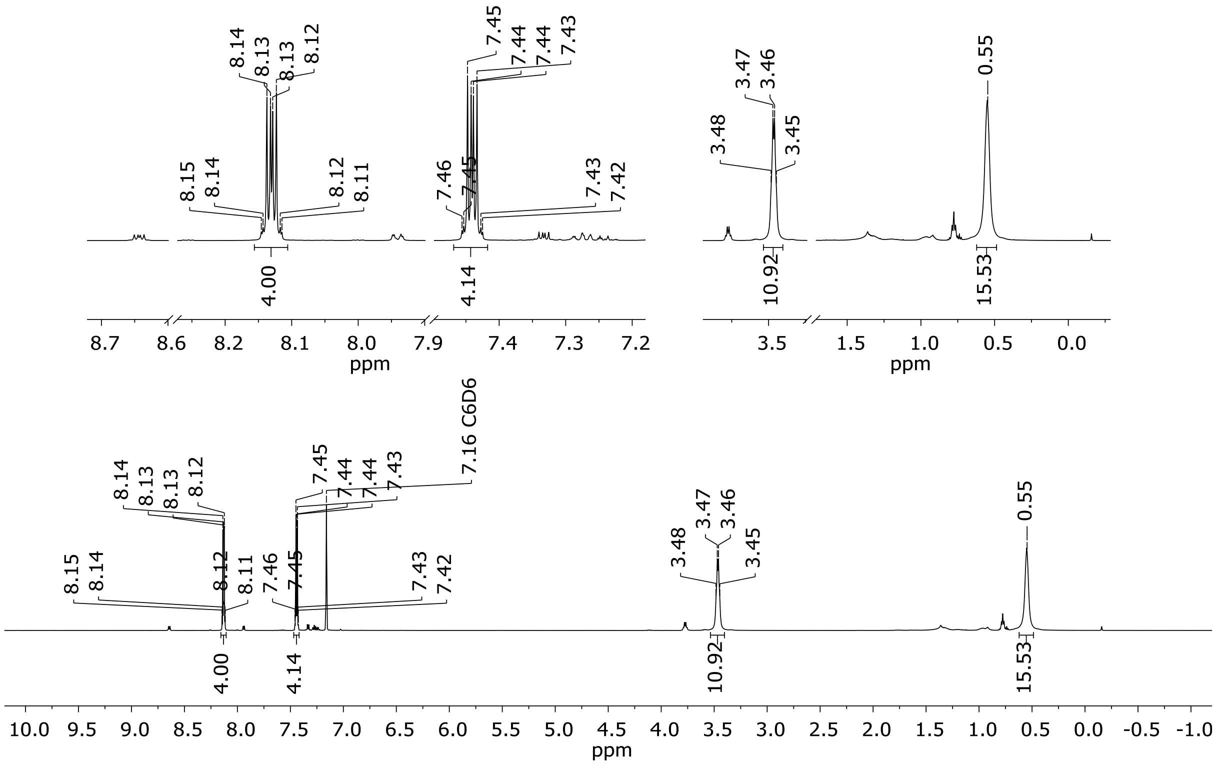

Figure S18: ^{1}H NMR spectrum of the crude product **2**·$(OEt_2)_2$ (600.2 MHz, C_6D_6).

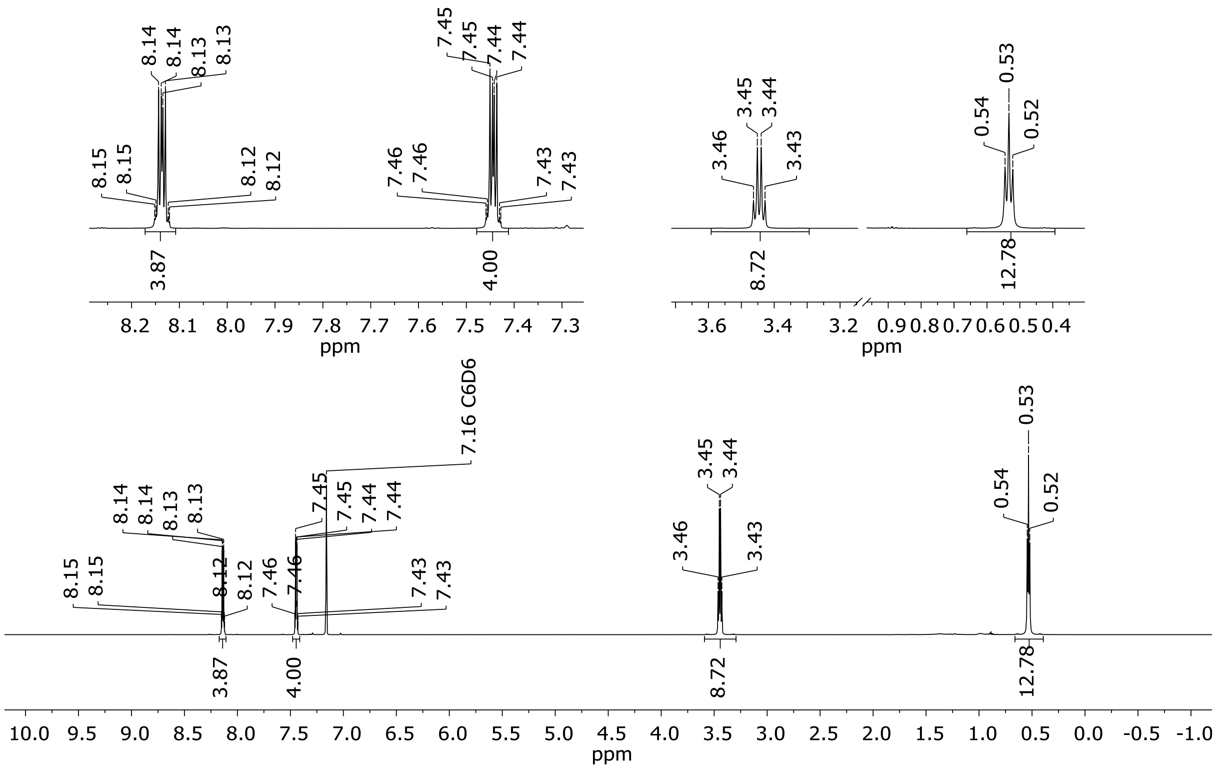

Figure S19: ^{1}H NMR spectrum of crystalline **2**·$(OEt_2)_2$ (600.2 MHz, C_6D_6).

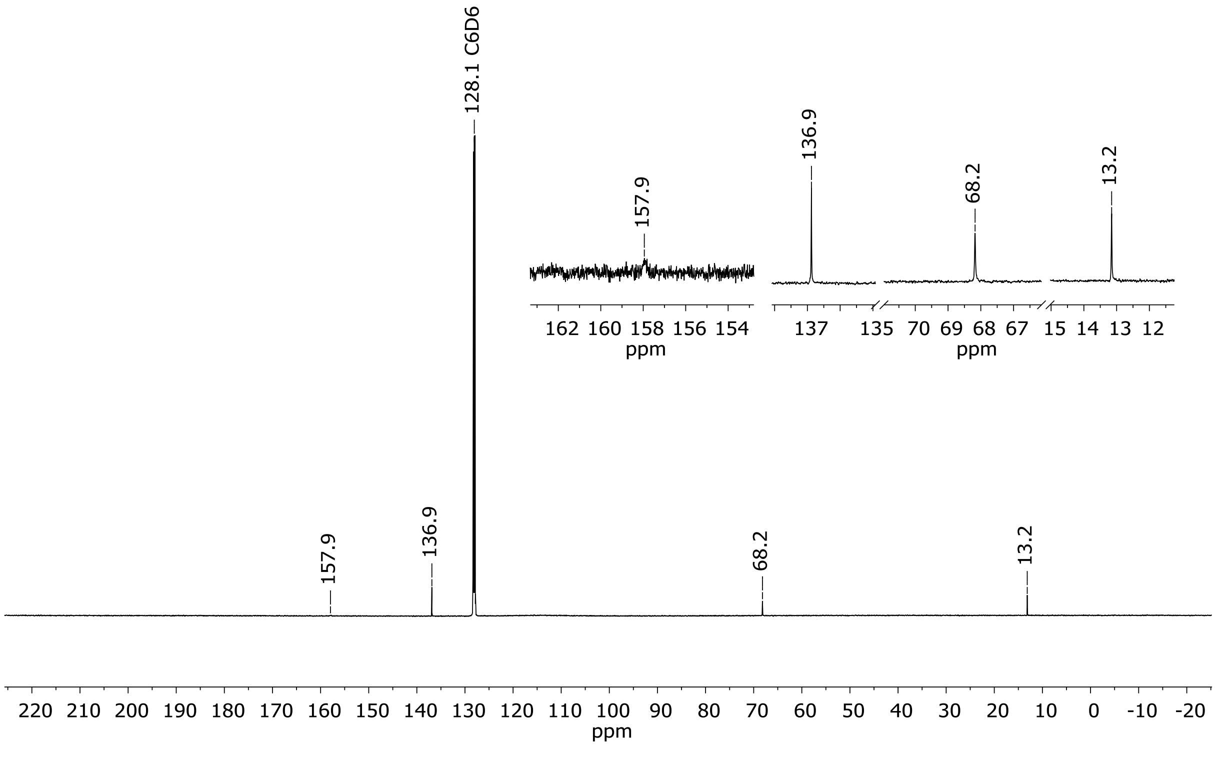

Figure S20: $^{13}C\{^{1}H\}$ NMR spectrum of **2**·$(OEt_2)_2$ (150.9 MHz, C_6D_6).

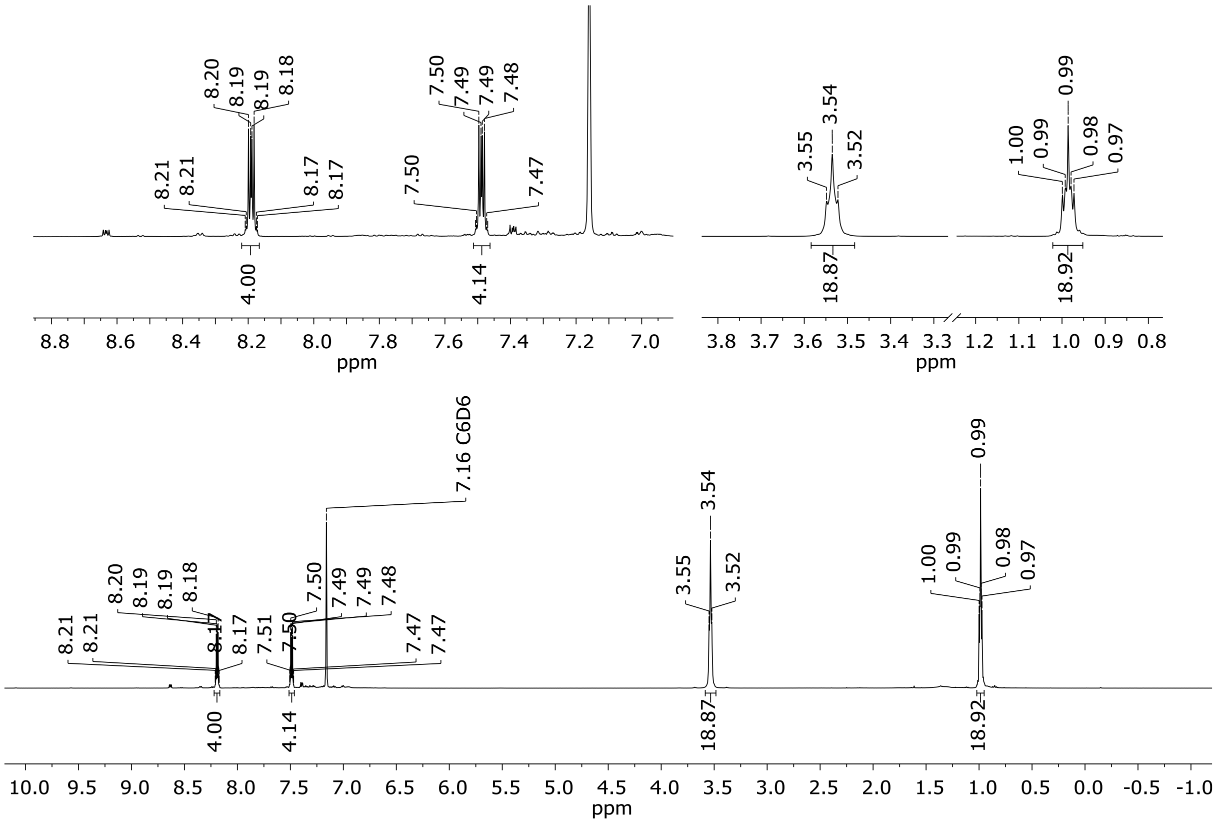

Figure S21: ^{1}H NMR spectrum of **2**·(thf)$_2$ (500.2 MHz, C_6D_6).

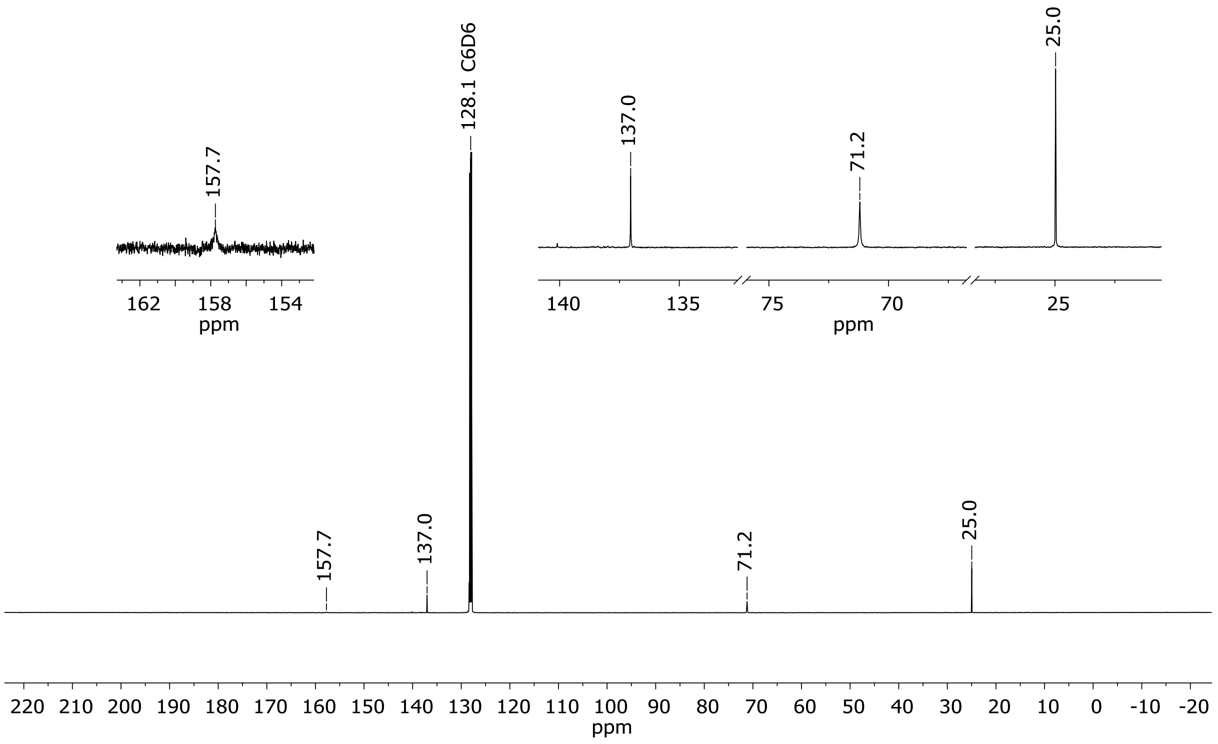

Figure S22: $^{13}C\{^{1}H\}$ NMR spectrum of **2**·(thf)$_2$ (125.8 MHz, C_6D_6).

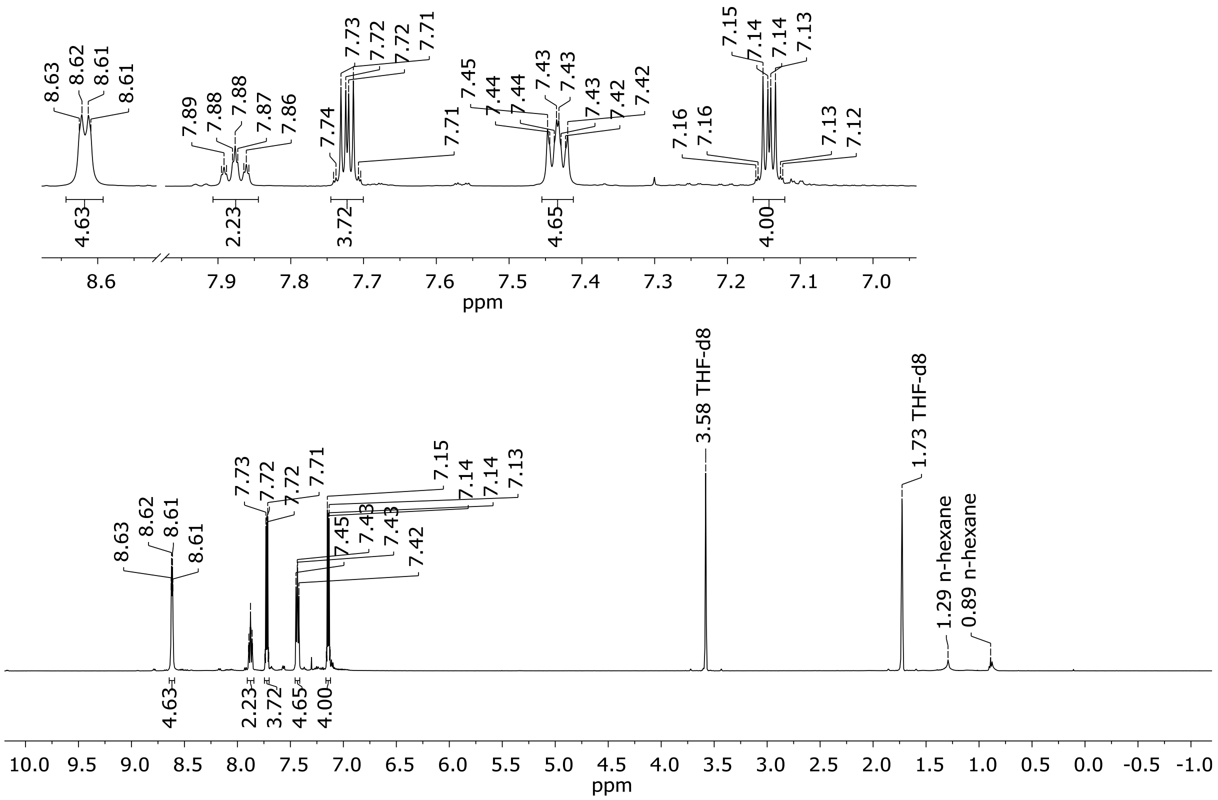

Figure S23: ^{1}H NMR spectrum of **2**·(py)$_2$ (500.2 MHz, THF-d_8).

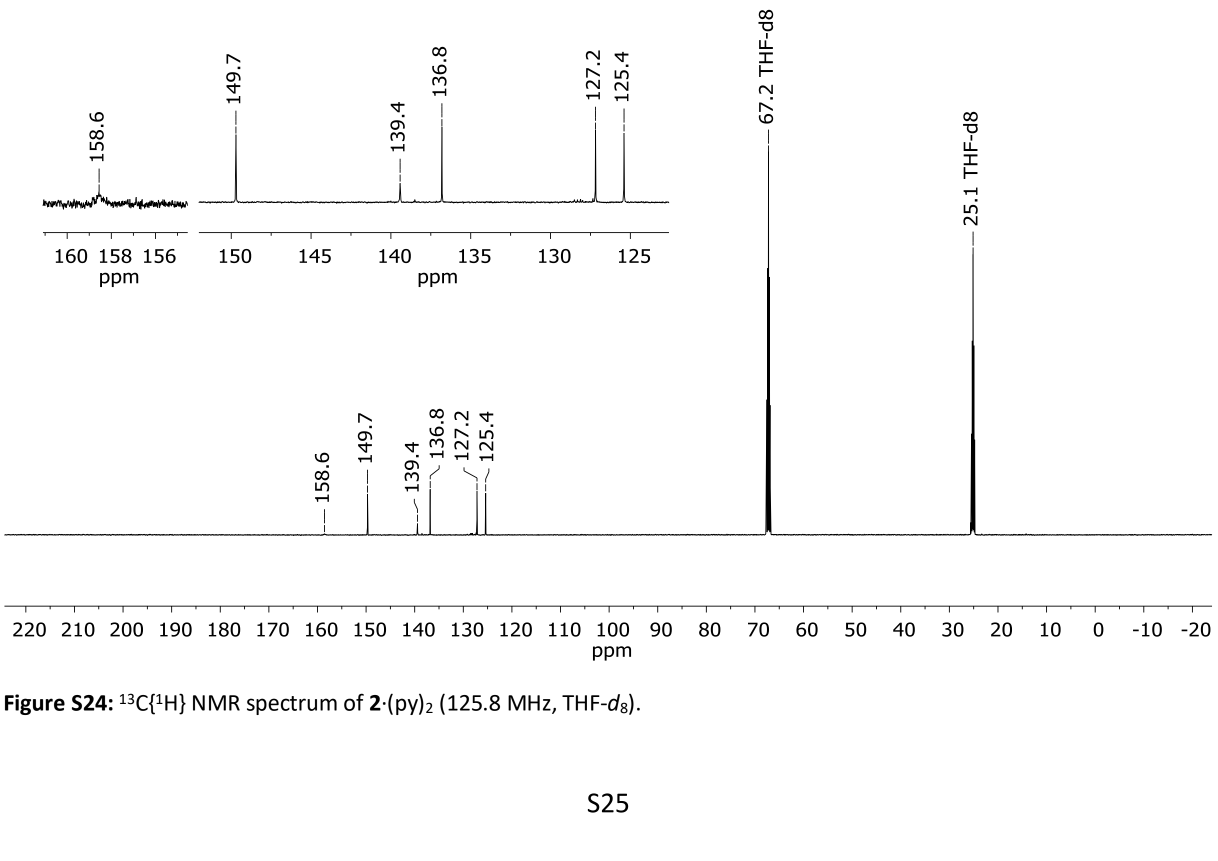

Figure S24: $^{13}C\{^{1}H\}$ NMR spectrum of **2**·(py)$_2$ (125.8 MHz, THF-d_8).

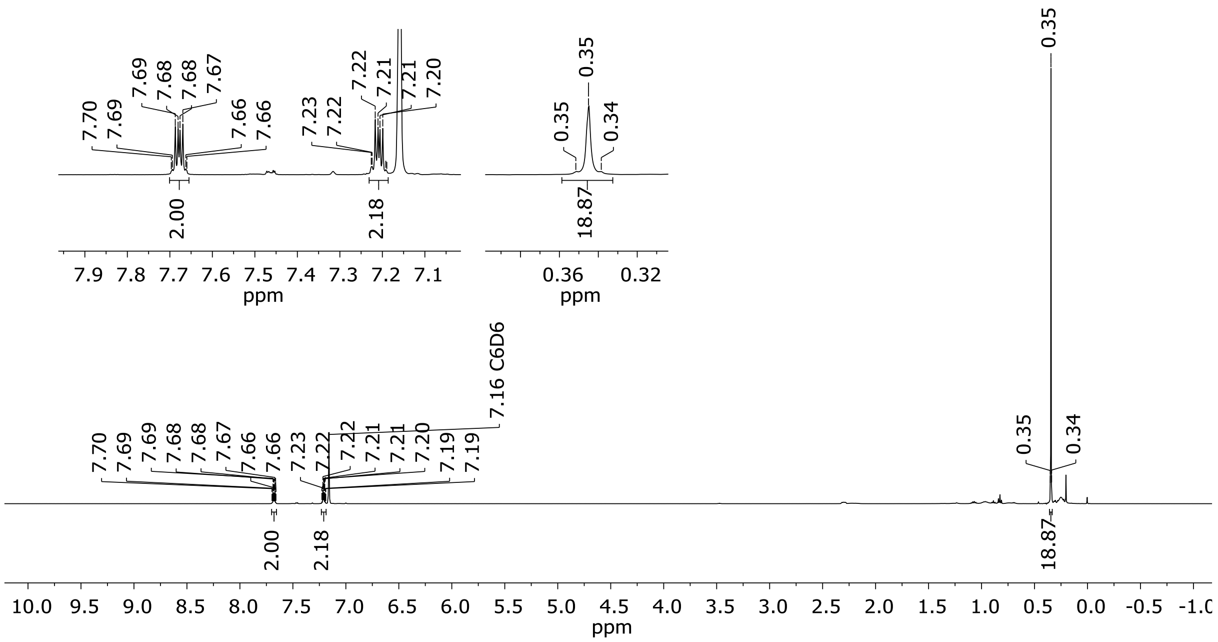

Figure S25: ^{1}H NMR spectrum (500.2 MHz, C_6D_6) of the crude product 1,2-$(Me_3Si)_2C_6H_4$, obtained via the reaction of [*n*Bu_4N][**3**] with 2 equiv Me_3SiCl.

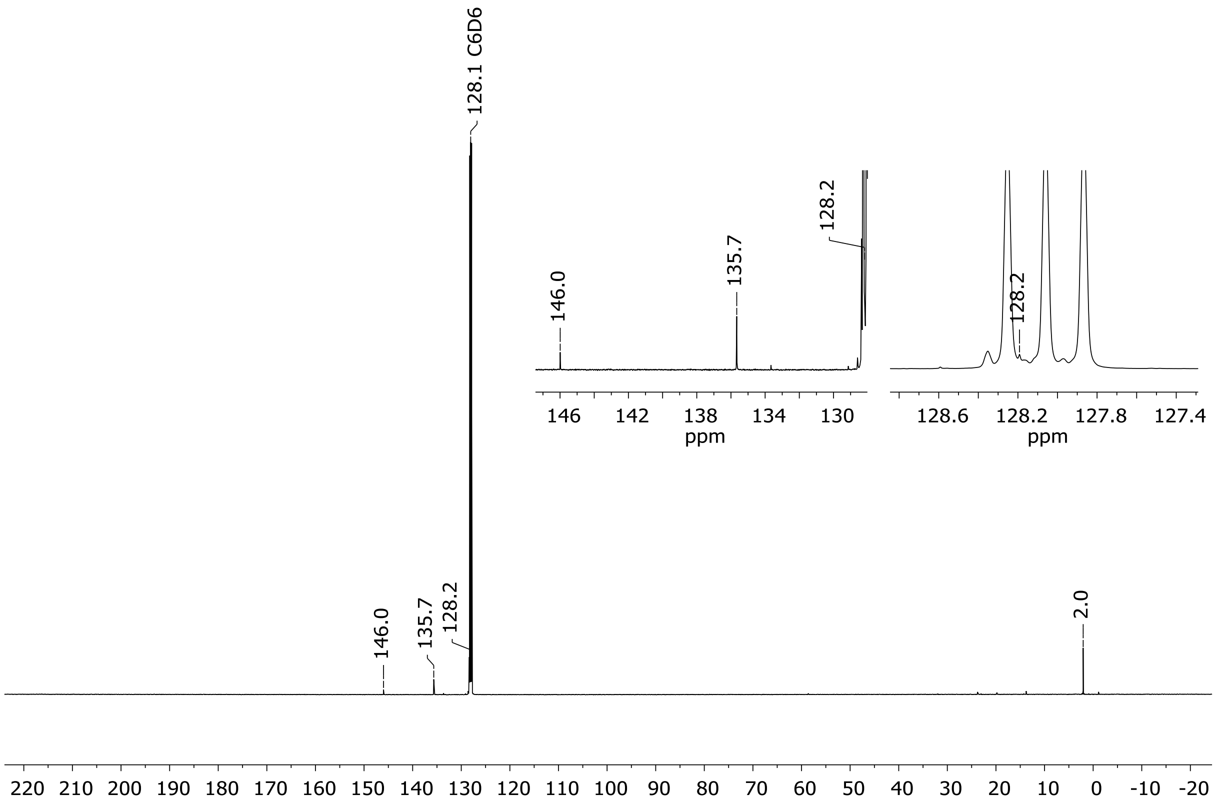

Figure S26: $^{13}C\{^{1}H\}$ NMR spectrum (500.2 MHz, C_6D_6) of the crude product 1,2-$(Me_3Si)_2C_6H_4$, obtained via the reaction of [*n*Bu_4N][**3**] with 2 equiv Me_3SiCl.

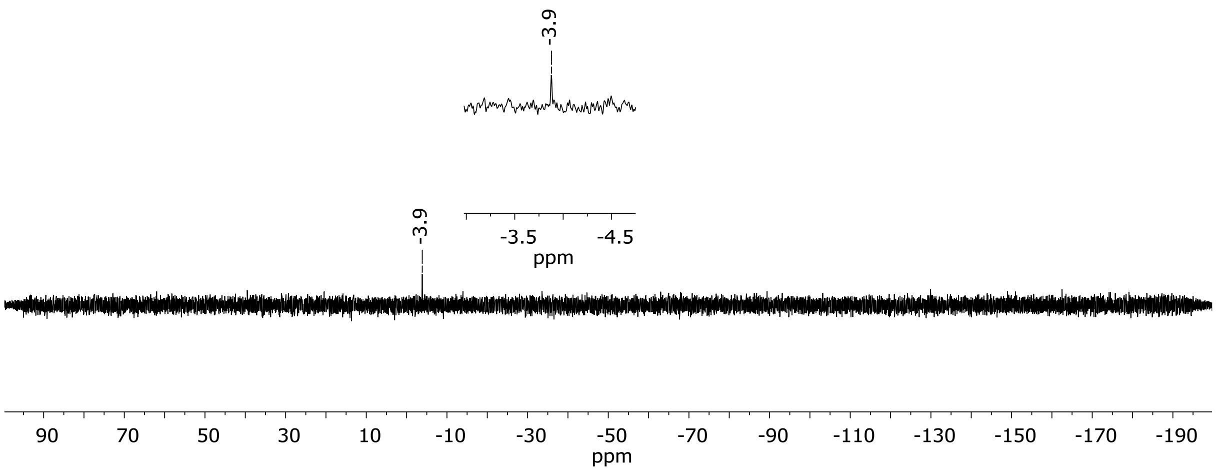

Figure S27: $^{29}Si\{^1H\}$ NMR spectrum (99.4 MHz, C_6D_6) of the crude product 1,2-$(Me_3Si)_2C_6H_4$, obtained via the reaction of [*n*Bu_4N][**3**] with 2 equiv Me_3SiCl.

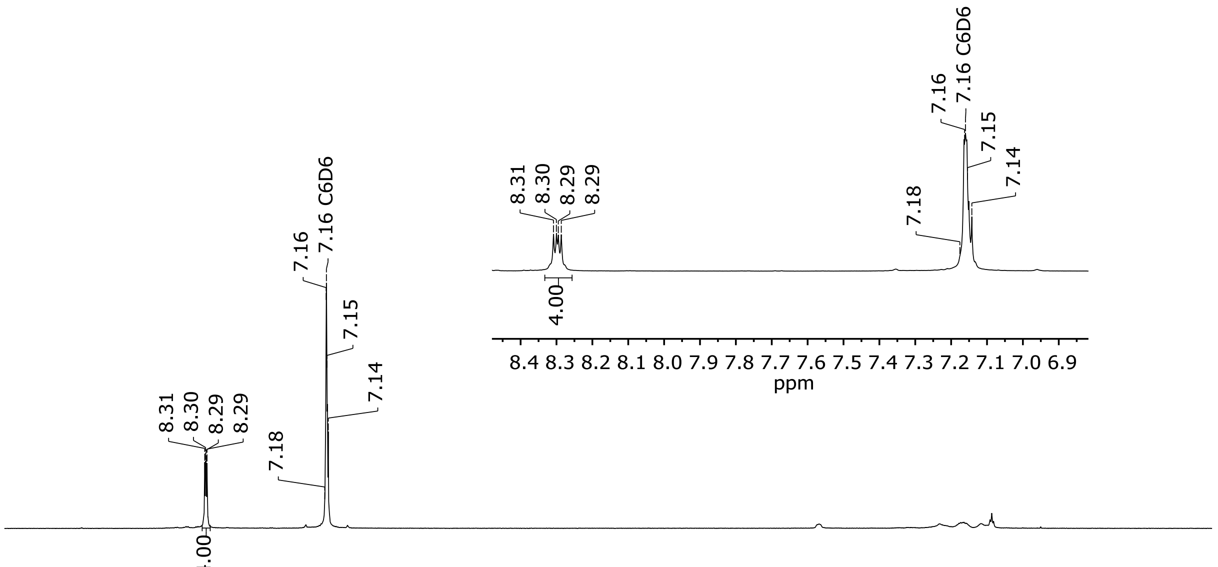

Figure S28: ^1H NMR spectrum (400.3 MHz, C_6D_6) of the product DBA-Br_2, obtained via the reaction of [*n*Bu_4N][**3**] with exc. BBr_3.

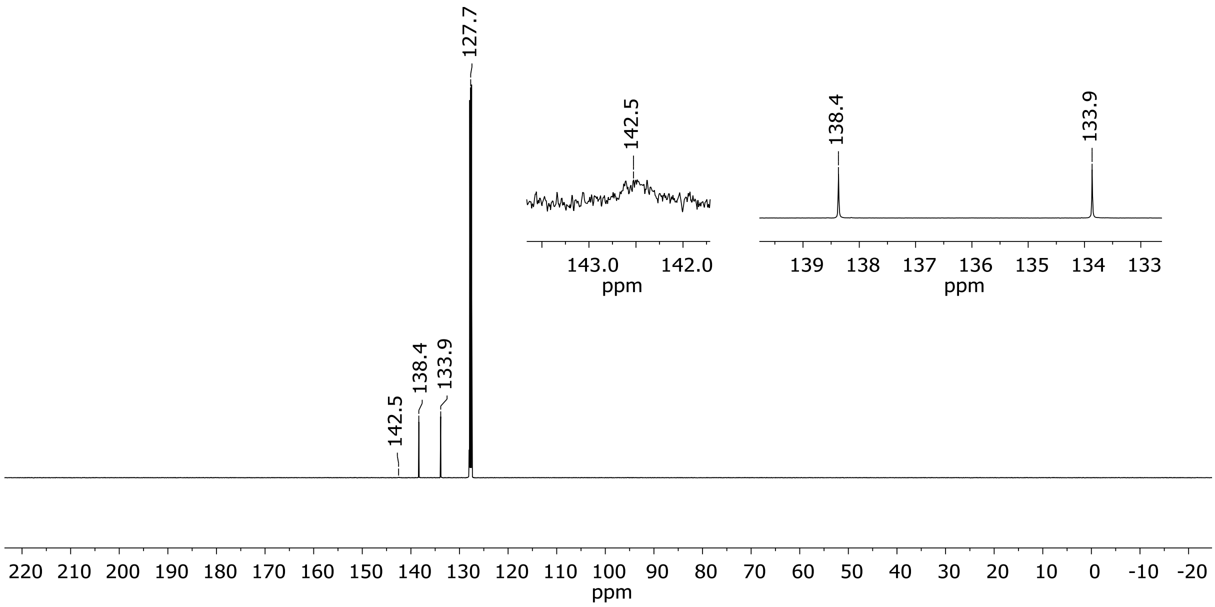

Figure S29: $^{13}C\{^{1}H\}$ NMR spectrum (125.8 MHz, C_6D_6) of the product DBA-Br_2, obtained via the reaction of [$n$$Bu_4N$][**3**] with exc. BBr_3.

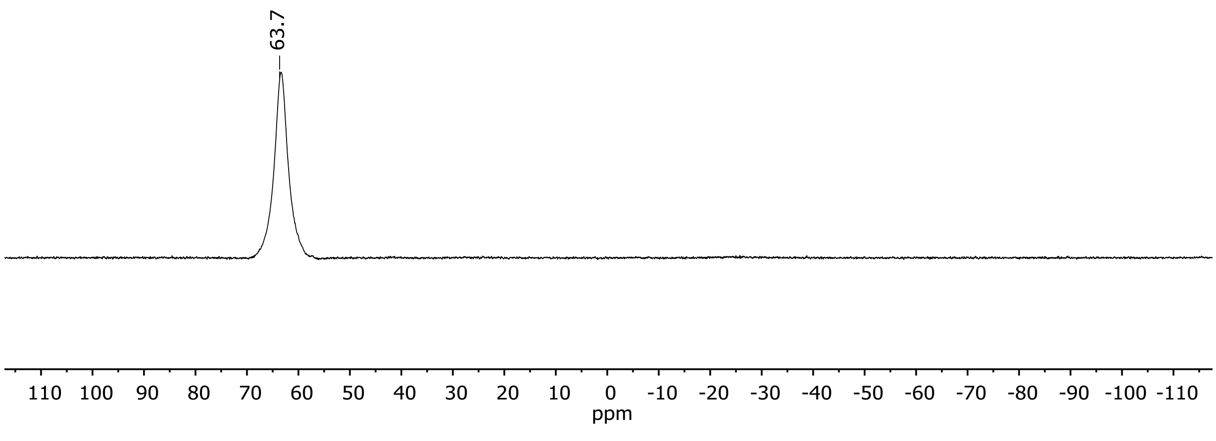

Figure S30: ^{11}B NMR spectrum (128.4 MHz, C_6D_6) of the product DBA-Br_2, obtained via the reaction of [$n$$Bu_4N$][**3**] with exc. BBr_3.

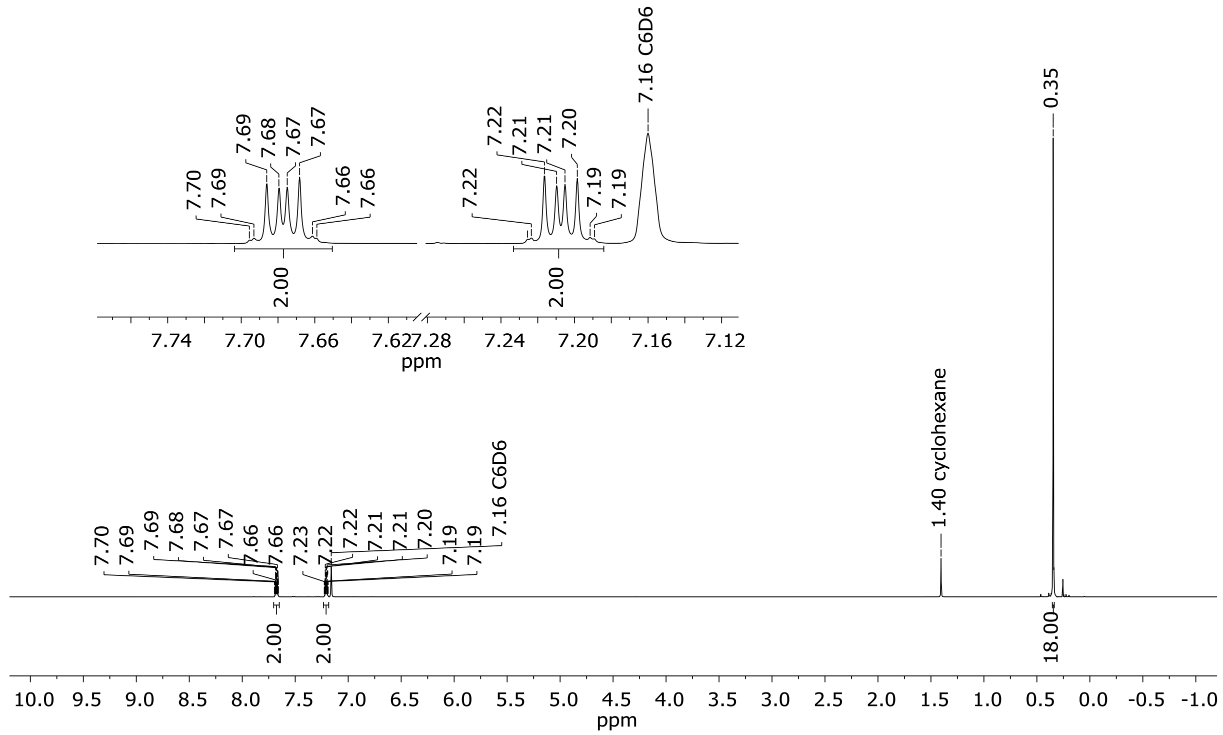

Figure S31: ^{1}H NMR spectrum of 1,2-$(Me_3Si)_2C_6H_4$ (500.2 MHz, C_6D_6).

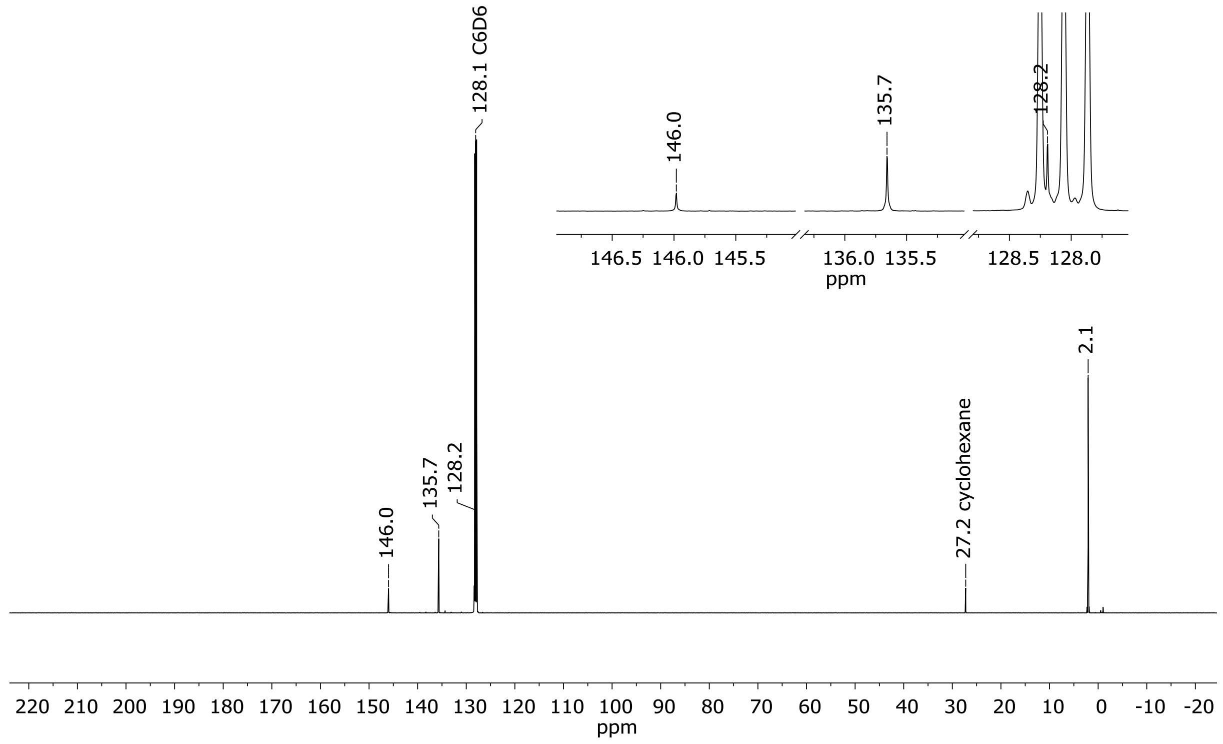

Figure S32: $^{13}C\{^1H\}$ NMR spectrum of 1,2-$(Me_3Si)_2C_6H_4$ (125.8 MHz, C_6D_6).

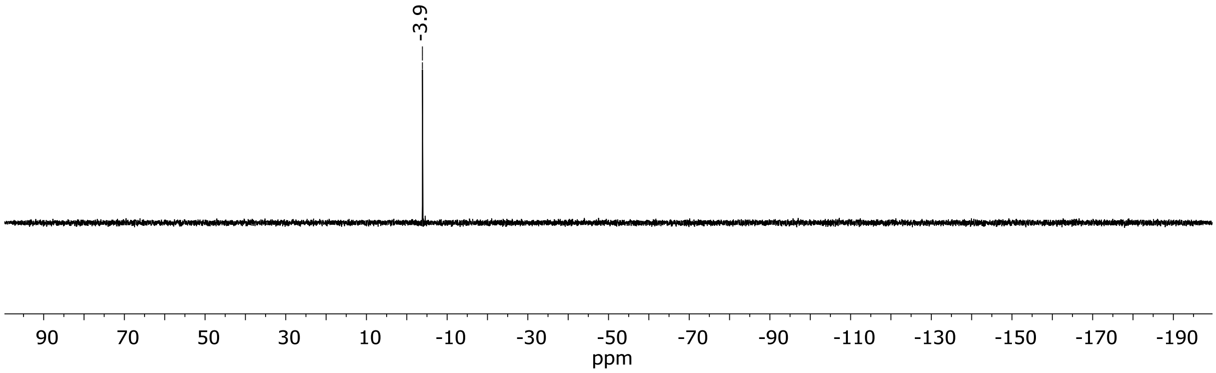

Figure S33: $^{29}Si\{^1H\}$ NMR spectrum of 1,2-$(Me_3Si)_2C_6H_4$ (99.4 MHz, C_6D_6).

3 Single-crystal X-ray structure analyses

Single-crystal diffraction data were collected at –100 °C on a *STOE IPDS II* two-circle diffractometer equipped with a *Genix 3D HS* microfocus MoK_α X-ray source (λ = 0.71073 Å). The finalization of the data, including empirical absorption corrections, was done using the *CrysAlisPro* software v.1.171.42.43a (*Rigaku Oxford Diffraction*, 2022). The structures were solved using the *SHELXS* and *SHELXT* programs and refined against $|F|^2$ with full-matrix least-squares techniques using the program *SHELXL*-2018/3.[S9,S10] All H atoms were located geometrically and refined riding on the pivot atom.

Topological aspects of the packing of molecules in crystals were analyzed with the *TOPOS 4.0 Professional* (*ToposPro*) program set.[S11]

CIF files containing the crystallographic information are deposited with the Cambridge Crystallographic Data Centre under the deposition codes CSD2385628-2385640 and can be obtained free of charge via www.ccdc.cam.ac.uk/data_request/cif. Crystallographic data and parameters of the diffraction experiments are given in Tables S1-S7.

Table S1: Selected crystallographic data for (**1**)$_2$ and **1**·(thf)$_2$.

	(**1**)$_2$	**1**·(thf)$_2$
Chemical formula	$C_{28}H_{28}Al_4$	$C_{22}H_{30}Al_2O_2$
M_r	472.42	380.42
Crystal system, space group	Triclinic, $P\bar{1}$	Monoclinic, $P2_1/n$
Temperature (K)	173	173
a, b, c (Å)	9.7750(3), 16.6702(6), 25.7105(8)	9.9086(5), 13.8047(6), 16.1315(7)
α, β, γ (°)	107.247(3), 93.692(3), 98.988(3)	90, 95.104(4), 90
V (Å^3)	3924.7(2)	2197.80(18)
Z	6	4
F(000)	1488	816
D_x (Mg m^{-3})	1.199	1.150
Radiation type	Mo Kα	Mo Kα
μ (mm^{-1})	0.19	0.14
Crystal shape	Elongated prism	Plate
Color	Colorless	Colorless
Crystal size (mm)	0.42 × 0.11 × 0.08	0.22 × 0.21 × 0.06
Absorption correction	Multi-scan	Multi-scan
T_{min}, T_{max}	0.765, 1.000	0.831, 1.000
No. of measured, independent and observed [$I > 2σ(I)$] reflections	53408, 16650, 13432	16944, 4161, 3073
R_{int}	0.065	0.048
$Θ_{max}$ (°)	26.7	25.7
Range of h, k, l	h = –12→12, k = –21→21, l = –31→32	h = –12→12, k = –16→16, l = –19→19
$R[F^2 > 2σ(F^2)]$, $wR(F^2)$, S	0.042, 0.114, 1.03	0.056, 0.161, 1.04
No. of reflections	16650	4161
No. of parameters	877	277
$Δρ_{max}$, $Δρ_{min}$ (e Å^{-3})	0.45, –0.24	0.30, –0.26

Computer programs: *X-AREA* (*Stoe & Cie*, 2001), *CrysAlis PRO* 1.171.42.43a (*Rigaku OD*, 2022), *SHELXS* (G. M. Sheldrick, 1997), *SHELXT* (G. M. Sheldrick, 2015), *SHELXL-2018*/3 (Sheldrick, 2018).

Table S2: Selected crystallographic data for **1**·(py)$_2$ × C_6H_6 and [K(thf)$_{1.5}$]$_2$[**1**·(μ-pz)]$_2$.

	**1**·(py)$_2$ × C_6H_6	[K(thf)$_{1.5}$]$_2$[**1**·(μ-pz)]$_2$
Chemical formula	$C_{24}H_{24}Al_2N_2$ × C_6H_6	$C_{46}H_{58}Al_4K_2N_4O_3$
M_r	472.52	450.54
Crystal system, space group	Triclinic, $P\bar{1}$	Triclinic, $P\bar{1}$
Temperature (K)	173	173
a, *b*, *c* (Å)	9.0697(3), 9.2595(4), 9.8024(4)	10.9074(12), 12.1287(12), 20.097(2)
α, β, γ (°)	112.117 (4), 105.052 (3), 102.588 (3)	90.030(9), 91.767(9), 109.036(9)
V (Å^3)	689.09(5)	2512.0(5)
Z	1	4
F(000)	250	363
D_x (Mg m^{-3})	1.139	2.238
Radiation type	Mo *K*α	Mo *K*α
μ (mm^{-1})	0.13	0.30
Crystal shape	Block	Block
Color	Colorless	Colorless
Crystal size (mm)	0.29 × 0.28 × 0.26	0.25 × 0.15 × 0.15
Absorption correction	Multi-scan	Multi-scan
T_{min}, T_{max}	0.848, 1.000	0.171, 1.000
No. of measured, independent and observed [$I > 2\sigma(I)$] reflections	15378, 3239, 3123	9521, 9521, 4260
R_{int}	0.031	0.204
Θ_{max} (°)	27.9	25.7
Range of *h*, *k*, *l*	*h* = –11→11, *k* = –12→12, *l* = –12→12	*h* = –13→13, *k* = –14→14, *l* = –24→24
$R[F^2 > 2\sigma(F^2)]$, $wR(F^2)$, *S*	0.039, 0.106, 1.07	0.106, 0.394, 1.11
No. of reflections	3239	9521
No. of parameters	154	537
$\Delta\rho_{max}$, $\Delta\rho_{min}$ (e Å^{-3})	0.30, –0.27	0.61, –0.74

Computer programs: *X-AREA* (*Stoe & Cie*, 2001), *CrysAlis PRO* 1.171.42.43a (*Rigaku OD*, 2022), *SHELXS* (G. M. Sheldrick, 1997), *SHELXT* (G. M. Sheldrick, 2015), *SHELXL-2018*/3 (Sheldrick, 2018).

Table S3: Selected crystallographic data for **1**·(pyz)(thf) and DAA-R_2·$(AlBrR_2)_2$ (R = Me or Br).

	**1**·(pyz)(thf)	DAA-R_2·$(AlBrR_2)_2$ (R = Me or Br)
Chemical formula	$(Al_2C_{22}H_{22}N_4)_{0.333}(Al_2C_{22}H_{26}N_2O)_{0.667}$	$C_{13.90}H_{13.69}Al_4Br_{6.10}$
M_r	391.10	776.11
Crystal system, space group	Monoclinic, $P2_1/n$	Triclinic, $P\bar{1}$
Temperature (K)	100	173
a, *b*, *c* (Å)	9.87648(19), 14.3894(3), 15.3682(3)	8.1909(9), 8.1869(9), 9.8777(10)
α, β, γ (°)	90, 104.960(2), 90	109.179(10), 102.057(9), 104.104(10)
V (Å^3)	2110.06(7)	575.79(12)
Z	4	1
F(000)	827	363
D_x (Mg m^{-3})	1.231	2.238
Radiation type	Mo $K\alpha$	Mo $K\alpha$
μ (mm^{-1})	0.15	10.78
Crystal shape	Plate	Plate
Color	Yellow	Colorless
Crystal size (mm)	0.32 × 0.25 × 0.13	0.16 × 0.13 × 0.06
Absorption correction	Multi-scan	Multi-scan
T_{min}, T_{max}	0.628, 1.000	0.219, 0.241
No. of measured, independent and observed [$I > 2\sigma(I)$] reflections	49565, 4841, 4455	5874, 2804, 2211
R_{int}	0.055	0.060
Θ_{max} (°)	27.5	28.3
Range of *h*, *k*, *l*	$h = -12 \rightarrow 12$, $k = -18 \rightarrow 18$, $l = -19 \rightarrow 19$	$h = -10 \rightarrow 10$, $k = -10 \rightarrow 10$, $l = -13 \rightarrow 13$
$R[F^2 > 2\sigma(F^2)]$, $wR(F^2)$, S	0.063, 0.158, 1.10	0.047, 0.108, 1.03
No. of reflections	4841	2804
No. of parameters	265	114
$\Delta\rho_{max}$, $\Delta\rho_{min}$ (e Å^{-3})	0.57, –0.41	0.92, –0.84

Computer programs: *X-AREA* (*Stoe & Cie*, 2001), *CrysAlis PRO* 1.171.42.43a (*Rigaku OD*, 2022), *SHELXS* (G. M. Sheldrick, 1997), *SHELXT* (G. M. Sheldrick, 2015), *SHELXL-2018*/3 (Sheldrick, 2018).

Table S4: Selected crystallographic data for α-**2**·$(AlBr_3)_2$ and β-**2**·$(AlBr_3)_2$.

	α-**2**·$(AlBr_3)_2$	β-**2**·$(AlBr_3)_2$
Chemical formula	$C_{12}H_8Al_4Br_8$	$C_{12}H_8Al_4Br_8$
M_r	899.38	899.38
Crystal system, space group	Triclinic, $P\bar{1}$	Triclinic, $P\bar{1}$
Temperature (K)	173	173
a, b, c (Å)	8.5249(4), 10.0840(6), 15.0220(7)	8.0710(5), 8.2992(6), 9.8658(7)
α, β, γ (°)	80.500(4), 80.878(4), 65.121(5)	108.901(7), 101.510(6), 103.983(6)
V (Å^3)	1149.72(11)	578.23(8)
Z	2	1
F(000)	824	412
D_x (Mg m^{-3})	2.598	2.583
Radiation type	Mo Kα	Mo Kα
μ (mm^{-1})	14.10	14.02
Crystal shape	Block	Plate
Color	Colorless	Colorless
Crystal size (mm)	0.26 × 0.23 × 0.23	0.14 × 0.09 × 0.04
Absorption correction	Multi-scan	Multi-scan
T_{min}, T_{max}	0.032, 0.051	0.145, 0.167
No. of measured, independent and observed [$I > 2\sigma(I)$] reflections	16804, 4533, 4003	6330, 2534, 2281
R_{int}	0.106	0.042
Θ_{max} (°)	26.0	27.1
Range of h, k, l	$h = -10 \rightarrow 10$, $k = -12 \rightarrow 12$, $l = -18 \rightarrow 18$	$h = -10 \rightarrow 9$, $k = -10 \rightarrow 10$, $l = -12 \rightarrow 12$
$R[F^2 > 2\sigma(F^2)]$, $wR(F^2)$, S	0.059, 0.155, 1.09	0.033, 0.087, 1.07
No. of reflections	4533	2534
No. of parameters	218	109
$\Delta\rho_{max}$, $\Delta\rho_{min}$ (e Å^{-3})	1.64, –1.57	0.79, –0.84

Computer programs: *X-AREA* (*Stoe & Cie*, 2001), *CrysAlis PRO* 1.171.42.43a (*Rigaku OD*, 2022), *SHELXS* (G. M. Sheldrick, 1997), *SHELXT* (G. M. Sheldrick, 2015), *SHELXL-2018*/3 (Sheldrick, 2018).

Table S5: Selected crystallographic data for (**2**)$_2$ and **2**·(OEt_2)$_2$.

	(**2**)$_2$	**2**·(OEt_2)$_2$
Chemical formula	$C_{24}H_{16}Al_4Br_4$	$C_{20}H_{28}Al_2Br_2O_2$
M_r	731.93	514.20
Crystal system, space group	Monoclinic, *P*2/*n*	Monoclinic, *C*2/*c*
Temperature (K)	173	90
a, *b*, *c* (Å)	10.1518(8), 9.9203(6), 12.7992(10)	19.694(3), 7.0404(6), 19.080(4)
α, β, γ (°)	90, 100.072(7), 90	90, 120.073(18), 90
V (Å^3)	1269.14(16)	2289.4(7)
Z	2	4
F(000)	704	1040
D_x (Mg m^{-3})	1.915	1.492
Radiation type	Mo *K*α	Mo *K*α
μ (mm^{-1})	6.49	3.63
Crystal shape	Prism	Prism
Color	Colorless	Colorless
Crystal size (mm)	0.22 × 0.07 × 0.06	0.21 × 0.11 × 0.08
Absorption correction	Multi-scan	Multi-scan
T_{min}, T_{max}	0.307, 1.000	0.253, 1.000
No. of measured, independent and observed [$I > 2\sigma(I)$] reflections	7388, 2682, 2361	6600, 2323, 1690
R_{int}	0.063	0.044
Θ_{max} (°)	26.8	26.4
Range of *h*, *k*, *l*	$h = -12 \rightarrow 12$, $k = -12 \rightarrow 12$, $l = -13 \rightarrow 16$	$h = -24 \rightarrow 23$, $k = -7 \rightarrow 8$, $l = -23 \rightarrow 23$
$R[F^2 > 2\sigma(F^2)]$, $wR(F^2)$, S	0.036, 0.090, 1.11	0.044, 0.098, 1.03
No. of reflections	2682	2323
No. of parameters	146	121
$\Delta\rho_{max}$, $\Delta\rho_{min}$ (e Å^{-3})	0.71, –0.43	1.59, –0.61

Computer programs: *X-AREA* (*Stoe & Cie*, 2001), *CrysAlis PRO* 1.171.42.43a (*Rigaku OD*, 2022), *SHELXS* (G. M. Sheldrick, 1997), *SHELXT* (G. M. Sheldrick, 2015), *SHELXL-2018*/3 (Sheldrick, 2018).

Table S6: Selected crystallographic data for **2**·$(thf)_2$ and **2**·$(py)_2 \times C_6H_6$.

	**2**·$(thf)_2$	**2**·$(py)_2 \times C_6H_6$
Chemical formula	$C_{20}H_{24}Al_2Br_2O_2$	$C_{22}H_{18}Al_2Br_2N_2 \times C_6H_6$
M_r	510.17	602.27
Crystal system, space group	Monoclinic, $P2_1/n$	Triclinic, $P\bar{1}$
Temperature (K)	100	173
a, *b*, *c* (Å)	9.5845(4), 13.7779(5), 16.7652(6)	9.0374(7), 9.3213(6), 9.7274(9)
α, β, γ (°)	90, 106.151(4), 90	110.307(7), 104.486(8), 104.444(6)
V (Å^3)	2126.54(15)	691.18(10)
Z	4	1
F(000)	1024	302
D_x (Mg m^{-3})	1.593	1.447
Radiation type	Mo $K\alpha$	Mo $K\alpha$
μ (mm^{-1})	3.91	3.01
Crystal shape	Plate	Block
Color	Colorless	Colorless
Crystal size (mm)	0.27 × 0.16 × 0.10	0.29 × 0.28 × 0.26
Absorption correction	Multi-scan	Multi-scan
T_{min}, T_{max}	0.535, 1.000	0.658, 1.000
No. of measured, independent and observed [$I > 2\sigma(I)$] reflections	31664, 4322, 3674	12141, 2719, 2310
R_{int}	0.062	0.053
Θ_{max} (°)	26.4	26.0
Range of *h*, *k*, *l*	$h = -11 \rightarrow 11$, $k = -17 \rightarrow 17$, $l = -20 \rightarrow 20$	$h = -11 \rightarrow 11$, $k = -11 \rightarrow 11$, $l = -12 \rightarrow 12$
$R[F^2 > 2\sigma(F^2)]$, $wR(F^2)$, S	0.033, 0.065, 1.11	0.037, 0.068, 1.16
No. of reflections	4322	2719
No. of parameters	235	181
$\Delta\rho_{max}$, $\Delta\rho_{min}$ (e Å^{-3})	0.50, –0.34	0.43, –0.34

Computer programs: *X-AREA* (*Stoe & Cie*, 2001), *CrysAlis PRO* 1.171.42.43a (*Rigaku OD*, 2022), *SHELXS* (G. M. Sheldrick, 1997), *SHELXT* (G. M. Sheldrick, 2015), *SHELXL-2018*/3 (Sheldrick, 2018).

Table S7: Selected crystallographic data for [*n*Bu_4N][**3**].

	[*n*Bu_4N][**3**]
Chemical formula	$C_{22}H_{40}Al_2Br_5N$
M_r	772.06
Crystal system, space group	Monoclinic, $P2_1$
Temperature (K)	173
a, *b*, *c* (Å)	9.4049(4), 16.9807(6), 10.3837(4)
α, β, γ (°)	90, 108.989(4), 90
V (Å^3)	1568.06 (11)
Z	2
F(000)	760
D_x (Mg m^{-3})	1.635
Radiation type	Mo Kα
μ (mm^{-1})	6.47
Crystal shape	Plate
Color	Colorless
Crystal size (mm)	0.28 × 0.14 × 0.08
Absorption correction	Multi-scan
T_{min}, T_{max}	0.142, 1.000
No. of measured, independent and observed [$I > 2\sigma(I)$] reflections	15380, 6903, 5835
R_{int}	0.056
Θ_{max} (°)	27.1
Range of *h*, *k*, *l*	$h = -12 \rightarrow 12$, $k = -21 \rightarrow 21$, $l = -13 \rightarrow 13$
$R[F^2 > 2\sigma(F^2)]$, $wR(F^2)$, S	0.042, 0.097, 1.01
No. of reflections	6903
No. of parameters	275
$\Delta\rho_{max}$, $\Delta\rho_{min}$ (e Å^{-3})	0.62, –0.54

Computer programs: *X-AREA* (*Stoe & Cie*, 2001), *CrysAlis PRO* 1.171.42.43a (*Rigaku OD*, 2022), *SHELXS* (G. M. Sheldrick, 1997), *SHELXT* (G. M. Sheldrick, 2015), *SHELXL-2018*/3 (Sheldrick, 2018).

3.1 Single-crystal X-ray structure analysis of $(1)_2$

Compound ($\mathbf{1}$)$_2$ crystallizes in the triclinic space group $P\bar{1}$ (No. 2) with three crystallographically unique molecules in general positions (Figure S34).

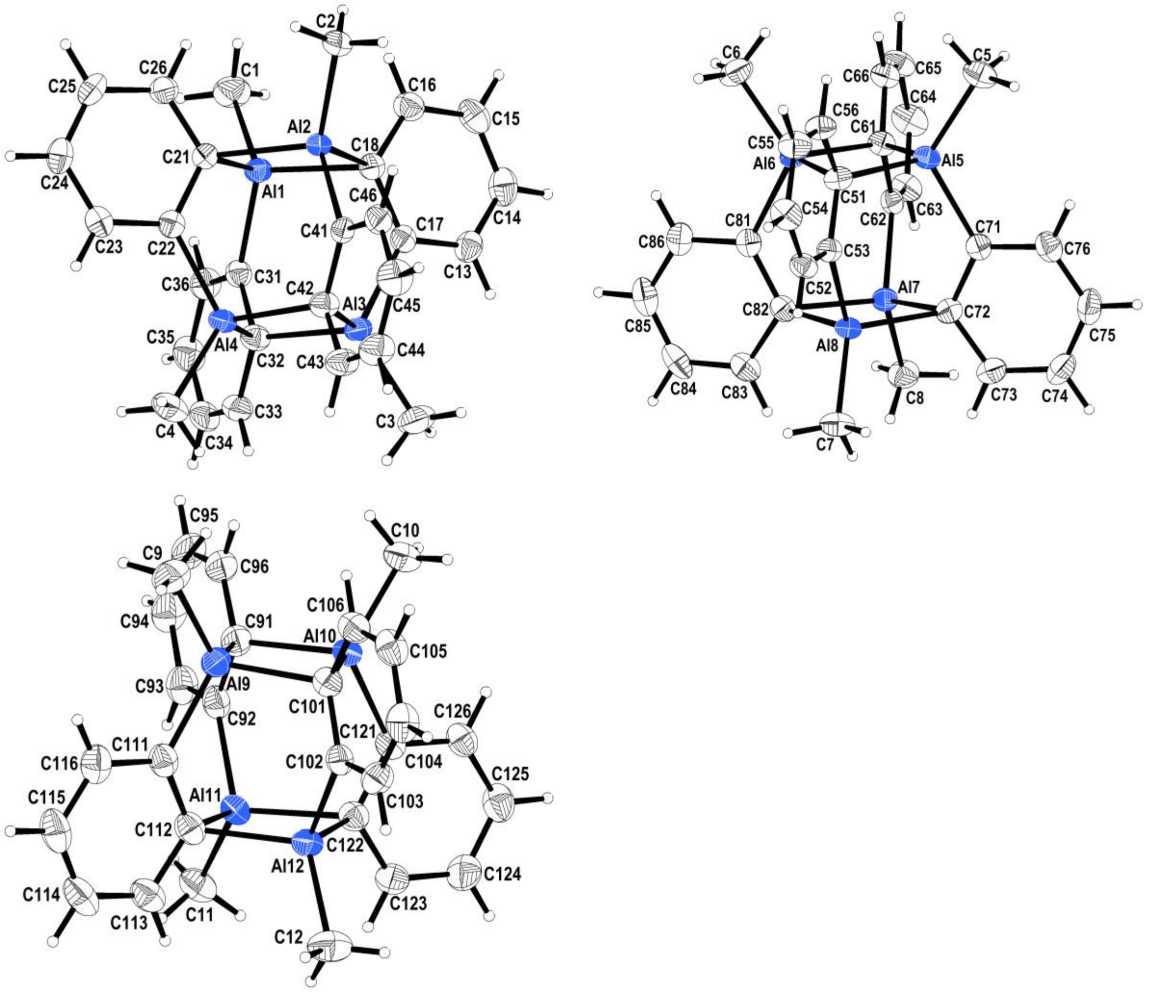

Figure S34: Molecular structures of the three unique molecules of ($\mathbf{1}$)$_2$ in the solid state. Atomic displacement ellipsoids are drawn at the 50 % probability level.

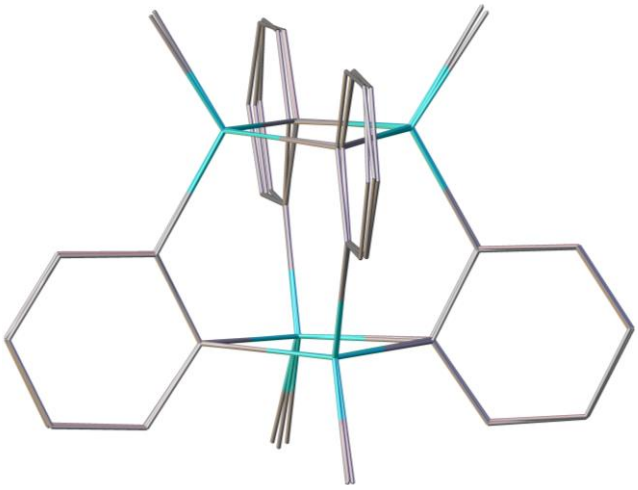

Figure S35: Overlay of the three crystallographically unique molecules ($\mathbf{1}$)$_2^A$–($\mathbf{1}$)$_2^C$ in the unit cell. H atoms omitted for clarity. C: grey, Al: turquoise.

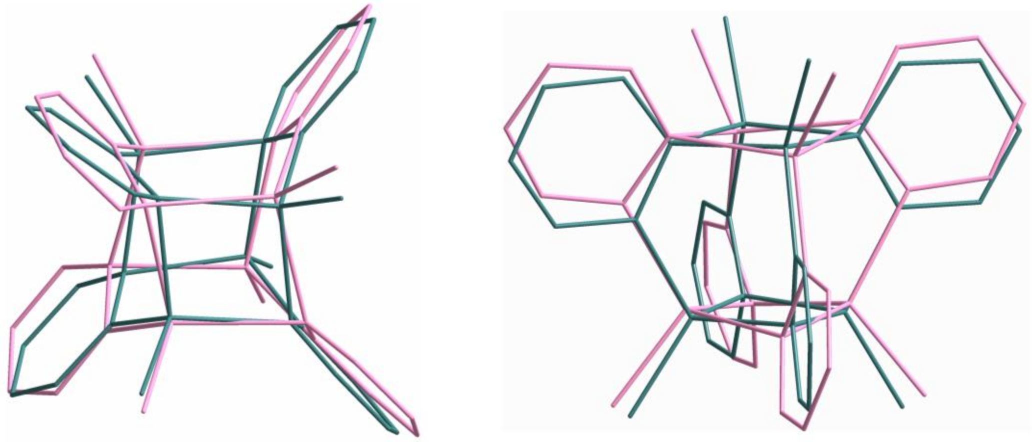

Figure S36: Overlay of (**1**)$_2$ (turquoise) and $[(C_6H_4){-}Mg(thf)]_4$ (pink); the thf ligands are represented by their O atoms. H atoms omitted for clarity.

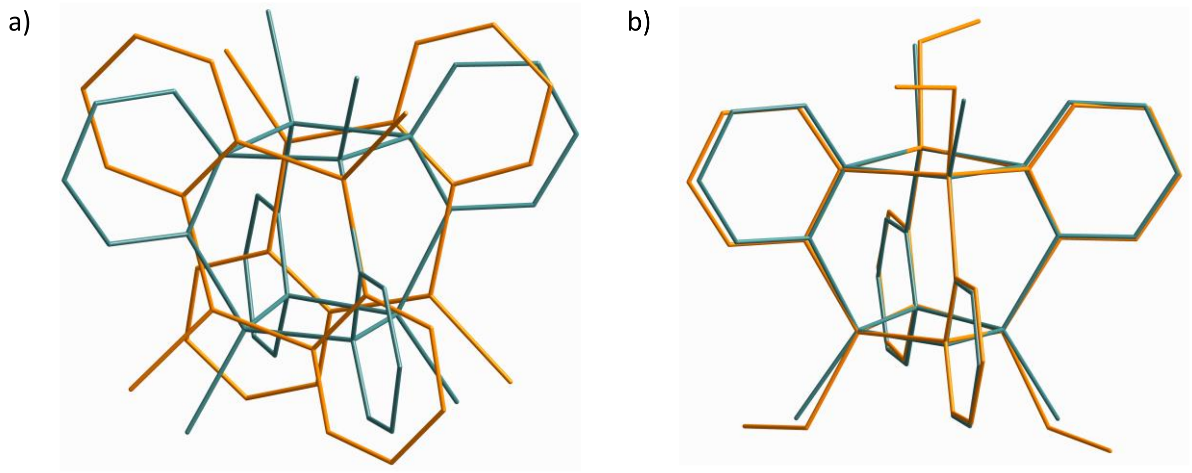

Figure S37: Overlay of (**1**)$_2$ (turquoise) with a) DGA-Me_2 (orange) and b) DGA-Et_2 (orange). H atoms omitted for clarity.

3.2 Single-crystal X-ray structure analysis of 1·(thf)$_2$

Compound **1**·(thf)$_2$ crystallizes in the monoclinic space group $P2_1/n$ (No. 14) with two crystallographically unique molecules at the inversion centers (Figure S38). The thf ligands are disordered over three positions with the relative weights of 43:36:21 %. The corresponding carbon atoms were refined by the isotropic approximation.

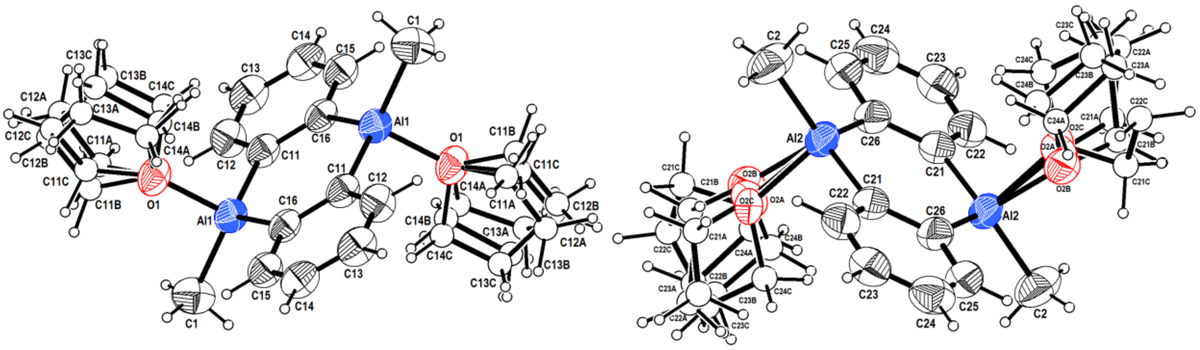

Figure S38: Molecular structures of the two unique molecules of **1**·(thf)$_2$ in the solid state. Atomic displacement ellipsoids are drawn at the 50 % probability level. The disordered positions of the isotropically refined carbon atoms are depicted as spheres.

3.3 Single-crystal X-ray structure analysis of 1·(py)$_2$ × C_6H_6

The solvate **1**·(py)$_2$ crystallizes with one C_6H_6 molecule in the triclinic space group $P\bar{1}$ (No. 2) (Figure S39). Both molecules are located at inversion centers.

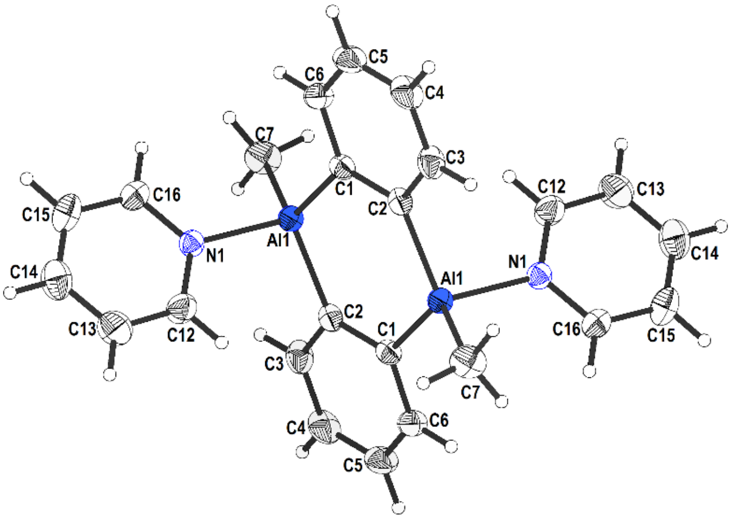

Figure S39: Molecular structure of **1**·(py)$_2$ × C_6H_6 in the solid state. Atomic displacement ellipsoids are drawn at the 50 % probability level. The solvent molecule (C_6H_6) is omitted for clarity.

3.4 Single-crystal X-ray structure analysis of $[K(thf)_{1.5}]_2[\mathbf{1}\cdot(\mu\text{-pz})]_2$

The ionic 1D-polymeric structure $[K(thf)_{1.5}]_2[\mathbf{1}\cdot(\mu\text{-pz})]_2$ crystallizes as a thf solvate in the triclinic space group $P\bar{1}$ (No. 2) (Figure S40a). Each K^+ cation binds to the phenyl rings of two neighboring anions through weak $K^+\cdots\pi(Ar)$ interactions. The coordination spheres of $K1^+$ and $K2^+$ are completed by one and two thf ligands, respectively. The polymer chains are therefore generated via contact-ion pair formation (Figure S40b).

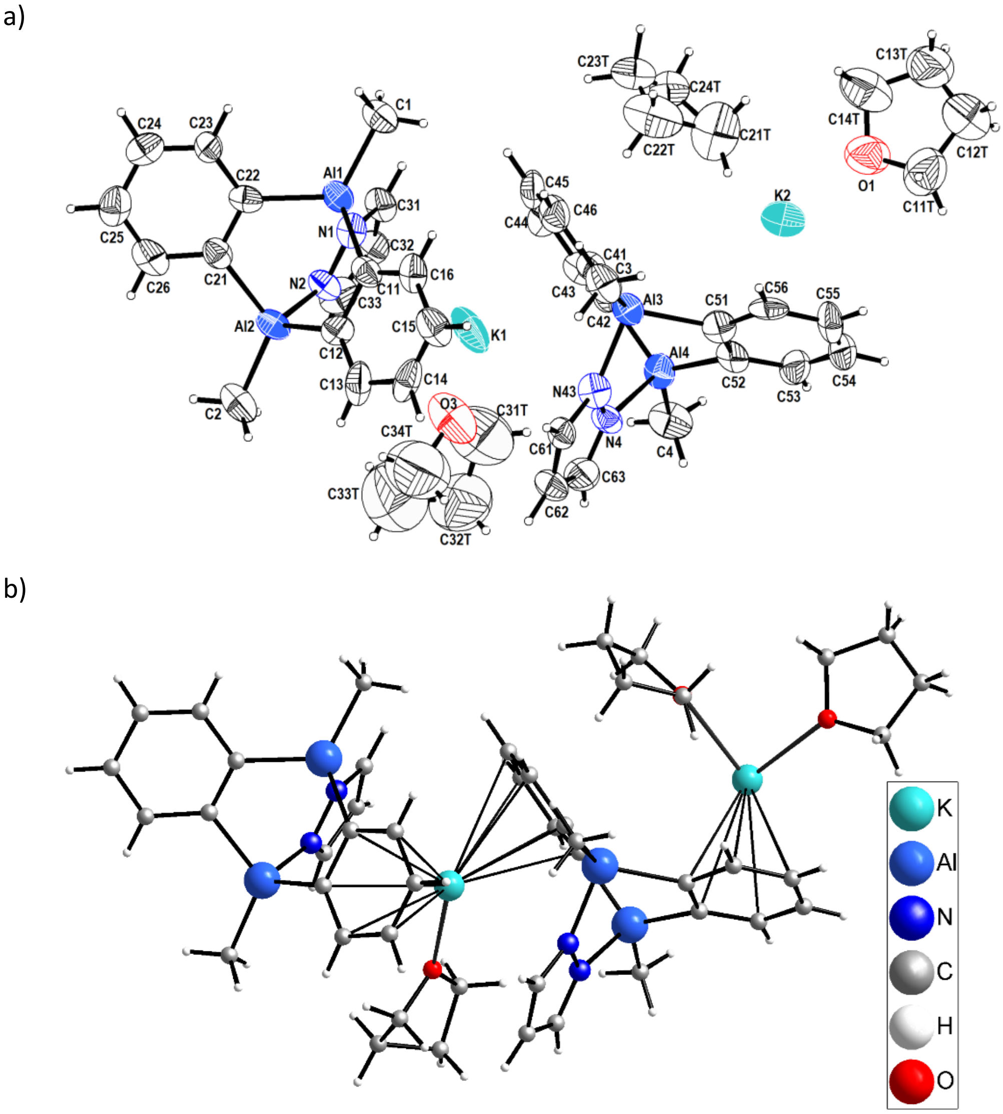

Figure S40: a) Molecular structure of $[K(thf)_{1.5}]_2[\mathbf{1}\cdot(\mu\text{-pz})]_2$ in the solid state. Atomic displacement ellipsoids are drawn at the 50 % probability level. b) Solid-state structure of $[K(thf)_{1.5}]_2[\mathbf{1}\cdot(\mu\text{-pz})]_2$ with $K^+\cdots\pi(Ar)$ interactions explicitly shown.

3.5 Single-crystal X-ray structure analysis of 1·(pyz)(thf)

Compound **1**·(pyz)(thf) crystallizes in the monoclinic space group $P2_1/n$ (No. 14) (Figure S41a). Al1 carries exclusively one pyz molecule; the ligand positions at Al2 are statistically occupied by pyz (33 %) and thf, which is itself disordered over two positions (67 % in total) (Figure S41b). Therefore, the composition of the crystal can be formulated as $(Al_2C_{22}H_{22}N_4)_{0.333}(Al_2C_{22}H_{26}N_2O)_{0.667}$. During the refinement, various SADI constraints were applied to the C–C distances in the disordered thf molecules.

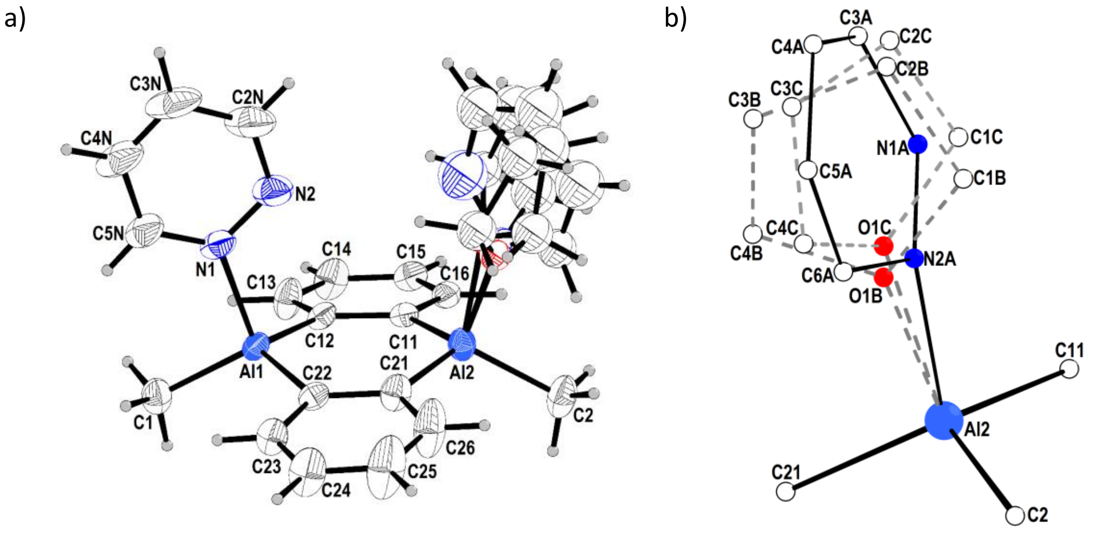

Figure S41: a) Molecular structure of **1**·(pyz)(thf) in the solid state. Atomic displacement ellipsoids are drawn at the 50 % probability level. b) Disorder of the coordinated ligands at Al2.

3.6 Single-crystal X-ray structure analysis of DAA-R_2·$(AlBrR_2)_2$ (R = Me or Br)

Compound DAA-R_2·$(AlBrR_2)_2$ (R = Me or Br) crystallizes in the triclinic space group $P\bar{1}$ (No. 2) with one crystallographically unique molecule at the inversion center (Figure S42). Me and Br are present with different proportions at positions C1B, Br3, and Br4. The position C1B is occupied by Me (73.4(3) %) and Br (26.6(3) %). The Br3 position is almost pure Br with only 4.4(3) % Me. The Br4 position is also predominantly brominated, with a Br/Me ratio of 83.0(3)/17.0(3) %. As a result, molecules with a different number of Me and Br groups coexist in the crystal.

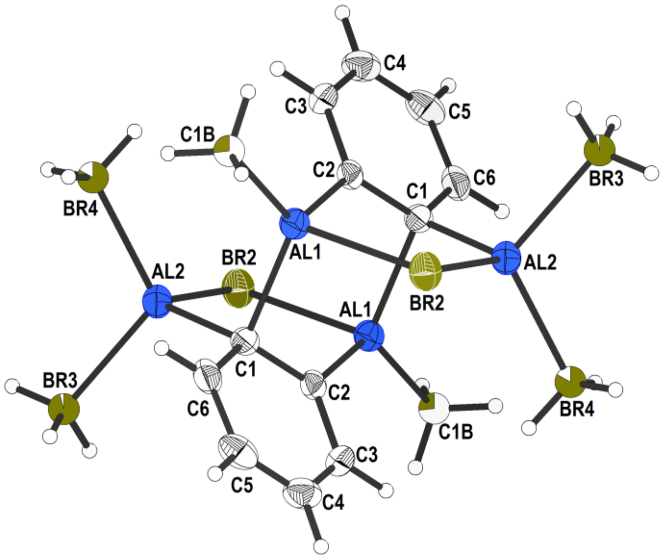

Figure S42: Molecular structure of DAA-R_2·$(AlBrR_2)_2$ (R = Me or Br) in the solid state. Atomic displacement ellipsoids are drawn at the 50 % probability level. The mixed sorted positions are displayed as two-colored sectors that are proportional to the corresponding location occupancy factors.

3.7 Single-crystal X-ray structure analyses of two polymorphous modifications of 2·$(AlBr_3)_2$

Compound **2**·$(AlBr_3)_2$ crystallizes in two polymorphous modifications. The relatively denser α form crystallizes in the triclinic space group $P\bar{1}$ (No. 2) with two crystallographically unique molecules at the inversion centers (Figure S43a). The β form also crystallizes in the triclinic space group $P\bar{1}$ (No. 2), but with one unique molecule at the inversion center (Figure S43b).

Analysis of the crystal packing of the polymorphous modifications of **2**·$(AlBr_3)_2$ shows significant differences: The molecules in the α-modification form a distorted face-centered cubic (f.c.c) packing (Figure S43c), while the molecular packing in the β form follows a distorted body-centered cubic (b.c.c.) motif (Figure S43d). However, the distances between the molecular centers are similar (α: 8.51···10.08 Å; β: 8.07···11.43 Å).

a)

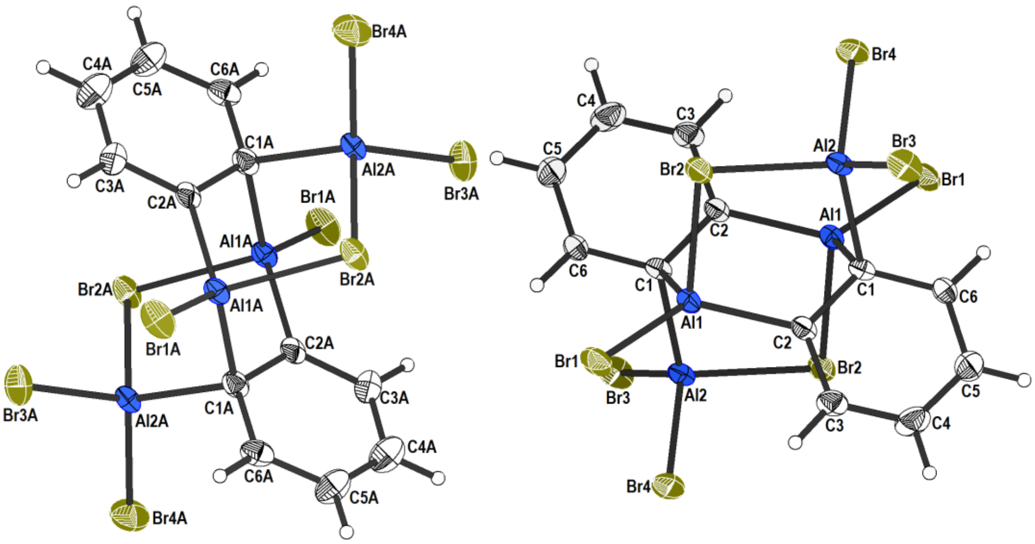

b)

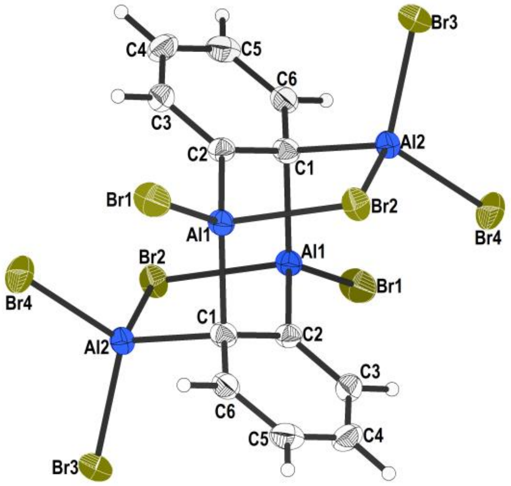

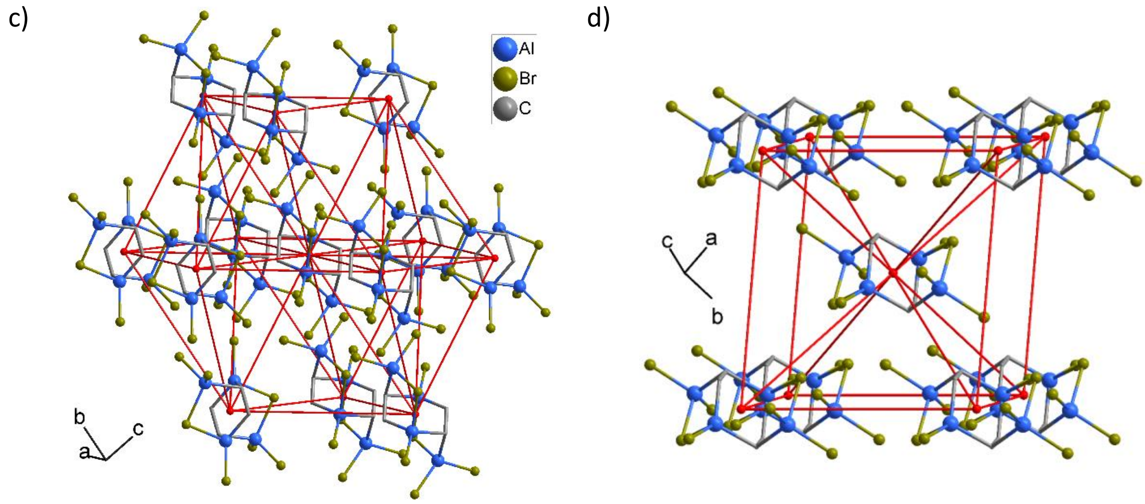

Figure S43: Molecular structure of a) the two unique molecules of α-**2**·$(AlBr_3)_2$ and b) the one unique molecule of β-**2**·$(AlBr_3)_2$. Atomic displacement ellipsoids are drawn at the 50 % probability level. Crystal packing in c) α-**2**·$(AlBr_3)_2$ and d) β-**2**·$(AlBr_3)_2$. Ph rings are omitted for clarity.

3.8 Single-crystal X-ray structure analysis of (2)$_2$

Compound (**2**)$_2$ crystallizes in the monoclinic space group *P*2/*n* (No. 13) with one crystallographically unique molecule at the two-fold axis, resulting in C_2 symmetry (Figure S44).

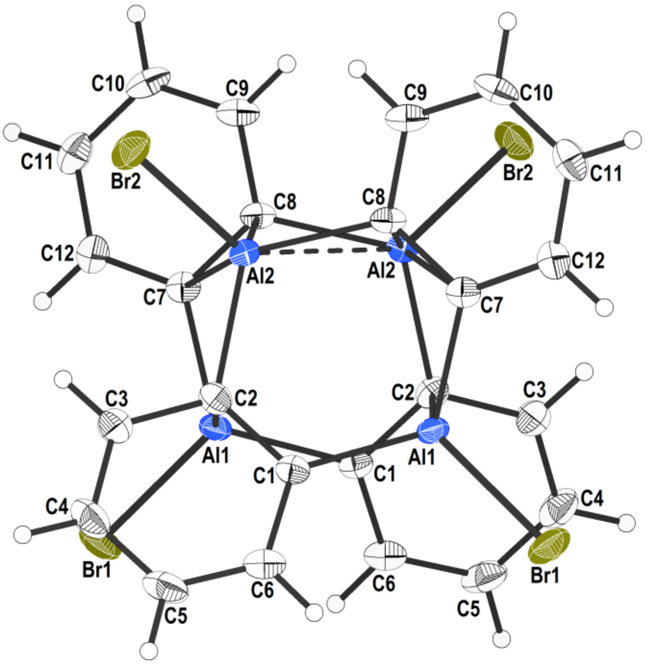

Figure S44: Molecular structure of (**2**)$_2$ in the solid state. Atomic displacement ellipsoids are drawn at the 50 % probability level.

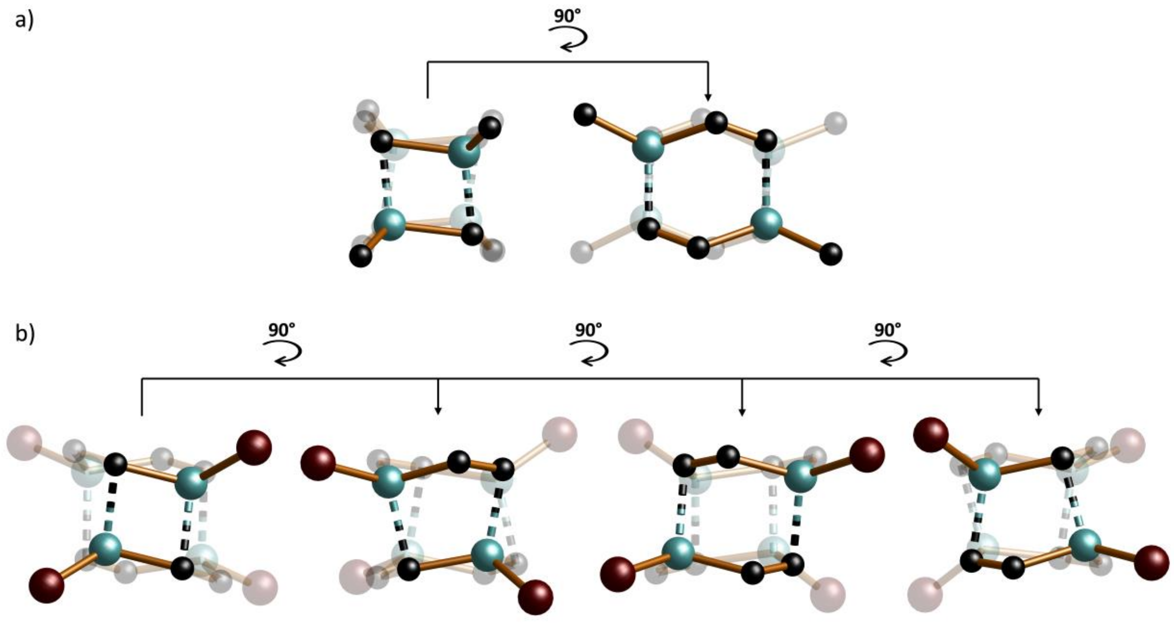

Figure S45: Comparison of the "belts" of the $(Al_2C_4)_2$ fragment of a) (**1**)$_2$ and b) (**2**)$_2$. H atoms omitted for clarity. C: black, Br: brown, Al: turquoise.

3.9 Single-crystal X-ray structure analysis of $2\cdot(OEt_2)_2$

Compound **2**·$(OEt_2)_2$ crystallizes in the monoclinic space group *C*2/*c* (No. 15) with one crystallographically unique molecule at the inversion center (Figure S46). The crystal appeared to be a pseudo-merohedral twin. The twinning matrix (–1 0 0 / 0 1 0 / 1 0 1 2) was calculated using the algorithm[S12] as: T = A × B × A^{-1}, where matrix A = (0 –1 0 / –1 0 0 / –1 0 –2) corresponds to the transformation of the *mC* unit cell into the pseudo-*oF* unit cell, and matrix B = (1 0 0 / 0 1 0 / 0 0 –1) corresponds to twinning by inversion along the *c* axis in the pseudo-*oF* setting. After applying the TWIN instruction, the R values dropped significantly, despite the relatively low weight of 3.26 % of the second twin domain.

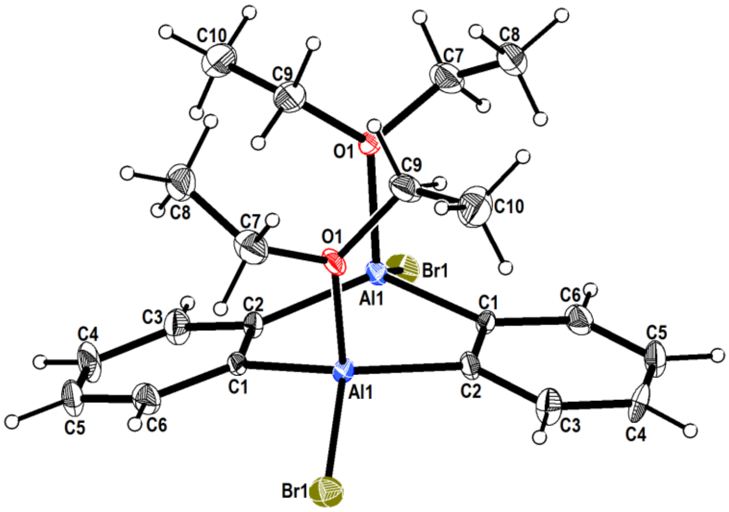

Figure S46: Molecular structure of **2**·$(OEt_2)_2$ in the solid state. Atomic displacement ellipsoids are drawn at the 50 % probability level.

3.10 Single-crystal X-ray structure analysis of 2·(thf)$_2$

Compound **2**·(thf)$_2$ crystallizes in the monoclinic space group $P2_1/n$ (No. 14) with two crystallographically unique molecules at the inversion centers (Figure S47).

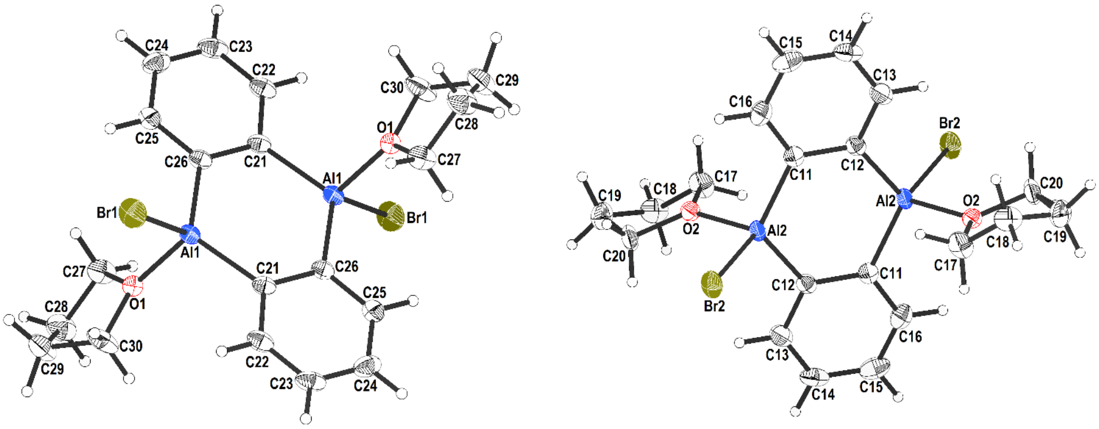

Figure S47: Molecular structures of the two unique molecules of **2**·(thf)$_2$ in the solid state. Atomic displacement ellipsoids are drawn at the 50 % probability level.

3.11 Single-crystal X-ray structure analysis of $\mathbf{2}\cdot(py)_2 \times C_6H_6$

The solvate $\mathbf{2}\cdot(py)_2$ crystallizes with one C_6H_6 molecule in the triclinic space group $P\bar{1}$ (No. 2) (Figure S48). Both molecules are located at inversion centers. The C_6H_6 molecule is disordered over two positions with almost equal weights.

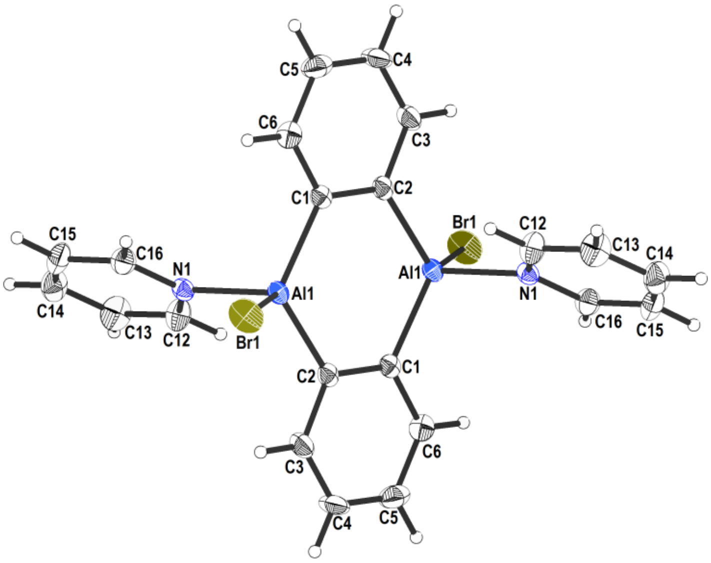

Figure S48: Molecular structure of $\mathbf{2}\cdot(py)_2 \times C_6H_6$ in the solid state. Atomic displacement ellipsoids are drawn at the 50 % probability level. The solvent molecule (C_6H_6) is omitted for clarity.

3.12 Single-crystal X-ray structure analysis of [*n*Bu₄N][3]

The ionic compound [nBu$_4$N][**3**] crystallizes in the chiral polar monoclinic space group $P2_1$ (No. 4); the cation and anion are both in general positions (Figure S49). The crystal packing is chiral, and the Flack parameter, which has been refined to a value statistically equal to zero, shows that the crystal is homochiral.

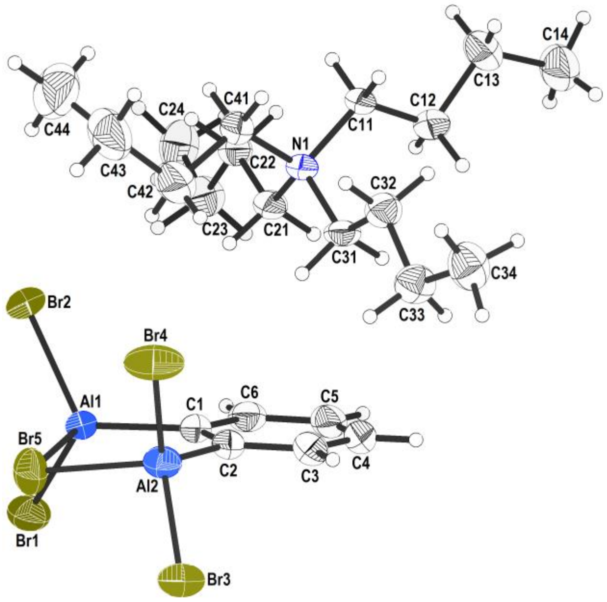

Figure S49: Molecular structure of [nBu$_4$N][**3**] in the solid state. Atomic displacement ellipsoids are drawn at the 50 % probability level.

4 X-ray powder diffraction

To determine the phase purity of the bulk material **2**·$(AlBr_3)_2$, X-ray powder diffraction was performed at room temperature and the measured diffractogram was compared with the simulated diffractogram from the single-crystal measurement. Data were collected in Debye-Scherrer geometry on a *STOE STADI-P* transmission diffractometer equipped with a curved Ge(111) monochromator and a *Dectris Mythen 1K* detector (PSD) and processed with the *STOE WinX^{POW}* software.[S13] Cu$K\alpha_1$ radiation (λ = 1.5406 Å) was used. The sample was placed in capillary mark tube made from borosilicate glass no. 50 (Ø = 1 mm) and sealed under an Ar atmosphere. The capillary was mounted on the diffractometer and rotated during the measurement. The 2θ range was 2–70°, moving the detector in steps of 0.5° (2θ) with an exposure time of 20 s/step.

4.1 X-ray powder diffractometry on 2·$(AlBr_3)_2$

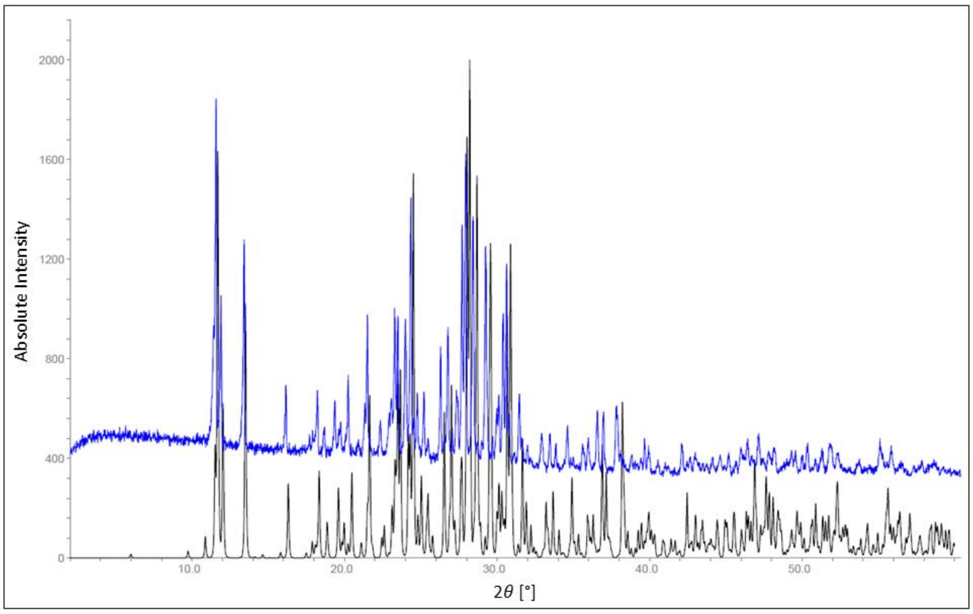

Figure S50: The recorded X-ray powder diffraction pattern (blue) and the predicted pattern based on the single-crystal X-ray diffraction data (black) of α-**2**·$(AlBr_3)_2$.

5 Computational details

All DFT calculations were performed using *Gaussian 16, Revision B.01*.[S14] IBO calculations were carried out with *IBOview*. Graphical representations of molecular geometries were produced with the *CYLview20* software.[S15]

Geometry optimizations and Hessian calculations were performed at the ωB97XD[S16]/def2-TZVPP[S17,S18] level of theory including implicit solvation by the solvent model based on density (SMD; solvent = C_6H_6, ε = 2.2706).[S19] Optimized geometries were confirmed to be the desired minimum energy structures or transition states by vibrational frequency analysis. Single-point calculations were performed at the SMD(C_6H_6)/ωB97XD/def2-QZVPP[S18] level. All free energy values were calculated for ambient temperature (298 K) and included a concentration correction[S20] accounting for the change in standard states going from gas phase to condensed phase. Bond orders were estimated using the Mayer bond order (MBO)[S21] and Wiberg bond index (WBI)[S22] methods. These were obtained with *Multiwfn 3.8*.[S23] For investigating the bonding situation of selected systems, additional calculations based on the intrinsic bond orbital (IBO)[S24] were performed.

5.1 Rearrangement processes in the dimer (2)$_2$

The observed transition states for the conformational changes in (**2**)$_2$ are quite small ($\Delta G^{\ddagger}$ = 2.1 and 3.0 kcal mol^{-1}; Scheme S1) and in the range as observed for C–C rotations in *e.g.* alkanes.[S25] Thus, these rearrangements seem to occur rapidly, which explains that only an averaged and thus symmetric structure is observed in the NMR spectra (in contrast to its solid state structure; *cf.* Figure S44). A monomer-dimer equilibrium seems unlikely since the dissociation [(**2**)$_2$ → 2 **2**] is endothermic with ΔG = 28.0 kcal mol^{-1}.

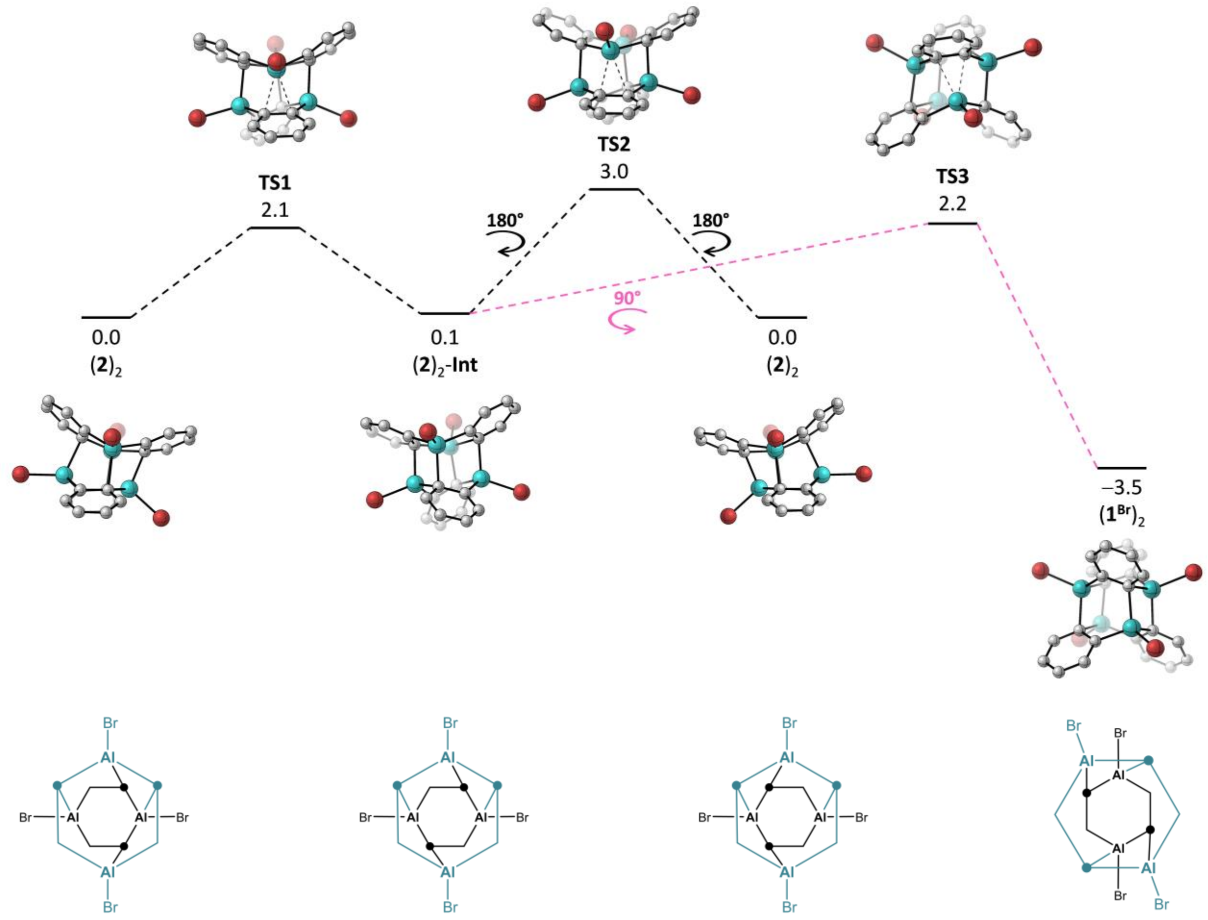

Scheme S1: Computed mechanism for the rearrangement in (**2**)$_2$ (H atoms omitted for clarity) with the corresponding schematic representations. The curved arrows indicate that the respective structures are rotated about the vertical axis by 180 ° or 90 °, respectively, for better visualization. *Notes*: a) Dimer (**2**)$_2$ is a chiral molecule; the transition from left to right generates the mirror image (= the other enantiomer). b) Although an energy difference of 0.9 kcal mol^{-1} is at the limit of computational accuracy, we note in passing that the lower energy of **TS1** compared to **TS2** corresponds with the differences in the computed WBI/MBO values of the respective bonds that are broken in the process (Figure S53).

5.2 Characterization of the bonding situation in (2)$_2$

The observed dimer formation in the case of (**2**)$_2$ raises the question of how to classify the interactions between the two DAA-Br_2 monomers M and M'. In our corresponding quantum-chemical calculations, no symmetry constraints were applied, although (**2**)$_2$ has C_2 symmetry in the solid state: Four short distances are present between Al atoms of one DAA-Br_2 monomer and *ipso*-C atoms of the other monomer (calculated distances: 2.120, 2.120, 2.250, 2.251 Å). As a result, (**2**)$_2$ has a cage structure, with six-membered rings serving as the base and top, and one four-membered, two five-membered, and one six-membered ring(s) constituting the belt (Figure S51). These ring sizes will serve as a guide in the following discussion of dimer interactions. To this end, we performed IBO calculations and computed the underlying WBIs and MBOs.

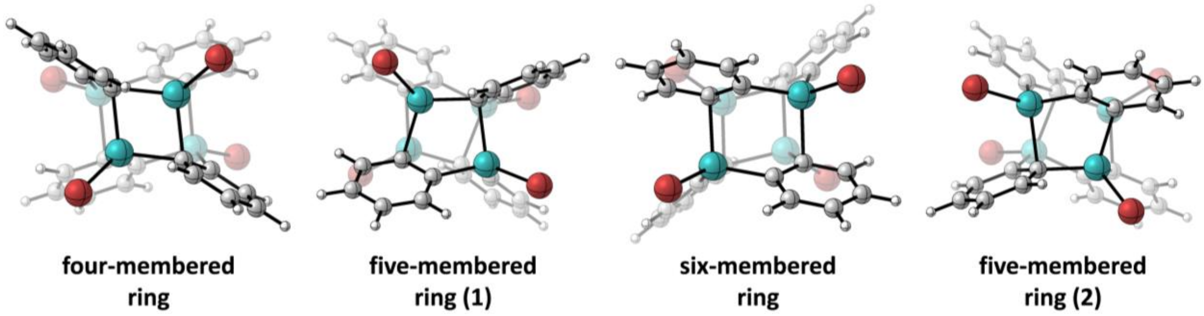

Figure S51: The vertical lines represent the shortest monomer···monomer distances in (**2**)$_2$; the different rings constituting the 'belt' of the dimer are also shown.

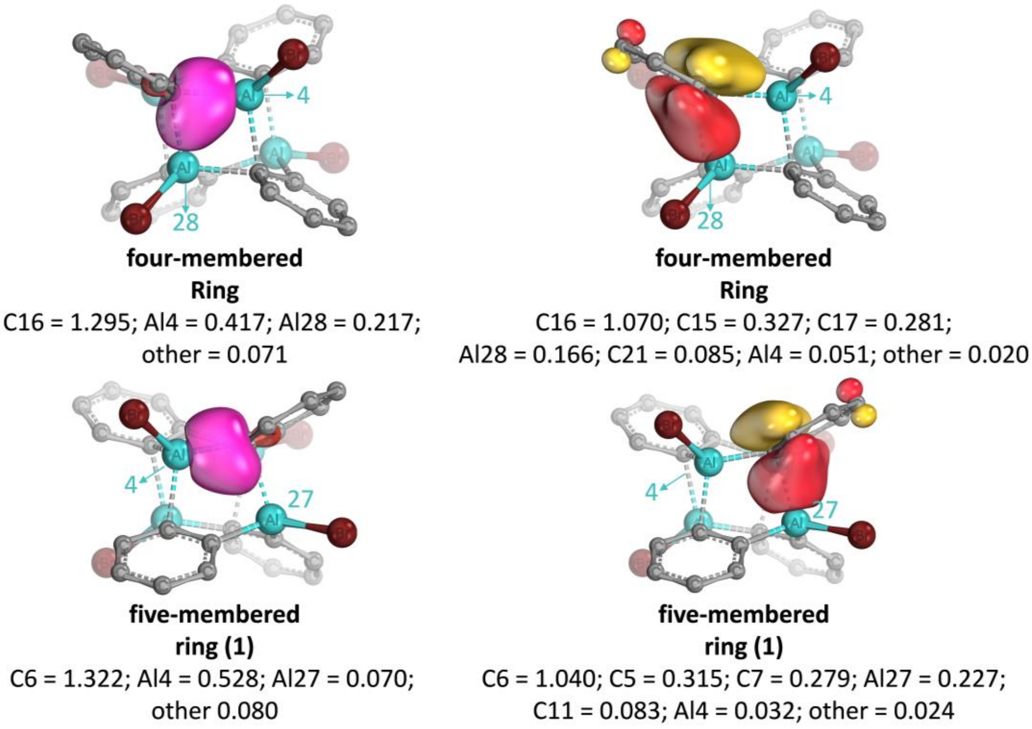

Figure S52: Characteristic intrinsic bond orbitals (IBOs) contributing to the two different kinds of interactions between the two DAA-Br_2 monomers within the four-membered ring (*top*) and within a five-membered ring (*bottom*). The numerical values listed are the total contributions related to the fraction of electrons of the doubly occupied IBO that are assigned to each individual atom.

To simplify the analysis of intermonomer interactions in (**2**)$_2$, we will treat each bridging C atom (C_b) as sp^2 hybridized, disregarding any potential contributions from Wheland-type[S26] electronic structures with sp^3-hybridized C_b atoms. In this framework, an Al atom of monomer M can interact with monomer

M' either via the unhybridized p_z orbital of C_b (Al···π(Ar) interaction) or via the Al–C_b σ bond (two-electron–three-center (2e3c) bond). Among the four M···M' interactions in (**2**)$_2$, two are pairwise identical (assuming an idealized C_2-symmetric dimer, as observed in the crystal lattice of (**2**)$_2$). The two remaining distinct types of interactions are shown in Figure S52. Both interactions involve contributions from Al···π(Ar) and 2e3c interactions, though two different extents: the interaction within the four-membered ring (Figure S52, top) exhibits a higher degree of 2e3c bonding (cf. Al28 = 0.217 vs. 0.166), while the interaction within the five-membered ring (Figure S52, bottom) is primarily of the Al···π(Ar) type (cf. total contribution to the IBO of Al27 = 0.070 vs. 0.227).

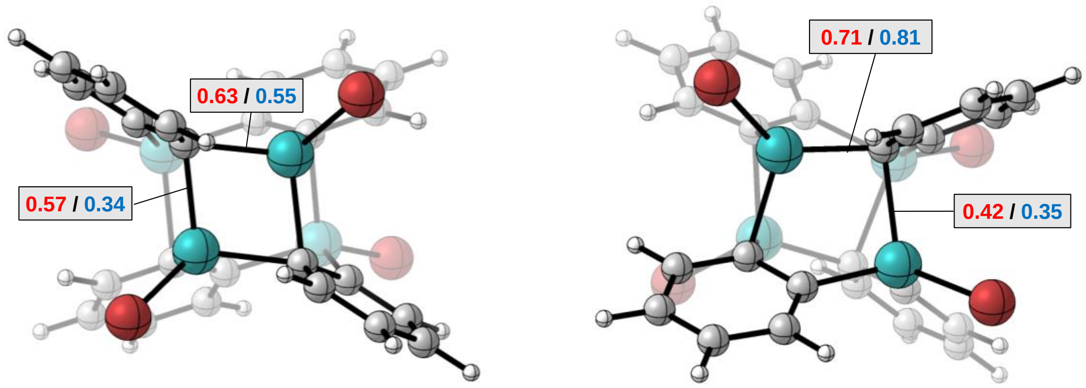

four-membered ring

five-membered ring (1)

Figure S53: Wiberg bond indices (WBIs; red) and Mayer bond orders (MBOs; blue) of selected interactions between both DAA-Br_2 monomers. *Left:* WBIs and MBOs computed for the four-membered ring. *Right:* WBIs and MBOs computed for the five-membered ring. Level of theory: SMD(C_6H_6)/ωB97XD/def2-TZVPP//SMD(C_6H_6)/ωB97XD/def2-TZVPP.

WBIs/MBOs of selected M···M' interactions in (**2**)$_2$ are shown in Figure S53. Higher WBIs/MBOs indicate stronger bonds. First, we find that the *intra*monomer Al–C_b bonds are stronger than the *inter*monomer interactions. Second, the *inter*monomer (mainly) 2e3c-type bond within the four-membered ring tends to be stronger than the (mainly) Al···π(Ar)-type bond within the five-membered ring (compare also the different energies of **TS1** und **TS2** in Scheme S1).

5.3 Computed structures and free energies

(**2**)$_2$
***G*$_{298}$** = –12191.256303 Hartree

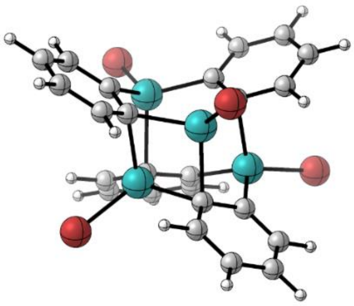

TS1
***G*$_{298}$** = –12191.252957 Hartree

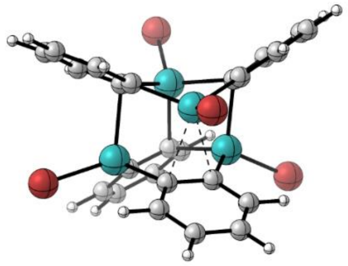

(**2**)$_2$-**Int**
***G*$_{298}$** = –12191.256129 Hartree

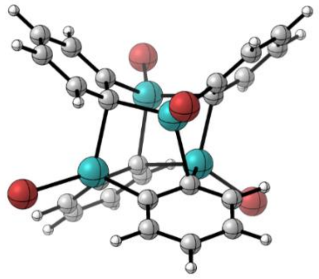

TS2
***G*$_{298}$** = –12191.251533 Hartree

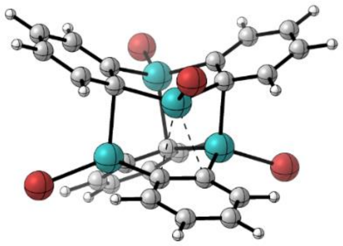

2
***G*$_{298}$** = –6095.605875 Hartree

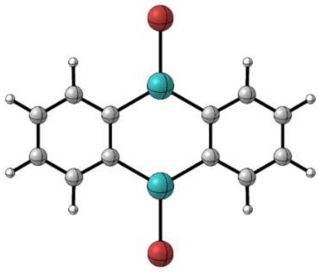

TS3
***G*$_{298}$** = –12191.252852 Hartree

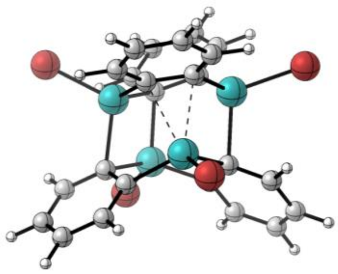

(**1**^{Br})$_2$
***G*$_{298}$** = –12191.261830 Hartree

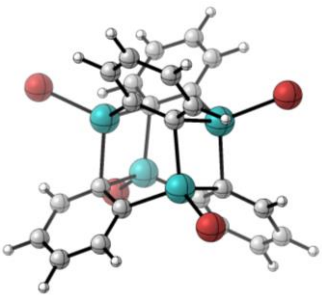

(**1**)$_2$
***G*$_{298}$** = –2053.446186 Hartree

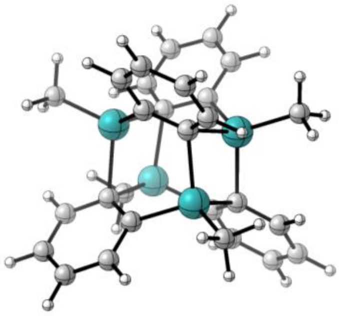

(**2**$^{\mathbf{Me}}$)$_2$

$\boldsymbol{G_{298}}$ = –2053.438377 Hartree

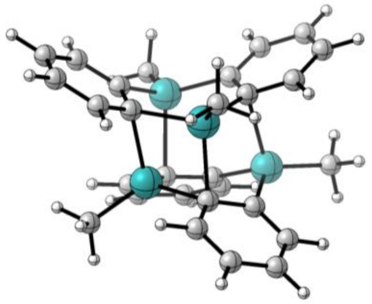

6 References

S1 J. J. Eisch and B. W. Kotowicz, *Eur. J. Inorg. Chem.*, 1998, 761–769.

S2 N. Dordević and H. V. Huynh, *Organometallics*, 2022, **41**, 335–344.

S3 G. R. Fulmer, A. J. M. Miller, N. H. Sherden, H. E. Gottlieb, A. Nudelman, B. M. Stoltz, J. E. Bercaw and K. I. Goldberg, *Organometallics*, 2010, **29**, 2176–2179.

S4 We are aware that the compounds $AlMe_3$, $AlBr_3$, and Me_nAlBr_{3-n} are monomeric neither in solution nor in the solid state. However, for simplicity, the monomeric forms were used in calculating the quantities employed.

S5 M. A. Dam, T. Nijbacker, F. J. J. De Kanter, O. S. Akkerman, F. Bickelhaupt and A. L. Spek, *Organometallics*, 1999, **18**, 1706–1709.

S6 E. Januszewski, A. Lorbach, R. Grewal, M. Bolte, J. W. Bats, H.-W. Lerner and M. Wagner, *Chem. Eur. J.*, 2011, **17**, 12696–12705.

S7 A. Lorbach, C. Reus, M. Bolte, H.-W. Lerner and M. Wagner, *Adv. Synth. Catal.*, 2010, **352**, 3443–3449.

S8 H. F. Bettinger and M. Filthaus, *J. Org. Chem.*, 2007, **72**, 9750–9752.

S9 G. M. Sheldrick, *Acta Crystallogr. Sect. A*, 2015, **71**, 3–8.

S10 G. M. Sheldrick, *Acta Crystallogr. Sect. C*, 2015, **71**, 3–8.

S11 V. A. Blatov, A. P. Shevchenko and D. M. Proserpio, *Cryst. Growth Des.*, 2014, **14**, 3576–3586.

S12 R. Herbst-Irmer and G. M. Sheldrick, *Acta Crystallogr. Sect. B*, 1998, **54**, 443–449.

S13 *WinX*^{POW}; Stoe & Cie.: Darmstadt, Germany, 2011.

S14 Gaussian 16, Revision B.01, M. J. Frisch, G. W. Trucks, H. B. Schlegel, G. E. Scuseria, M. A. Robb, J. R. Cheeseman, G. Scalmani, V. Barone, B. Mennucci, G. A. Petersson, H. Nakatsuji, M. Caricato, X. Li, H. P. Hratchian, A. F. Izmaylov, J. Bloino, G. Zheng, J. L. Sonnenberg, M. Hada, M. Ehara, K. Toyota, R. Fukuda, J. Hasegawa, M. Ishida, T. Nakajima, Y. Honda, O. Kitao, H. Nakai, T. Vreven, J., A. Montgomery Jr., J. E. Peralta, F. Ogliaro, M. Bearpark, J. J. Heyd, E. Brothers, K. N. Kudin, V. N. Staroverov, R. Kobayashi, J. Normand, K. Raghavachari, A. Rendell, J. C. Burant, S. S. Iyengar, J. Tomasi, M. Cossi, N. Rega, J. M. Millam, M. Klene, J. E. Knox, J. B. Cross, V. Bakken, C. Adamo, J. Jaramillo, R. Gomperts, R. E. Stratmann, O. Yazyev, A. J. Austin, R. Cammi, C. Pomelli, J. W. Ochterski, R. L. Martin, K. Morokuma, V. G. Zakrzewski, G. A. Voth, P. Salvador, J. J. Dannenberg, S. Dapprich, A. D. Daniels, Ö. Farkas, J. B. Foresman, J. V Ortiz, J. Cioslowski and D. J. Fox, Gaussian, Inc., Wallingford, CT, USA, 2016.

S15 C. Y. Legault, *CYLview*, 1.0b, 2009.

S16 J.-D. Chai and M. Head-Gordon, *Phys. Chem. Chem. Phys.*, 2008, **10**, 6615–6620.

S17 (*a*) A. Schäfer, H. Horn and R. Ahlrichs, *J. Chem. Phys.*, 1992, **97**, 2571–2577; (*b*) A. Schäfer, C. Huber and R. Ahlrichs, *J. Chem. Phys.*, 1994, **100**, 5829–5835; (*c*) K. Eichkorn, F. Weigend, O. Treutler and R. Ahlrichs, *Theor. Chem. Acc.*, 1997, **97**, 119–124.

S18 F. Weigend, F. Furche and R. Ahlrichs, *J. Chem. Phys.*, 2003, **119**, 12753–12762.

S19 A. V. Marenich, C. J. Cramer and D. G. Truhlar, *J. Phys. Chem. B*, 2009, **113**, 6378–6396.

S20 (*a*) C. P. Kelly, C. J. Cramer and D. G. Truhlar, *J. Chem. Theory Comput.*, 2005, **1**, 1133–1152; (*b*) M. Sparta, C. Riplinger and F. Neese, *J. Chem. Theory Comput.*, 2014, **10**, 1099–1108.

S21 A. J. Bridgeman, G. Cavigliasso, L. R. Ireland and J. Rothery, *Dalton Trans.*, 2001, 2095–2108.

S22 K. B. Wiberg, *Tetrahedron*, 1968, **24**, 1083–1096.

S23 T. Lu and F. Chen, *J. Comput. Chem.*, 2012, **33**, 580–592.

S24 G. Knizia, *J. Chem. Theory Comput.*, 2013, **9**, 4834–4843.

S25 K. B. Wiberg and M. A. Murcko, *J. Am. Chem. Soc.*, 1988, **110**, 8029–8038.

S26 C. A. Reed, K.-C. Kim, E. S. Stoyanov, D. Stasko, F. S. Tham, L. J. Mueller and P. D. W. Boyd, *J. Am. Chem. Soc.*, 2003, **125**, 1796–1804.
